# Stereodivergent approach in the protected glycal synthesis of L-vancosamine, L-saccharosamine, L-daunosamine and L-ristosamine involving a ring-closing metathesis step

**DOI:** 10.3762/bjoc.14.274

**Published:** 2018-11-29

**Authors:** Pierre-Antoine Nocquet, Aurélie Macé, Frédéric Legros, Jacques Lebreton, Gilles Dujardin, Sylvain Collet, Arnaud Martel, Bertrand Carboni, François Carreaux

**Affiliations:** 1 Univ Rennes, CNRS, ISCR (Institut des Sciences Chimiques de Rennes), UMR 6226, 263 avenue du Général Leclerc, Campus de Beaulieu, F-35000 Rennes, France; 2 Institut des Molécules et Matériaux du Mans, UMR 6283 CNRS-Université du Maine, avenue Olivier Messiaen, 72085 Cedex Le Mans, France; 3 Chimie Et Interdisciplinarité: Synthèse, Analyse, Modélisation (CEISAM), UMR 6230 CNRS-Université de Nantes, 2 chemin de la Houssinière, 44322 Cedex Nantes, France

**Keywords:** 3-amino glycals, diastereoselective additions to aldehydes, pluramycins, ring-closing metathesis, vinyl ethers

## Abstract

In this paper, a new access to several chiral 3-aminoglycals as potential precursors for glycosylated natural products is reported from a common starting material, (−)-methyl-L-lactate. The stereodivergent strategy is based on the implementation of a ring-closing metathesis of vinyl ethers as key step of reaction sequences developed.

## Introduction

**Figure 1:**
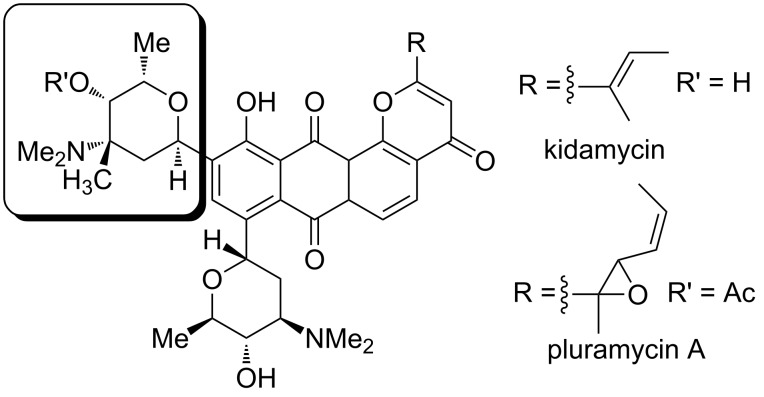
*N*,*N*-Dimethyl-L-vancosamine as substructure of kidamycin and pluramycin.

Several classes of medicinally useful molecules with antibiotic and anticancer activity contain in their structures 3-amino-2-deoxy sugars [1]. For instance, *N*,*N*-dimethyl-L-vancosamine is an essential component of pluramycin antibiotics such as kidamycin and pluramycin A via a C-glycosidic linkage (Figure 1).

**Figure 2:**
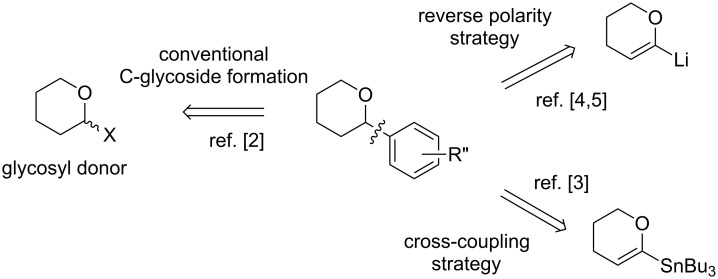
Glycals as relevant scaffolds for constructing aryl C-glycosidic linkage.

For constructing aryl C-glycoside bonds, glycal derivatives are versatile synthetic intermediates (Figure 2). Indeed, they can be converted into glycosyl donors but can also be considered as potential coupling partners or nucleophilic moieties via the formation of transient metalated species [2]. As example concerning their use in pluramycins' syntheses, an approach to the synthesis of pluraflavin A was developed based on a Stille coupling to install the C-linked sugar residue [3]. Moreover, the addition of lithiated glycals to quinone derivatives followed by a rearrangement was also studied for the synthesis of kidamycin according to a “reverse polarity” strategy [4–5].

Considering that the glycal scaffolds are versatile building blocks with multiple applications in the field of natural product synthesis [6], the development of new asymmetric synthetic sequences with stereochemical diversity is still of high interest. Different approaches have been reported for the asymmetric synthesis of protected 3-aminoglycals from non-carbohydrate precursors. Most of them used a common methodology for the construction of the pyranosyl glycal ring which is based on a cycloisomerization reaction of chiral homopropargylic alcohols [7–10]. In some cases, the strategy used for the preparation of the corresponding alkynyl alcohols requires the handling of toxic tin reagents [8–9].

**Figure 3:**
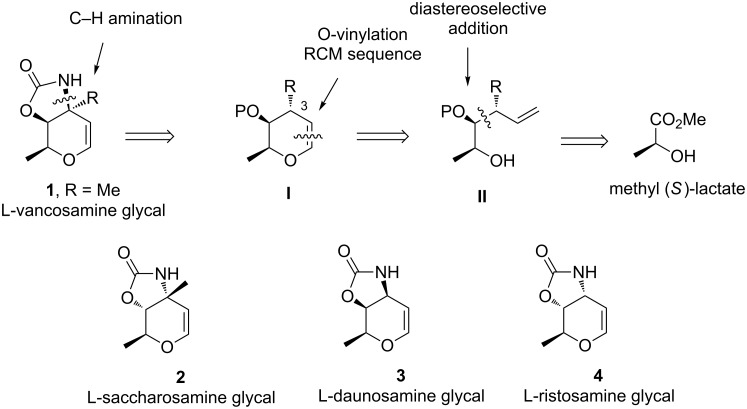
Strategy including a ring-closing metathesis of vinyl ethers as key step for the preparation of several carbamate-protected 3-aminoglycals.

During these last years, ring-closing metathesis (RCM) of vinyl ethers have proved to be an efficient method for the preparation of chiral glycal scaffolds [11–18] as demonstrated in some total syntheses of marine polycyclic ethers [19–21]. However, to the best of our knowledge, this methodology was never evaluated for the synthesis of this kind of nitrogen-containing substrates. Taking into account our interest about the development of new synthetic approaches to pluramycins [22–23], we speculated that the cyclic vinyl ether derivative **I**, with the prerequisite configuration of all stereogenic centers of the carbamate-protected glycal of L-vancosamine **1**, could be obtained from the alcohol derivative **II** using an O-vinylation–ring-closing metathesis sequence (Figure 3). Afterwards, the introduction of nitrogen in the convenient position (C3) could be performed by a stereopecific nitrene insertion reaction catalyzed by rhodium(II) complexes [24–25].

Herein, we describe our outcomes related to the implementation of this strategy for the synthesis of L-vancosamine derivative **1**, as well as its diastereoisomer, the carbamate-protected 3-aminoglycal of L-saccharosamine **2**, employing the (*S*)-(−)-methyl lactate as common starting material. The efficiency and generality of this methodology was also demonstrated by a new synthesis of C-3 unbranched amino glycals, L-daunosamine **3** and L-ristosamine **4** derivatives, from the same source of chirality.

## Results and Discussion

**Scheme 1:**
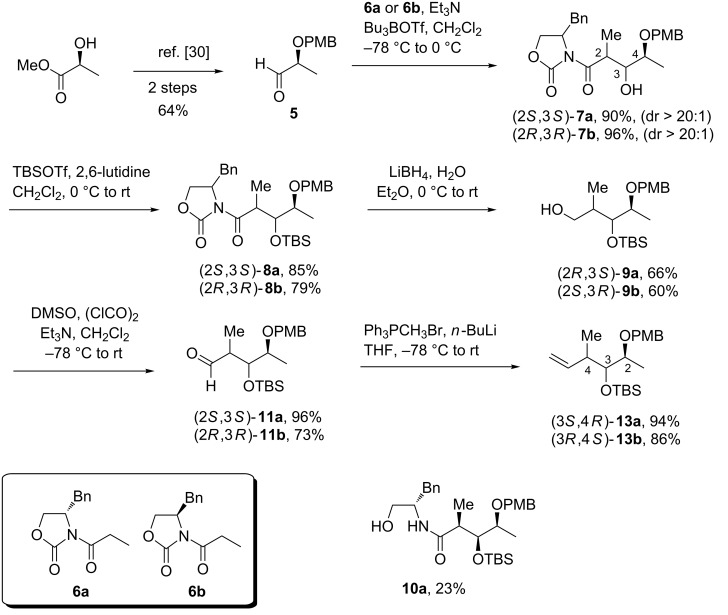
Evans aldol reaction for the preparation of diastereomeric compounds **13a** and **13b**.

**Synthesis of vancosamine and saccharosamine glycals.** The chiral (−)-lactic methyl ester was identified as the privileged starting material considering that the Evans aldol reaction via boron enolates [26–28] with an appropriately O-protected aldehyde should afford the desired aldol adduct with a *syn* relative configuration between the two newly created chiral centers [29–30]. Moreover, the boron-mediated stereoselective aldol reaction is all the more interesting for our synthetic plan as stereochemical diversity can be generated depending on the absolute configuration of the chiral auxiliary used. The aldehyde **5** was first prepared according to a described procedure in two steps from methyl L-lactate (Scheme 1) [31]. The reaction with (*R*)- or (*S*)-oxazolidinones **6** led to the formation of 2,3-*syn* aldol products **7** in good yields with a very high level of diastereoselection (>20:1 for both).

**Scheme 2:**
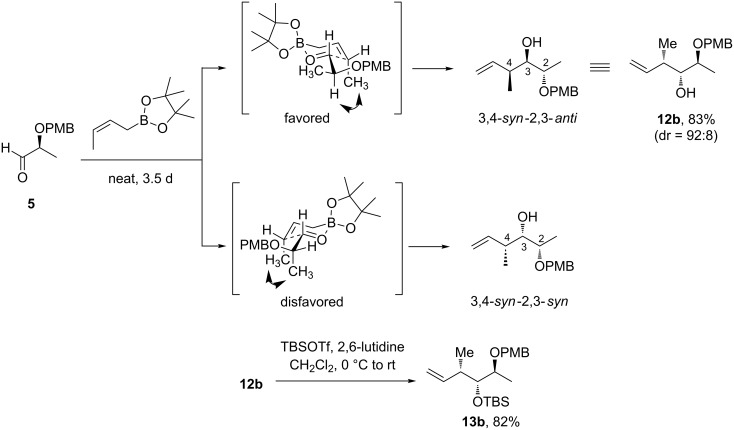
Alternative preparation of **13b** based on a diastereoselective allylboration.

After protection of the free hydroxy group, the reduction of the *N*-acyl oxazolidinones **8** into primary alcohols **9** was accomplished by LiBH_4_ in presence of water or LiAlH_4_ [32]. Whatever the conditions used for this step, moderate yields were obtained for the desired products due to the formation of substantial amounts of ring-opened byproducts **10** resulting from the hydride addition to the carbonyl group of the oxazolidinone ring [33–34]. The alcohols **9** were then subjected to a Swern oxidation followed by a Wittig reaction to generate the corresponding alkenes **13a**,**b** in 90% and 63% yield, respectively, over two steps. Alternatively, we envisioned that, from the same α-substituted chiral aldehyde **5**, compound **13b** could be obtained in a more straightforward manner employing a strategy based on a diastereoselective allylboration reaction (Scheme 2) [35]. Indeed, the reaction of achiral pinacol (*Z*)-crotylboronate with **5** under neat conditions at room temperature gave a good level of diastereoselectivity for the hitherto unreported 3,4-*syn*-2,3-*anti* product **12b** [36–39].

The *syn* relationship between C3 and C4 is controlled by the (*Z*)-geometry of the crotylboronate, while the 2,3-*anti* relationship can be rationalized by invoking Cornforth-like transition states [40–43]. Eventually, silylation of the homoallylic alcohol **12b** afforded the expected compound **13b** in 68% overall yield from **5** after purification, compared to 29% using a strategy based on an Evans’ aldol reaction.

**Scheme 3:**
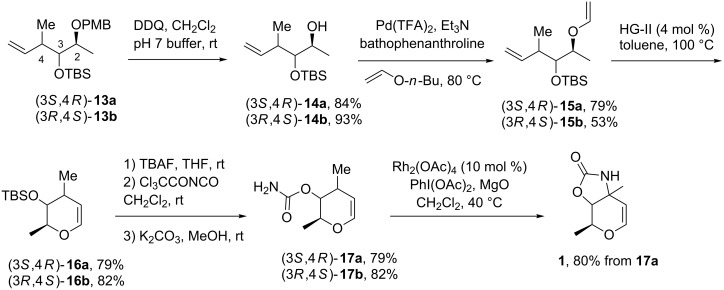
O-Vinylation-ring-closing metathesis sequence for access to 3-amino glycals.

Mildly oxidizing conditions using 2,3-dichloro-5,6-dicyano-1,4-benzoquinone (DDQ) were used for the removal of the *p*-methoxybenzyl (PMB) group to provide alcohols **14** (Scheme 3). Several palladium(II) catalysts have been tested for the conversion of alcohols to vinyl ethers **15** [13,44–46]. We found that the best yields were obtained using Pd(TFA)_2_ and *n*-butyl vinyl ether as solvent in the presence of bathophenanthroline as ligand. In the case of Pd(OAc)_2_, the reaction was slower with moderate yields.

The ring-closing metathesis reaction was performed with Hoveyda–Grubbs second-generation (HG-II) catalyst to deliver the corresponding dihydropyrans **16** in excellent yields given that this kind of reaction can be sensitive to the substitution pattern contained in the substrate [11]. After silyl deprotection, the key C–H amination precursors **17a**,**b** for the synthesis of the carbamate-protected glycal of L-vancosamine **1** and L-saccharosamine **2** were prepared in two steps by treatment of alcohols with the trichloroacetyl isocyanate reagent (TCA-NCO) followed by basic hydrolysis. The spectroscopic properties of carbamates **17** were identical to those reported in the literature [7–8]. Although the intramolecular C–H amination of compounds **17** under the Du Bois conditions [24] was already described in the literature [8–9], the reaction was nevertheless achieved with carbamate **17a** in order to check the reproducibility of the final step. As expected, L-vancosamine glycal **1** was obtained in similar yield than one reported [8,47].

**Scheme 4:**
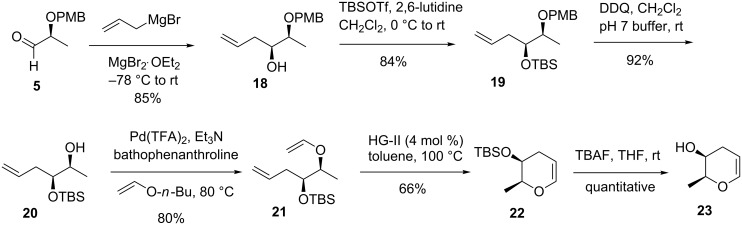
Synthesis of key intermediate **23** for the C-3 unbranched amino glycals preparation.

**Synthesis of daunosamine and ristosamine glycals.** As previously, the chiral pool material **5** was used for this unbranched glycal synthesis (Scheme 4). The first step was the chelation-controlled addition of allylmagnesium bromide to **5** to provide the *syn* diastereomer **18** in high stereoselectivity (93:7). After silylation of the free hydroxy group, the cleavage of the PMB ether with DDQ led to alcohol **20** in 77% yield for the two steps. Ring-closing metathesis of diene **21**, obtained by O-vinylation of **20**, gave the dihydropyran **22** in 53% overall yield for two steps**.** The silyl group of compound **22** was cleaved using tetrabutylammonium fluoride (TBAF) in THF to form alcohol **23** which was used directly in the next step [48].

**Scheme 5:**
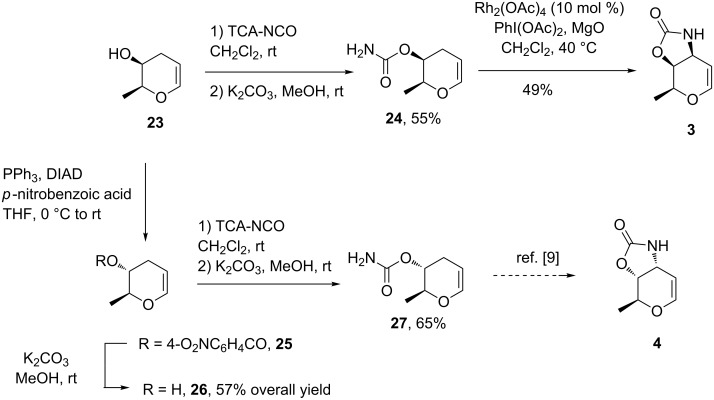
Access to diastereoisomeric compounds **3** and **4** from **23**.

At this stage, we envisioned that **23** could be also a key intermediate to access the ristosamine derivative by reversing the configuration of the stereogenic center bearing the hydroxy group (Scheme 5). With this in mind, the secondary alcohol **23** was engaged in a Mitsunobu reaction using *p*-nitrobenzoic acid as nucleophile to afford the expected compound **25**. Hydrolysis of the ester was achieved using potassium carbonate in methanol to afford the epimeric product **26**.

Both diastereomers **23** and **26** were converted to the corresponding known carbamates using a two step sequence. Reaction with TCA-NCO followed by a basic hydrolysis provided the desired compounds **24** and **27** in good yields and in full agreement with all reported spectroscopic data [9]. As an example, the expected protected glycal of L-daunosamine **3** [9,47] was obtained by regioselective rhodium nitrene insertion thus demonstrating the usefulness of this strategy for the synthesis of such compounds.

## Conclusion

We developed an alternative route to 3-aminoglycals through ring-closing metathesis of vinyl ethers as key step in the synthesis and using a common noncarbohydrate starting material. The approach was first validated for the synthesis of protected L-vancosamine glycal and extended afterwards to prepare a diastereomeric compound as well as other unbranched C-3 aminoglycals. The use of these synthons in the synthesis of glycosylated antibiotics as kidamycin is underway in our laboratory.

## Supporting Information

Supporting Information contains detailed experimental procedures with full characterization of all compounds and NMR spectra.

File 1 Experimental part and NMR spectra of all compounds.

## References

[1] Hauser F M, Ellenberger S R (1986). Chem Rev.

[2] Kitamura K, Ando Y, Matsumoto T, Suzuki K (2018). Chem Rev.

[3] Hartung J, Wright B J D, Danishefsky S J (2014). Chem – Eur J.

[4] Parker K A (1994). Pure Appl Chem.

[5] Parker K A, Koh Y-h (1994). J Am Chem Soc.

[6] Danishefsky S J, Bilodeau M T (1996). Angew Chem, Int Ed Engl.

[7] Cutchins W W, McDonald F E (2002). Org Lett.

[8] Parker K A, Chang W (2003). Org Lett.

[9] Parker K A, Chang W (2005). Org Lett.

[10] Fei Z, McDonald F E (2007). Org Lett.

[11] Sturino C F, Wong J C Y (1998). Tetrahedron Lett.

[12] Gurjar M K, Krishna L M, Reddy B S, Chorghade M S (2000). Synthesis.

[13] Peczuh M W, Snyder N L (2003). Tetrahedron Lett.

[14] Postema M H D, Piper J L, Liu L, Shen J, Faust M, Andreana P (2003). J Org Chem.

[15] Adam J-M, de Fays L, Laguerre M, Ghosez L (2004). Tetrahedron.

[16] Sharma H, Santra S, Debnath J, Antonio T, Reith M, Dutta A (2014). Bioorg Med Chem.

[17] Sutton A E, Seigal B A, Finnegan D F, Snapper M L (2002). J Am Chem Soc.

[18] Schmidt B, Biernat A (2008). Chem – Eur J.

[19] Majumder U, Cox J M, Johnson H W B, Rainier J D (2006). Chem – Eur J.

[20] Osei Akoto C, Rainier J D (2008). Angew Chem, Int Ed.

[21] Clark J S, Romiti F, Sieng B, Paterson L C, Stewart A, Chaudhury S, Thomas L H (2015). Org Lett.

[22] Mabit T, Siard A, Pantin M, Zon D, Foulgoc L, Sissouma D, Guingant A, Mathé-Allainmat M, Lebreton J, Carreaux F (2017). J Org Chem.

[23] Mabit T, Siard A, Legros F, Guillarme S, Martel A, Lebreton J, Carreaux F, Dujardin G, Collet S (2018). Chem – Eur J.

[24] Espino C G, Du Bois J (2001). Angew Chem, Int Ed.

[25] Du Bois J (2011). Org Process Res Dev.

[26] Evans D A, Vogel E, Nelson J V (1979). J Am Chem Soc.

[27] Evans D A, Nelson J V, Vogel E, Taber T R (1981). J Am Chem Soc.

[28] Mukaiyama T, Inoue T (1976). Chem Lett.

[29] Cowden C J, Paterson I (1997). Org React.

[30] Zhang Z, Collum D B (2017). J Org Chem.

[31] Roush W R, Bennett C E, Roberts S E (2001). J Org Chem.

[32] Heravi M M, Zadsirjan V, Farajpour B (2016). RSC Adv.

[33] Penning T D, Djuric S W, Haack R A, Kalish V J, Miyashiro J M, Rowell B W, Yu S S (1990). Synth Commun.

[R34] 34Compound **10b** was not isolated.

[35] Lachance H, Hall D G, Denmark S E (2008). Allylboration of carbonyl compounds. Organic reactions.

[36] Hoffmann R W, Weidmann U (1985). Chem Ber.

[37] Brinkmann H, Hoffmann R W (1990). Chem Ber.

[38] Wuts P G M, Bigelow S S (1988). J Org Chem.

[39] Roush W R, Adam M A, Walts A E, Harris D J (1986). J Am Chem Soc.

[40] Cornforth J W, Cornforth R H, Mathew K K (1959). J Chem Soc.

[41] Roush W R, Helmchen G, Hoffmann R W, Mulzer J (1995). Houben-Weyl, Stereoselective Synthesis.

[42] Cee V J, Cramer C J, Evans D A (2006). J Am Chem Soc.

[43] Díaz-Oltra S, Carda M, Murga J, Falomir E, Marco J A (2008). Chem – Eur J.

[44] Weintraub P M, King C-H R (1997). J Org Chem.

[45] Handerson S, Schlaf M (2002). Org Lett.

[46] Dechert-Schmitt A-M, Cabral S, Kung D W (2016). Synlett.

[R47] 47A very clean carbamate product is required to obtain a good conversion into oxazolidinone.

[R48] 48A partial loss of product can occur during evaporation under reduced pressure due to its low boiling point.

