# Supplementary material for: Stereodivergent approach in the protected glycal synthesis of L-vancosamine, L-saccharosamine, L-daunosamine and L-ristosamine involving a ring-closing metathesis step
Source: Beilstein J Org Chem. 2018 Nov 29;14:2949–55. doi: 10.3762/bjoc.14.274 (PMC6278755; doi:10.3762/bjoc.14.274)
Supplement: File 1 — Experimental part and NMR spectra of all compounds. [file Beilstein_J_Org_Chem-14-2949-s001.pdf]

BEILSTEIN JOURNAL OF ORGANIC CHEMISTRY

Supporting Information

for

Stereodivergent approach in the protected glycal synthesis of L-vancosamine, L-saccharosamine, L-daunosamine and L-ristosamine involving a ring-closing metathesis step

Pierre-Antoine Nocquet, Aurélie Macé, Frédéric Legros, Jacques Lebreton, Gilles Dujardin, Sylvain Collet, Arnaud Martel, Bertrand Carboni and François Carreaux

Beilstein J. Org. Chem. **2018,** *14,* 2949–2955. doi:10.3762/bjoc.14.274

Experimental part and NMR spectra of all compounds

License and Terms: This is a supporting information file under the terms of the Creative Commons Attribution License (http://creativecommons.org/licenses/by/4.0). Please note that the reuse, redistribution and reproduction in particular requires that the authors and source are credited.
The license is subject to the *Beilstein Journal of Organic Chemistry* terms and conditions: (https://www.beilstein-journals.org/bjoc)

Table of contents

General information	S2
Synthesis of **5**	S3
General procedure for the aldol reaction (**7a**,**b**)	S4
Synthesis of **18**	S5
General procedure for the synthesis of silyl ethers (**8a**,**b**, **19**)	S6
General procedure for the reductive removal of the auxiliary group (**9a**,**b**,**10a**)	S8
General procedure for the Swern oxidation (**11a**,**b**)	S10
General procedure for the Wittig reaction (**13a**,**b**)	S11
Diastereoselective allylboration (**12b**)	S12
General procedure for the deprotection of PMB ether (**14a**,**b, 20**)	S13
General procedure for vinyl ether preparation (**15a**,**b, 21**)	S15
General procedure for dihydropyran preparation by ring metathesis (**16a**,**b, 22**)	S17
Synthesis of **23** and **26**	S19
General procedure for carbamate preparation (**17a**,**b**, **24**, **27**)	S20
General procedure for 2-oxazolidinone preparation (**1**,**3**)	S22
References	S23
^{1}H NMR and ^{13}C NMR spectra of all compounds	S24–86

General information

General experimental methods. Tetrahydrofuran (THF) and diethyl ether (Et_2O) were distilled over sodium/benzophenone. Toluene and cyclohexane were distilled over sodium and dichloromethane (DCM) was distilled over P_2O_5. DMSO was distilled over CaH_2 and stored over MS 4 Å. Triethylamine (Et_3N) was distilled over potassium hydroxide (KOH). All other commercially available chemicals were used without further purification. Reactions run at room temperature were performed between 20 and 25 °C. Solvent evaporations were conducted under reduced pressure at temperatures less than 45 °C. TLC was performed on silica gel plates visualized either with a UV lamp (254 nm) or using a staining solution ($KMnO_4$) followed by heating. Column chromatography was carried out under positive pressure using silica gel (0.006–0.200 mm, 60 Å) and the indicated solvents (v/v) unless otherwise precised. NMR spectra were recorded at 300 or 400 MHz for ^{1}H and 75 or 101 MHz for ^{13}C. Chemical shifts are given in ppm (δ) comparatively to solvent signal, which was used as an internal reference. Coupling constants (*J*) are given in Hertz (Hz), and the following abbreviations are used to describe the signal multiplicity: s (singlet), br s (broad singlet), d (doublet), t (triplet), q (quadruplet), and m (multiplet). Assignments were done with the aid of DEPT 135, COSY, and HMQC experiments. High-resolution mass spectra (HRMS) were recorded on a Micro-Tof-Q II or on a Q-Tof 2 using positive ion electrospray. Optical rotations were measured using 10 cm cell at 20 °C (sodium D line: 589 nm), and the concentration is expressed in g/dL. Melting points were measured without correction on a digital melting point apparatus.

(*S*)-2-((4-Methoxybenzyl)oxy)propanal (5)[1]

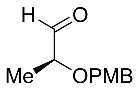

A solution of PMBOH (109 mmol) in Et_2O (44 mL) was added to a solution of NaH (60% in oil, 32.6 mmol, 0.3 equiv) in Et_2O (65 mL). The solution was stirred at room temperature for 45 min. After cooling to 0 °C, Cl_3CCN (109 mmol, 1 equiv) was added and the solution was stirred at 0 °C for 1 h, then at room temperature for 4 h. The solution was filtered through a pad of celite and a saturated aqueous solution of $NaHCO_3$ (40 mL) was added. The product was extracted with Et_2O (3 × 30 mL). The combined organic layer was dried over $MgSO_4$, filtered and concentrated under reduced pressure to give the desired imidate (28.5 g, 93%) as an orange oil which was used without further purification.

TfOH (0.202 mmol, 0.003 equiv) was added dropwise to a solution of imidate (101 mmol, 1.5 equiv) and methyl (L)-lactate (67.2 mmol) in a mixture CH_2Cl_2/cyclohexane (6:4, 152 mL) cooled at 0 °C. The solution was stirred at 0 °C for 15 min, then at room temperature for 21 h. The solution was filtered through a pad of celite and concentrated under reduced pressure. A saturated aqueous solution of $NaHCO_3$ (30 mL) was added and the product was extracted with CH_2Cl_2 (3 × 30 mL). The combined organic layer was dried over $MgSO_4$, filtered and concentrated under reduced pressure. The crude product was purified by flash chromatography (EtOAc/cyclohexane, 5:95 to 10:90) to afford ester (14.7 g, 92%) as a yellow oil.

DIBAL-H (1 M in CH_2Cl_2, 46.3 mmol, 1.05 equiv) was added with a syringe pump (40 mL/h) to a solution of ester (10.5 g, 44.1 mmol) in CH_2Cl_2 (110 mL) cooled at –78 °C. At the end of the addition, the solution was stirred at –78 °C for 1.5 h. A saturated aqueous solution of Rochelle salt (40 mL) was then added slowly and the solution was allowed to warm up to rt overnight. Water (20 mL) was added and the product was extracted with CH_2Cl_2 (3× 30 mL). The combined organic layer was dried over $MgSO_4$, filtered and concentrated under reduced pressure. The crude product was purified by flash chromatography (EtOAc/cyclohexane, 1:5 to 1:3) to afford aldehyde **5** (5.48 g, 64%) as a yellow oil.

R_f = 0.30 (EtOAc/cyclohexane = 1:3)
$[\alpha]_D^{20}$ = –49.5 (c 1.1, $CHCl_3$); Lit. $[\alpha]_D^{23}$ = –40.87 (c 1.03, $CHCl_3$)
^{1}H NMR (300 MHz, $CDCl_3$) δ 9.64 (d, *J* = 1.9 Hz, 1H), 7.31 – 7.26 (m, 2H, 2), 6.92 – 6.87 (m, 2H), 4.58 (d, *J* = 11.5 Hz, 1H), 4.53 (d, *J* = 11.5 Hz, 1H), 3.87 (qd, *J* = 6.9, 1.9 Hz, 1H), 3.81 (s, 3H), 1.31 (d, *J* = 6.9 Hz, 3H);
^{13}C NMR (75 MHz, $CDCl_3$) δ 203.6, 159.6, 129.7, 129.4, 114.0, 79.2, 71.8, 55.3, 15.4;
Spectroscopic data are in accordance with those reported in literature.

General procedure for the aldol reaction (7a,b).

Bu_2BOTf (1M in CH_2Cl_2, 3.8 mL, 3.81 mmol, 2 equiv) followed by Et_3N (0.80 mL, 5.71 mmol, 3 equiv) were added dropwise to a solution of (*S*) or (*R*)-4-benzyl-3-propionyloxazolidin-2-one (444 mg, 1.90 mmol, 1 equiv) in CH_2Cl_2 (4 mL) cooled at 0 °C. The solution was stirred at 0 °C for 1 h. After cooling to –78 °C, a solution of aldehyde **5** (740 mg, 3.81 mmol, 2 equiv) in CH_2Cl_2 (1.6 mL) was added dropwise. The solution was stirred at –78 °C for 2 h, then allowed to warm up slowly to room temperature overnight. MeOH (0.68 mL) followed by 35% H_2O_2 (2.0 mL) were added dropwise and the solution was stirred at room temperature for 1 h. Water was added and the product was extracted with CH_2Cl_2 (3 × 5 mL). The combined organic layer was dried over $MgSO_4$, filtered and concentrated under reduced pressure. The crude product was purified by flash chromatography to afford alcohol **7**.

(*S*)-4-Benzyl-3-((2*S*,3*S*,4*S*)-3-hydroxy-4-((4-methoxybenzyl)oxy)-2-methylpentanoyl)oxazolidin-2-one (7a)[1]

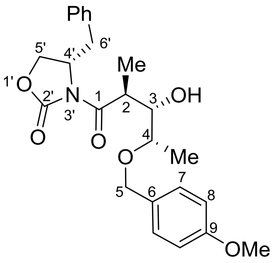

Prepared from (*S*)-4-benzyl-3-propionyloxazolidin-2-one and purified using a cyclohexane/ethyl acetate mixture (5:1 to 2:1) as eluent. The product **7a** was isolated as a viscous colorless oil (731 mg) in 90% yield.

R_f = 0.22 (EtOAc/cyclohexane = 1:5)
$[\alpha]_D^{20}$ = +71.9 (c 1.0, $CHCl_3$); Lit. $[\alpha]_D^{27.8}$ = +32.5 (c 1.85, $CHCl_3$);
^1H NMR (300 MHz, $CDCl_3$) δ 7.38 – 7.14 (m, 7H), 6.92 – 6.81 (m, 2H), 4.59 (d, *J* = 11.4 Hz, 1H), 4.45 – 4.41 (m, 1H), 4.30 (d, *J* = 11.4 Hz, 1H), 4.09 (dd, *J* = 9.0, 2.6 Hz, 1H), 4.03 – 3.99 (m, 1H), 3.89 – 3.85 (m, 1H), 3.81 – 3.74 (m, 4H), 3.51 (qd, *J* = 6.2, 4.1 Hz, 1H), 3.23 (dd, *J* = 13.3, 3.3 Hz, 1H), 2.70 (dd, *J* = 13.3, 9.6 Hz, 1H), 2.39 (d, *J* = 6.8 Hz, 1H), 1.31 – 1.24 (m, 6H);
^{13}C NMR (75 MHz, $CDCl_3$) δ 175.4, 159.2, 152.9, 135.3, 130.3, 129.7, 129.4, 128.9, 127.4, 113.8, 75.2, 74.2, 70.1, 66.0, 55.4, 55.3, 40.5, 37.8, 15.5, 12.8;
HRMS calcd for $C_{24}H_{29}NO_6Na$ $(M+Na)^+$: 450.1887 Found: 450.1888.

(*R*)-4-Benzyl-3-((2*R*,3*R*,4*S*)-3-hydroxy-4-((4-methoxybenzyl)oxy)-2-methylpentanoyl)oxazolidin-2-one (7b)

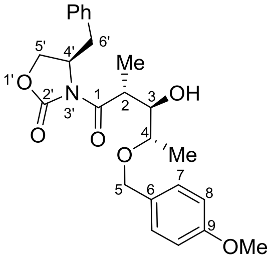

Prepared from (*R*)-4-benzyl-3-propionyloxazolidin-2-one and purified using a cyclohexane/ethyl acetate mixture (3:1 to 2:1) as eluent. The product **7b** was isolated as a viscous colorless oil (779 mg) in 96% yield.

R_f = 0.26 (EtOAc/cyclohexane = 1:2);
$[\alpha]_D^{20}$ = –21.5 (c 1.0, $CHCl_3$);
^1H NMR (300 MHz, $CDCl_3$) δ 7.37 – 7.24 (m, 5H), 7.22 – 7.15 (m, 2H), 6.92 – 6.85 (m, 2H), 4.65 – 4.50 (m, 2H), 4.34 (d, *J* = 11.1 Hz, 1H), 4.16 – 4.04 (m, 2H), 4.02 – 3.86 (m, 2H), 3.78 (s, 3H), 3.50 – 3.46 (m, 1H), 3.21 (dd, *J* = 13.4, 3.4 Hz, 1H), 2.90 (d, *J* = 3.6 Hz, 1H), 2.75 (dd, *J* = 13.4, 9.4 Hz, 1H), 1.30 (d, *J* = 6.1 Hz, 3H), 1.22 (d, *J* = 7.1 Hz, 3H);
^{13}C NMR (75 MHz, $CDCl_3$) δ 177.5, 159.2, 152.8, 135.1, 130.4, 129.4, 129.0, 127.4, 113.8, 74.8, 74.5, 70.4, 66.0, 55.3, 55.0, 39.2, 37.8, 16.0, 11.9;
HRMS calcd for $C_{24}H_{29}NO_6Na$ $(M+Na)^+$: 450.1887 Found: 450.1888.

(2*S*,3*S*)-2-((4-Methoxybenzyl)oxy)hex-5-en-3-ol (18)[2]

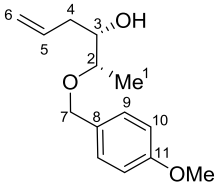

$MgBr_2{\bullet}Et_2O$ (6.97 g, 27.0 mmol, 1.05 equiv) was added portionwise to a solution of aldehyde **5** (4.99 g, 25.7 mmol, 1 equiv) in CH_2Cl_2 (165 mL) cooled at –78 °C. The solution was stirred at –78 °C for 30 min. AllylMgBr (1M in Et_2O, 51 mL, 51.4 mmol, 2 equiv) was added with a syringe pump (30 mL/h) and the solution was then allowed to warm up to room temperature overnight. 1M HCl (50 mL) was added and the product was extracted with CH_2Cl_2 (3×30 mL). The combined organic layer was dried over $MgSO_4$, filtered and concentrated under reduced pressure. The crude product was purified by flash chromatography (EtOAc/cyclohexane, 1:4 to 1:3) to afford alcohol **18** (5.17 g, 85%) as a slightly yellow oil.

R_f = 0.36 (EtOAc/cyclohexane = 1:3);
dr: 93:7

$[\alpha]_D^{20}$ = +43.0 (c 1.0, $CHCl_3$); Lit. $[\alpha]_D^{26}$ = +48.6 (c 1.0, CH_2Cl_2);
^{1}H NMR (300 MHz, $CDCl_3$) δ 7.30 – 7.23 (m, 2H), 6.91 – 6.85 (m, 2H), 5.96 – 5.90 (m, 1H), 5.14 – 5.05 (m, 2H), 4.60 (d, *J* = 11.1 Hz, 1H), 4.37 (d, *J* = 11.1 Hz, 1H), 3.81 (s, 3H), 3.56 – 3.37 (m, 2H), 2.55 (d, *J* = 3.6 Hz, 1H), 2.37 – 2.33 (m, 1H), 2.24 – 2.20 (m, 1H), 1.19 (d, *J* = 6.1 Hz, 3H);
^{13}C NMR (75 MHz, $CDCl_3$) δ 159.3, 134.8, 130.4, 129.5, 117.2, 113.9, 77.3, 74.3, 70.7, 55.3, 37.5, 15.5;
HRMS calcd for $C_{14}H_{20}O_3Na$ ($M+Na^+$): 259.1304 Found: 259.1305.
Spectroscopic data are in accordance with those reported in literature.

General procedure for the synthesis of silyl ethers (8a,b,19).

2,6-Lutidine (4.68 mmol, 2 equiv) followed by TBSOTf (3.51 mmol, 1.5 equiv) were added to a solution of **7** or **18** (2.34 mmol, 1 equiv) in CH_2Cl_2 (6.8 mL) cooled at 0 °C. The solution was allowed to warm up to room temperature and stirred for 22 h. A saturated aqueous solution of NH_4Cl (4 mL) was added and the product was extracted with CH_2Cl_2 (3 × 8 mL). The combined organic layer was dried over $MgSO_4$, filtered and concentrated under reduced pressure. The crude product was purified by flash chromatography to afford the silylated compound.

(*S*)-4-Benzyl-3-((2*S*,3*S*,4*S*)-3-((*tert*-butyldimethylsilyl)oxy)-4-((4-methoxybenzyl)oxy)-2-methylpentanoyl)oxazolidin-2-one (8a)

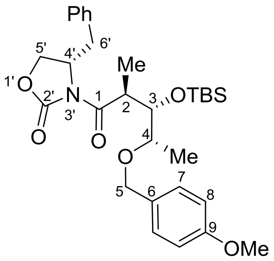

Prepared from **7a** and purified using a cyclohexane/ethyl acetate mixture (5:1) as eluent. The product **8a** was isolated as a viscous yellow oil (1.1 g) in 85% yield.

R_f = 0.47 (EtOAc/cyclohexane = 1:5);
$[\alpha]_D^{20}$ = +33.5 (c 1.3, $CHCl_3$);
^{1}H NMR (300 MHz, $CDCl_3$) δ 7.35 – 7.19 (m, 5H), 7.13 – 7.05 (m, 2H), 6.82 – 6.74 (m, 2H), 4.38 – 4.29 (m, 3H), 4.13 (dq, *J* = 9.5, 6.7 Hz, 1H), 3.92 (dddd, *J* = 9.9, 7.8, 3.4, 2.4 Hz, 1H), 3.78 (dd, *J* = 8.8, 2.4 Hz, 1H), 3.70 - 3.61 (m, 4H), 3.38 (dd, *J* = 8.8, 8.8 Hz, 1H), 3.14 (dd, *J* = 13.4, 3.4 Hz, 1H), 2.59 (dd, *J* = 13.4, 9.9 Hz, 1H), 1.22 (s, 3H), 1.19 (s, 3H), 0.91 (s, 9H), 0.14 (s, 3H), 0.09 (s, 3H);
^{13}C NMR (75 MHz, $CDCl_3$) δ 175.9, 159.1, 153.4, 135.8, 130.9, 129.4, 129.3, 128.8, 127.1, 113.5, 78.0, 73.4, 71.1, 65.7, 55.4, 55.2, 38.0, 36.8, 25.9, 18.1, 15.6, 13.4, –4.4, –4.6;
HRMS calcd for $C_{30}H_{43}NO_6SiNa$ $(M+Na)^+$: 564.2752 Found: 564.2753.

(*R*)-4-Benzyl-3-((2*R*,3*R*,4*S*)-3-((*tert*-butyldimethylsilyl)oxy)-4-((4-methoxybenzyl)oxy)-2-methylpentanoyl)oxazolidin-2-one (8b)

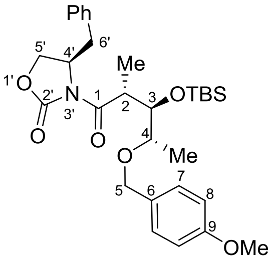

Prepared from **7b** and purified using a cyclohexane/ethyl acetate mixture (5:1) as eluent. The product **8b** was isolated as a viscous colorless oil (1.0 g) in 79% yield.

R_f = 0.34 (EtOAc/cyclohexane = 1:5);
$[\alpha]_D^{20}$ = –40.7 (c 1.0, $CHCl_3$);
^{1}H NMR (300 MHz, $CDCl_3$) δ 7.37 – 7.23 (m, 5H), 7.21 – 7.14 (m, 2H), 6.87 – 6.80 (m, 2H), 4.52 (d, *J* = 11.3 Hz, 1H), 4.40 – 4.28 (m, 2H), 4.15 (dd, *J* = 8.6, 4.8 Hz, 1H), 4.01 – 3.90 (m, 2H), 3.75 (s, 3H), 3.55 (dd, *J* = 8.4, 8.4 Hz, 1H), 3.39 – 3.33 (m, 1H), 3.19 (dd, *J* = 13.3, 3.3 Hz, 1H), 2.68 (dd, *J* = 13.3, 9.5 Hz, 1H), 1.26 (d, *J* = 7.0 Hz, 3H), 1.22 (d, *J* = 6.2 Hz, 3H), 0.94 (s, 9H), 0.15 (s, 3H), 0.13 (s, 3H);
^{13}C NMR (75 MHz, $CDCl_3$) δ 175.8, 158.9, 153.0, 135.4, 130.9, 129.4, 128.9, 128.8, 127.3, 113.5, 78.7, 75.9, 70.3, 65.6, 55.3, 55.2, 41.7, 37.8, 26.1, 18.4, 16.0, 15.3, –3.7, –4.0;
HRMS calcd for $C_{30}H_{43}NO_6SiNa$ $(M+Na)^+$: 564.2752 Found: 564.2752.

tert-Butyl(((2_S_,3_S_)-2-((4-methoxybenzyl)oxy)-hex-5-en-3-yl)oxy)dimethylsilane (19)[2]

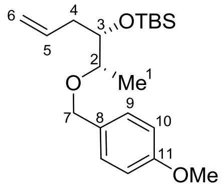

Prepared from **18** and purified using a cyclohexane/ethyl acetate mixture (95:5) as eluent. The product **19** was isolated as a colorless oil (688 mg) in 84% yield.

R_f = 0.57 (EtOAc/cyclohexane = 1:9);
$[\alpha]_D^{20}$ = +4.5 (c 1.0, $CHCl_3$); lit. $[\alpha]_D^{20}$ = +3.2 (c 2.28, CH_2Cl_2)
^{1}H NMR (300 MHz, $CDCl_3$) δ 7.30 – 7.23 (m, 2H), 6.91 – 6.85 (m, 2H), 5.82 –5.84 (m, 1H), 5.10 – 4.97 (m, 2H), 4.52 (d, J = 11.6 Hz, 1H), 4.44 (d, J = 11.6 Hz, 1H), 3.80 (s, 3H), 3.75 – 3.70 (m, 1H), 3.50 – 3.45 (m, 1H), 2.40 – 2.36 (m, 1H), 2.15 – 2.10 (m, 1H), 1.12 (d, J = 6.4 Hz, 3H), 0.87 (s, 9H), 0.02 (s, 3H), –0.01 (s, 3H);
^{13}C NMR (75 MHz, $CDCl_3$) δ 159.1, 136.3, 131.2, 129.2, 116.5, 113.8, 77.1, 73.9, 70.8, 55.3, 36.3, 25.9, 18.1, 14.1, –4.4, –4.5;
HRMS calcd for $C_{20}H_{34}NO_6SiNa$ $(M+Na)^+$: 373.2169 Found: 373.2170.
Spectroscopic data are in accordance with those reported in literature.

General procedure for the reductive removal of the auxiliary group (9a,b, 10a).

H_2O (6.34 mmol, 3 equiv) followed by $LiBH_4$ (2M in THF, 8.45 mmol, 4 equiv) were added to a solution of **8** (2.11 mmol, 1 equiv) in Et_2O (15 mL) cooled at 0 °C. The solution was stirred at 0 °C for 10 min then at room temperature for 3 h. 1 M aqueous NaOH (5 mL) was added and the solution was stirred at room temperature for 10 min. The product was extracted with Et_2O (3 × 8 mL). The combined organic layer was dried over $MgSO_4$, filtered and concentrated under reduced pressure. The crude product was purified by flash chromatography to afford alcohol **9** and **10**.

(2*R*,3*S*,4*S*)-3-((*tert*-Butyldimethylsilyl)oxy)-4-((4-methoxybenzyl)oxy)-2-methylpentan-1-ol (9a)

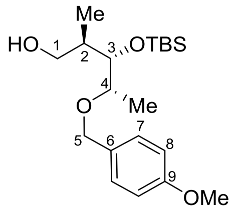

Prepared from **8a** and purified using a cyclohexane/ethyl acetate mixture (2:1) as eluent. The product **9a** was isolated as a slightly yellow oil (513 mg) in 66% yield and amide **10a** as a viscous colorless oil (245 mg) in 23% yield.

R_f = 0.48 (EtOAc/cyclohexane = 1:2)
$[\alpha]_D^{20}$ = +1.9 (c 1.0, $CHCl_3$);
^1H NMR (300 MHz, $CDCl_3$) δ 7.28 – 7.22 (m, 2H), 6.91 – 6.85 (m, 2H), 4.58 (d, *J* = 11.4 Hz, 1H), 4.43 (d, *J* = 11.4 Hz, 1H), 3.80 (s, 3H), 3.66 – 3.54 (m, 3H), 3.50 – 3.45 (m, 1H), 3.14 – 3.10 (m, 1H), 1.93 – 1.86 (m, 1H), 1.22 (d, *J* = 6.1 Hz, 3H), 0.90 (d, *J* = 6.9 Hz, 3H), 0.87 (s, 9H), 0.02 (s, 3H), –0.04 (s, 3H);
^{13}C NMR (75 MHz, $CDCl_3$) δ 159.4, 130.1, 129.4, 113.9, 77.3, 76.2, 70.8, 65.9, 55.3, 38.1, 25.9, 18.2, 14.4, 14.1, –4.4, –4.6.
HRMS calcd for $C_{20}H_{36}O_4SiNa$ $(M+Na)^+$: 391.2275 Found: 391.2275.

(2*S*,3*S*,4*S*)-3-((*tert*-Butyldimethylsilyl)oxy)-*N*-((*S*)-1-hydroxy-3-phenylpropan-2-yl)-4-((4-methoxybenzyl)oxy)-2-methylpentanamide (10a)

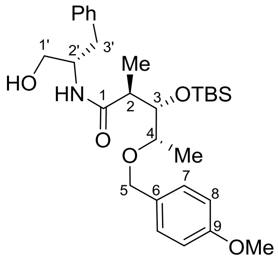

R_f = 0.22 (EtOAc/cyclohexane = 1:2)
$[\alpha]_D^{20}$ = –4.2 (c 1.0, $CHCl_3$);
^1H NMR (300 MHz, $CDCl_3$) δ 7.31 – 7.15 (m, 7H), 6.88 – 6.82 (m, 2H), 6.03 (d, *J* = 8.0 Hz, 1H), 4.44 (d, *J* = 11.9 Hz, 1H), 4.40 (d, *J* = 11.9 Hz, 1H), 4.13 – 4.08 (m, 1H), 3.84 (dd, *J* = 7.1, 3.3 Hz, 1H), 3.78 (s, 3H), 3.62 – 3.53 (m, 2H), 3.25 – 3.13 (m, 2H), 2.78 (dd, *J* = 13.7, 7.9 Hz, 1H), 2.73 (dd, *J* = 13.7, 7.4 Hz, 1H), 2.45 – 2.40 (m, 1H), 1.14 (d, *J* = 6.4 Hz, 3H), 1.05 (d, *J* = 7.0 Hz, 3H), 0.90 (s, 9H), –0.08 (s, 3H), 0.03 (s, 3H);
^{13}C NMR (75 MHz, $CDCl_3$) δ 174.3, 159.4, 138.0, 130.1, 129.7, 129.3, 128.5, 126.5, 113.8, 75.4, 74.9, 70.9, 62.3, 55.3, 52.3, 42.7, 37.4, 25.9, 18.1, 15.3, 14.5, –4.4, –4.6;
HRMS calcd for $C_{29}H_{45}NO_5SiNa$ $(M+Na)^+$: 538.2953 Found: 538.2955.

(2*S*,3*R*,4*S*)-3-((*tert*-Butyldimethylsilyl)oxy)-4-((4-methoxybenzyl)oxy)-2-methylpentan-1-ol (9b).

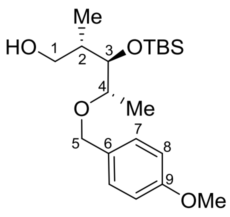

Prepared from **8b** and purified using a cyclohexane/ethyl acetate mixture (3:1) as eluent. The product **9b** was isolated as a slightly yellow oil (466 mg) in 60% yield.

R_f = 0.32 (EtOAc/cyclohexane = 1:3)
$[\alpha]_D^{20}$ = +15.0 (c 1.0, $CHCl_3$);
^{1}H NMR (300 MHz, $CDCl_3$) δ 7.31 – 7.24 (m, 2H), 6.92 – 6.86 (m, 2H), 4.54 (d, *J* = 11.3 Hz, 1H), 4.44 (d, *J* = 11.3 Hz, 1H), 3.82 (s, 3H), 3.78 (dd, *J* = 4.9, 3.3 Hz, 1H), 3.63 – 3.49 (m, 3H), 2.10 – 1.96 (m, 2H), 1.23 (d, *J* = 6.3 Hz, 3H), 0.93 (s, 9H), 0.91 (d, *J* = 7.4 Hz, 3H), 0.11 (s, 3H), 0.10 (s, 3H);
^{13}C NMR (75 MHz, $CDCl_3$) δ 159.1, 130.8, 129.3, 113.8, 76.6, 76.5, 70.6, 65.9, 55.3, 39.2, 26.1, 18.3, 16.4, 12.4, –3.9, –4.5;
HRMS calcd for $C_{20}H_{36}NO_4Si$ $(M+Na)^+$: 391.2275 Found: 391.2275.

General procedure for the Swern oxidation (11a,b).

Oxalyl chloride (2.99 mmol, 2 equiv) was added dropwise to a solution of DMSO (5.98 mmol, 4 equiv) in CH_2Cl_2 (10 mL) cooled at –78 °C. The solution was stirred at –78 °C for 30 min. A solution of **9** (1.50 mmol, 1 equiv) in CH_2Cl_2 (4 mL) was then added dropwise and the solution was stirred at – 78 °C for 1 h. Et_3N (6.88 mmol, 4.6 equiv) was added dropwise and the solution was allowed to warm up to –20 °C over 2 h. Water (50 mL) was then added. The product was extracted with CH_2Cl_2 (3 × 20 mL). The combined organic layer was dried over $MgSO_4$, filtered and concentrated under reduced pressure. The crude product was purified by flash chromatography to afford the aldehyde **11**.

(2*S*,3*S*,4*S*)-3-((*tert*-Butyldimethylsilyl)oxy)-4-((4-methoxybenzyl)oxy)-2-methylpentanal (11a)

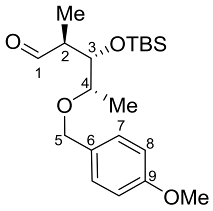

Prepared from **9a** and purified using a cyclohexane/ethyl acetate mixture (4:1) as eluent. The product **11a** was isolated as a slightly yellow oil (530 mg) in 96% yield.

R_f = 0.56 (EtOAc/cyclohexane = 1:3);
$[\alpha]_D^{20}$ = +22.1 (c 1.0, $CHCl_3$);
^{1}H NMR (300 MHz, $CDCl_3$) δ 9.72 (s, 1H), 7.24 – 7.18 (m, 2H), 6.90 – 6.84 (m, 2H), 4.46 (d, *J* = 11.5 Hz, 1H), 4.34 (d, *J* = 11.5 Hz, 1H), 4.01 – 3.95 (m, 1H), 3.80 (s, 3H), 3.57 – 3.53 (m, 1H), 2.58 – 2.53 (m, 1H), 1.17 (d, *J* = 6.4 Hz, 3H), 1.05 (d, *J* = 6.9 Hz, 3H), 0.88 (s, 9H), 0.05 (s, 3H), 0.03 (s, 3H);
^{13}C NMR (75 MHz, $CDCl_3$) δ 203.3, 159.2, 130.4, 129.4, 113.8, 75.4, 74.7, 70.4, 55.3, 49.0, 25.9, 18.2, 14.7, 10.3, –4.4, –4.7;
HRMS calcd for $C_{20}H_{34}O_4SiNa$ $(M+Na)^+$: 389.2118 Found: 389.2119.

(2*R*,3*R*,4*S*)-3-((*tert*-Butyldimethylsilyl)oxy)-4-((4-methoxybenzyl)oxy)-2-methylpentanal (11b)

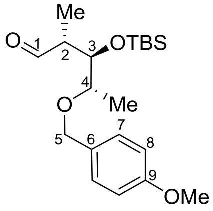

Prepared from **9b** and purified using a cyclohexane/ethyl acetate mixture (4:1) as eluent. The product **11b** was isolated as a slightly yellow oil (400 mg) in 73% yield.

R_f = 0.62 (EtOAc/cyclohexane = 1:3);
$[\alpha]_D^{20}$ = –1.8 (c 1.0, $CHCl_3$);
^{1}H NMR (400 MHz, $CDCl_3$) δ 9.69 (d, *J* = 1.0 Hz, 1H), 7.25 – 7.20 (m, 2H), 6.89 – 6.84 (m, 2H), 4.53 (d, *J* = 11.2 Hz, 1H), 4.36 (d, *J* = 11.2 Hz, 1H), 4.07 (dd, *J* = 6.4, 3.5 Hz, 1H), 3.80 (s, 3H), 3.47 – 3.43 (m, 1H), 2.75 – 2.70 (m, 1H), 1.23 (d, *J* = 6.2 Hz, 3H), 1.05 (d, *J* = 6.9 Hz, 3H), 0.87 (s, 9H), 0.08 (s, 3H), –0.01 (s, 3H);
^{13}C NMR (126 MHz, $CDCl_3$) δ 204.7, 159.2, 130.4, 129.5, 113.8, 76.0, 74.6, 70.6, 55.3, 50.0, 26.0, 18.2, 16.3, 8.0, –4.0, –4.1;
HRMS calcd for $C_{20}H_{34}NO_4SiNa$ $(M+Na)^+$: 389.2118 Found: 389.2119.

General procedure for the Wittig reaction (13a,b).

BuLi (2.3 M in hexane, 9.10 mmol, 2.5 equiv) was added dropwise to a solution of methyltriphenylphosphonium bromide (10.9 mmol, 3 equiv) in THF (15 mL) cooled at 0 °C. The solution was warmed up to room temperature and then cooled at –78 °C. A solution of **11** (3.64 mmol, 1 equiv) in THF (15 mL) was added and the solution was stirred at room temperature for 14 h. A saturated aqueous solution of NH_4Cl (10 mL) was added and the product was extracted with EtOAc (3 × 10 mL). The combined organic layer was dried over $MgSO_4$, filtered and concentrated under reduced pressure. The crude product was purified by flash chromatography to afford the alkene **13**.

***tert*-Butyl(((2*S*,3*S*,4*R*)-2-((4-methoxybenzyl)oxy)-4-methylhex-5-en-3-yl)oxy)dimethylsilane (13a)**

Prepared from **11a** and purified using a cyclohexane/ethyl acetate mixture (95:5) as eluent. The product **13a** was isolated as a slightly yellow oil (1.24 g) in 94% yield.

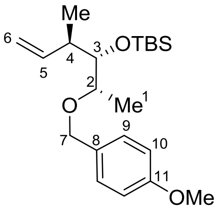

R_f = 0.75 (EtOAc/cyclohexane = 1:5);
$[\alpha]_D^{20}$ = +0.3 (c 1.0, $CHCl_3$);
^{1}H NMR (300 MHz, $CDCl_3$) δ 7.29 – 7.23 (m, 2H), 6.90 – 6.84 (m, 2H), 5.91 (ddd, *J* = 17.5, 10.3, 7.4 Hz, 1H), 5.02 – 4.92 (m, 2H), 4.49 (d, *J* = 11.6 Hz, 1H), 4.43 (d, *J* = 11.6 Hz, 1H), 3.81 (s, 3H), 3.58 – 3.54 (m, 1H), 3.50 – 3.45 (m, 1H), 2.44 – 2.41 (m, 1H), 1.15 (d, *J* = 6.3 Hz, 3H), 1.00 (d, *J* = 6.8 Hz, 3H), 0.89 (s, 9H), 0.02 (s, 3H), –0.01 (s, 3H);
^{13}C NMR (75 MHz, $CDCl_3$) δ 159.0, 143.2, 131.3, 129.1, 113.7, 113.0, 77.9, 77.6, 70.6, 55.3, 39.9, 26.1, 18.4, 15.4, 14.9, –4.0, –4.3;
HRMS calcd for $C_{21}H_{36}O_3SiNa$ $(M+Na)^+$: 387.2326 Found: 387.2327.

***tert*-Butyl(((2*S*,3*R*,4*S*)-2-((4-methoxybenzyl)oxy)-4-methylhex-5-en-3-yl)oxy)dimethylsilane (13b).**

Prepared from **11b** and purified using a cyclohexane/ethyl acetate mixture (98:2) as eluent. The product **13b** was isolated as a slightly yellow oil (1.13 g) in 86% yield.

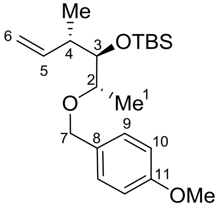

R_f = 0.25 (EtOAc/cyclohexane = 2:98);
$[\alpha]_D^{20}$ = +4.8 (c 1.0, $CHCl_3$);
^{1}H NMR (300 MHz, $CDCl_3$) δ 7.29 – 7.22 (m, 2H), 6.91 – 6.83 (m, 2H), 5.74 (ddd, *J* = 17.2, 10.3, 7.9 Hz, 1H), 5.03 – 4.92 (m, 2H), 4.46 (d, *J* = 11.4 Hz, 1H), 4.37 (d, *J* = 11.4 Hz, 1H), 3.80 (s, 3H), 3.58 (dd, *J* = 6.5, 3.7 Hz, 1H), 3.52 – 3.48 (m, 1H), 2.35 – 2.30 (m, 1H), 1.13 (d, *J* = 6.2 Hz, 3H), 1.02 (d, *J* = 6.8 Hz, 3H), 0.90 (s, 9H), 0.06 (s, 3H), 0.04 (s, 3H);
^{13}C NMR (75 MHz, $CDCl_3$) δ 159.0, 142.1, 131.2, 129.2, 114.0, 113.7, 78.4, 76.4, 70.3, 55.3, 41.8, 26.2, 18.5, 16.3, 14.3, –3.7, –4.3;
HRMS calcd for $C_{21}H_{36}O_3SiNa$ $(M+Na)^+$: 387.2326 Found: 387.2326.

Diastereoselective allylboration

(*Z*)-Crotylboronic acid pinacol ester (3.85 mmol, 1.1 equiv) was added to a solution of aldehyde **5** (3.50 mmol, 1 equiv). The solution was stirred at room temperature for 3.5 days. Water (3 mL) and CH_2Cl_2 (3 mL) were added and the product was extracted with CH_2Cl_2 (3 × 3 mL). The combined organic layer was dried over $MgSO_4$, filtered and concentrated under reduced pressure. The crude product was purified by flash chromatography (EtOAc/cyclohexane, 1:7 to 1:3) to afford alcohol **12b** (727 mg, 83%, dr 92:8) as a colorless oil.

(2*S*,3*R*,4*S*)-2-((4-Methoxybenzyl)oxy)-4-methylhex-5-en-3-ol (12b)

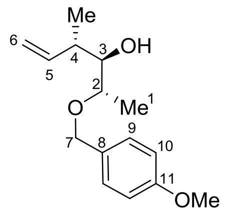

R_f = 0.41 (EtOAc/cyclohexane = 1:3);
$[\alpha]_D^{20}$ = +14.6 (c 1.0, $CHCl_3$);
^{1}H NMR (300 MHz, $CDCl_3$) δ 7.30 – 7.20 (m, 2H), 6.92 – 6.85 (m, 2H), 5.67 (ddd, *J* = 17.2, 10.3, 8.2 Hz, 1H), 5.10 – 4.98 (m, 2H), 4.51 (d, *J* = 11.2 Hz, 1H), 4.41 (d, *J* = 11.2 Hz, 1H), 3.80 (s, 3H), 3.60 – 3.49 (m, 2H), 2.33 – 2.28 (m, 1H), 2.22 – 2.18 (br s, 1H), 1.17 (d, *J* = 6.1 Hz, 3H), 1.09 (d, *J* = 6.7 Hz, 3H);
^{13}C NMR (75 MHz, $CDCl_3$) δ 159.2, 140.5, 130.6, 129.3, 115.1, 113.9, 75.8, 75.6, 70.2, 55.3, 40.3, 16.4, 13.0;
HRMS calcd for $C_{15}H_{22}O_3Na$ $(M+Na)^+$: 273.1461 Found: 273.1460.

General procedure for the deprotection of PMB ether (14a,b, 20).

DDQ (3.46 mmol, 1.5 equiv) was added to a solution of **13** or **19** (2.30 mmol, 1 equiv) in a solution of CH_2Cl_2 (15 mL) and pH 7 phosphate buffer (15 mL). The solution was stirred at room temperature for 4 h. A saturated aqueous solution of $NaHCO_3$ (5 mL) followed by a saturated aqueous solution of $Na_2S_2O_3$ (5 mL) were added. The product was extracted with CH_2Cl_2 (3×10 mL). The combined organic layer was dried over $MgSO_4$, filtered and concentrated under reduced pressure. The crude product was purified by flash chromatography to afford the alcohol.

(2*S*,3*S*,4*R*)-3-((*tert*-Butyldimethylsilyl)oxy)-4-methylhex-5-en-2-ol (14a)

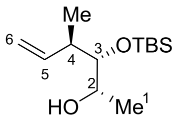

Prepared from **13a** and purified using a cyclohexane/ethyl acetate mixture (9:1) as eluent. The product **14a** was isolated as a colorless oil (474 mg) in 84% yield.

R_f = 0.44 (EtOAc/cyclohexane = 1:5);
$[\alpha]_D^{20}$ = +23.5 (c 1.1, $CHCl_3$);
^{1}H NMR (300 MHz, $CDCl_3$) δ 5.87 (ddd, J = 17.2, 10.4, 7.5 Hz, 1H), 5.07 – 5.03 (m, 1H), 5.01 – 4.98 (m, 1H), 3.78 – 3.74 (m, 1H), 3.36 (dd, J = 4.9, 4.0 Hz, 1H), 2.43 – 2.38 (m, 1H), 2.19 (d, J = 6.6 Hz, 1H), 1.15 (d, J = 6.4 Hz, 3H), 1.01 (d, J = 6.9 Hz, 3H), 0.93 (s, 9H), 0.10 (s, 6H);
^{13}C NMR (75 MHz, $CDCl_3$) δ 141.5, 114.5, 79.9, 68.0, 41.7, 26.1, 20.7, 18.4, 15.3, –3.7, –4.1;
HRMS calcd for $C_{13}H_{28}O_2SiNa$ $(M+Na)^+$: 267.1751 Found: 267.1751.

(2*S*,3*R*,4*S*)-3-((*tert*-Butyldimethylsilyl)oxy)-4-methylhex-5-en-2-ol (14b)[3]

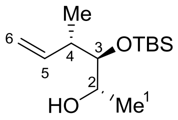

Prepared from **13b** and purified using a cyclohexane/ethyl acetate mixture (7:1) as eluent. The product **14b** was isolated as a colorless oil (524 mg) in 93% yield.

R_f = 0.37 (EtOAc/cyclohexane = 1:7);
$[\alpha]_D^{20}$ = –2.2 (c 1.0, $CHCl_3$); Lit. $[\alpha]_D^{20}$ = –2.76 (c 1.0, $CHCl_3$)
^{1}H NMR (300 MHz, $CDCl_3$) δ 5.77 (ddd, J = 17.3, 10.3, 7.8 Hz, 1H), 5.08 – 4.94 (m, 2H), 3.83 – 3.78 (m, 1H), 3.51 (dd, J = 6.1, 3.5 Hz, 1H), 2.35 –2.29 (m, 1H), 1.79 – 1.74 (br s, 1H), 1.14 (d, J = 6.4 Hz, 3H), 1.05 (d, J = 6.8 Hz, 3H), 0.92 (s, 9H), 0.09 (s, 3H), 0.08 (s, 3H);
^{13}C NMR (75 MHz, $CDCl_3$) δ 141.9, 114.0, 79.4, 69.9, 40.9, 26.1, 18.4, 17.5, 16.3, –3.9, -4.1;
HRMS calcd for $C_{13}H_{28}O_2SiNa$ $(M+Na)^+$: 267.1751 Found: 267.1752.
Spectroscopic data are in accordance with those reported in literature.

(2*S*,3*S*)-3-((*tert*-Butyldimethylsilyl)oxy)hex-5-en-2-ol (20).

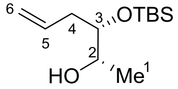

Prepared from **19** and purified using a cyclohexane/ethyl acetate mixture (9:1) as eluent. The product **20** was isolated as a colorless oil (482mg) in 92% yield.

R_f = 0.47 (EtOAc/cyclohexane = 1:5);
$[\alpha]_D^{20}$ = +35.2 (c 1.1, $CHCl_3$);
^{1}H NMR (300 MHz, $CDCl_3$) δ 5.81 (ddt, J = 17.3, 10.2, 7.2 Hz, 1H), 5.13 – 5.01 (m, 2H), 3.67 – 3.63 (m, 1H), 3.49 (dt, J = 6.6, 4.7 Hz, 1H), 2.42 – 2.38 (m, 1H), 2.47 – 2.43 (m, 1H), 2.23 – 2.17 (m, 1H), 1.13 (d, J = 6.3 Hz, 3H), 0.91 (s, 9H), 0.10 (s, 3H), 0.09 (s, 3H);
^{13}C NMR (75 MHz, $CDCl_3$) δ 134.3, 117.4, 76.2, 68.9, 38.5, 25.9, 19.5, 18.2, –4.1, –4.6;
HRMS calcd for $C_{12}H_{26}O_2SiNa$ $(M+Na)^+$: 253.1594 Found: 253.1595.

General procedure for vinyl ether preparation (15a,b, 21).

Et_3N (50.6 µmol, 7.5 mol %) was added to a solution of alcohol **14** or **20** (0.675 mmol, 1 equiv), $Pd(O_2CCF_3)_2$ (33.7 µmol, 5 mol %) and bathophenanthroline (33.7 µmol, 5 mol %) in *n*-butyl vinyl ether (8.7 mL). The solution was heated at 80 °C for 23 h. The solution was filtered through a pad of celite and concentrated under reduced pressure. The crude product was purified by flash chromatography to afford the corresponding vinyl ether.

tert-Butyldimethyl(((2*S*,3*S*,4*R*)-4-methyl-2-(vinyloxy)hex-5-en-3-yl)oxy)silane (15a).

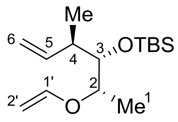

Prepared from **14a** and purified on deactivated silica gel using a cyclohexane/CH_2Cl_2 mixture (9:1) as eluent. The product **15a** was isolated as a colorless oil (145 mg) in 79% yield.

R_f = 0.51 (CH_2Cl_2/cyclohexane = 1:9);
$[\alpha]_D^{20}$ = –9.0 (c 1.0, $CHCl_3$);
^{1}H NMR (300 MHz, $CDCl_3$) δ 6.25 (dd, J = 14.2, 6.6 Hz, 1H), 5.87 (ddd, J = 17.4, 10.3, 7.3 Hz, 1H), 5.03– 4.99 (m, 1H), 4.97– 4.93 (m, 1H), 4.23 (dd, J = 14.2, 1.5 Hz, 1H), 3.98 (dd, J = 6.6, 1.5 Hz, 1H), 3.84– 3.80 (m, 1H), 3.56 (dd, J = 6.5, 3.7 Hz, 1H), 2.39– 2.33 (m, 1H), 1.17 (d, J = 6.4 Hz, 3H), 0.99 (d, J = 6.8 Hz, 3H), 0.88 (s, 9H), 0.05 (s, 3H), 0.04 (s, 3H);
^{13}C NMR (75 MHz, $CDCl_3$) δ 151.1, 142.7, 113.6, 88.2, 78.6, 78.2, 40.0, 26.2, 18.4, 15.9, 13.7, –3.9, –4.3;
HRMS calcd for $C_{15}H_{30}O_2SiNa$ $(M+Na)^+$: 293.1907 Found: 293.1908.

tert-Butyldimethyl(((2*S*,3*R*,4*S*)-4-methyl-2-(vinyloxy)hex-5-en-3-yl)oxy)silane (15b).

Prepared from **14b** and purified on deactivated silica gel using a cyclohexane/CH_2Cl_2 mixture (97:3) as eluent. The product **15b** was isolated as a colorless oil (97 mg) in 53% yield.

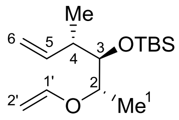

R_f = 0.53 (CH_2Cl_2/cyclohexane = 1:9);
$[\alpha]_D^{20}$ = –8.2 (c 1.0, $CHCl_3$);
^{1}H NMR (300 MHz, acetone-d6) δ 6.34 (dd, J = 14.2, 6.6 Hz, 1H), 5.81 (ddd, J = 17.2, 10.3, 8.5 Hz, 1H), 5.07 (ddd, J = 17.2, 2.0, 1.0 Hz, 1H), 5.00 (ddd, J = 10.3, 1.9, 0.7 Hz, 1H), 4.18 (dd, J = 14.2, 1.4 Hz, 1H), 4.01 (qd, J = 6.3, 2.5 Hz, 1H), 3.94 (dd, J = 6.7, 1.4 Hz, 1H), 3.68 (dd, J = 7.9, 2.5 Hz, 1H), 2.30– 2.25 (m, 1H), 1.15 (d, J = 6.4 Hz, 3H), 1.06 (d, J = 6.7 Hz, 3H), 0.93 (s, 9H), 0.13 (s, 3H), 0.09 (s, 3H);
^{13}C NMR (75 MHz, acetone-d6) δ 151.6, 142.2, 115.1, 88.2, 78.3, 77.8, 43.1, 26.6, 19.1, 17.7, 13.6, –3.6, –4.3;
HRMS calcd for $C_{15}H_{30}O_2SiNa$ $(M+Na)^+$: 293.1907 Found: 293.1907.

tert-Butyldimethyl(((2_S_,3_S_)-2-(vinyloxy)hex-5-en-3-yl)oxy)silane (21)

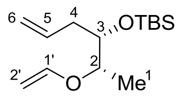

Prepared from **20** and purified on neutral aluminium oxide using cyclohexane as eluent. The product **21** was isolated as a slightly yellow oil (150 mg) in 80% yield.

R_f = 0.94 (EtOAc/cyclohexane = 4:96);
$[\alpha]_D^{20}$ = +9.4 (c 1.0, $CHCl_3$);
^{1}H NMR (300 MHz, $CDCl_3$) δ 6.29 (dd, J = 14.1, 6.6 Hz, 1H), 5.87–5.83 (m, 1H), 5.13 – 5.01 (m, 2H), 4.26 (dd, J = 14.1, 1.5 Hz, 1H), 3.97 (dd, J = 6.6, 1.5 Hz, 1H), 3.85– 3.80 (m, 1H), 3.74– 3.69 (m, 1H), 2.37– 2.30 (m, 1H), 2.17– 2.12 (m, 1H), 1.16 (d, J = 6.3 Hz, 3H), 0.89 (s, 9H), 0.06 (s, 6H);
^{13}C NMR (75 MHz, $CDCl_3$) δ 151.3, 135.2, 117.1, 88.0, 78.3, 73.8, 36.9, 25.9, 18.2, 14.7, –4.41, –4.44;
HRMS calcd for $C_{14}H_{28}O_2SiNa$ $(M+Na)^+$: 279.1751 Found: 279.1750.

General procedure for dihydropyran preparation by ring metathesis (16a,b, 22).

A solution of vinyl ether (1.04 mmol, 1 equiv) and Hoveyda–Grubbs catalyst 2nd Generation (41.5 µmol, 4 mol %) in degassed toluene (5 mL) was heated at 100 °C for 17 h. After cooling, the solution was concentrated under reduced pressure. The crude product was purified by flash chromatography (neutral aluminium oxide, CH_2Cl_2/cyclohexane) to afford the corresponding dihydropyran.

***tert*-Butyl(((2*S*,3*S*,4*R*)-2,4-dimethyl-3,4-dihydro-2*H*-pyran-3-yl)oxy)dimethylsilane (16a)**

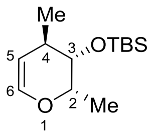

Prepared from **15a** and purified on deactivated silica gel using a cyclohexane/CH_2Cl_2 mixture (9:1) as eluent. The product **16a** was isolated as a colorless oil (191 mg) in 79% yield.

R_f = 0.26 (CH_2Cl_2/cyclohexane = 1:9);
$[\alpha]_D^{20}$ = –111.1 (c 1.0, $CHCl_3$);
^{1}H NMR (400 MHz, $CDCl_3$) δ 6.20 (dd, *J* = 6.0, 2.0 Hz, 1H), 4.49 (dd, *J* = 6.0, 3.0 Hz, 1H), 3.97 (qd, *J* = 6.5, 3.3 Hz, 1H), 3.45 (dd, *J* = 6.1, 3.3 Hz, 1H), 2.13 – 1.97 (m, 1H), 1.20 (d, *J* = 6.5 Hz, 3H), 1.00 (d, *J* = 7.0 Hz, 3H), 0.90 (s, 9H), 0.072 (s, 3H), 0.066 (s, 3H);
^{13}C NMR (101 MHz, $CDCl_3$) δ 140.8, 104.0, 73.2, 71.7, 31.9, 25.8, 19.8, 18.1, 14.1, –4.5, –4.6;
HRMS calcd for $C_{13}H_{26}O_2SiNa$ $(M+Na)^+$: 265.1594 Found: 265.1594.

***tert*-Butyl(((2*S*,3*R*,4*S*)-2,4-dimethyl-3,4-dihydro-2*H*-pyran-3-yl)oxy)dimethylsilane (16b).**

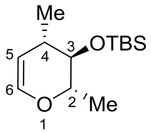

Prepared from **15b** and purified on deactivated silica gel using a cyclohexane/CH_2Cl_2 mixture (9:1) as eluent. The product **16b** was isolated as a colorless oil (208 mg) in 82% yield.

R_f = 0.44 (CH_2Cl_2/cyclohexane = 1:9);
$[\alpha]_D^{20}$ = +5.0 (c 1.1, $CHCl_3$);
^{1}H NMR (300 MHz, acetone-d6) δ 6.24 (dd, *J* = 5.9, 2.4 Hz, 1H), 4.45 (dd, *J* = 5.9, 2.0 Hz, 1H), 3.61 (qd, *J* = 8.9, 6.3 Hz, 1H), 3.16 (dd, *J* = 8.6, 8.6 Hz, 1H), 2.20 – 2.15 (m, 1H), 1.27 (d, *J* = 6.4 Hz, 3H), 1.04 (d, *J* = 7.0 Hz, 3H), 0.93 (s, 9H), 0.153 (s, 3H), 0.147 (s, 3H);
^{13}C NMR (75 MHz, acetone-d6) δ 142.9, 106.2, 77.9, 76.5, 37.2, 26.4, 19.9, 18.9, 18.7, –3.2, –3.4;
HRMS calcd for $C_{13}H_{26}O_2SiNa$ $(M+Na)^+$: 265.1594 Found: 265.1595.

tert-Butyldimethyl(((2S,3S)-2-methyl-3,4-dihydro-2H-pyran-3-yl)oxy)silane (22)[4]

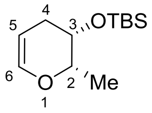

Prepared from **21** and purified on deactivated silica gel using a cyclohexane/CH_2Cl_2 mixture (9:1) as eluent. The product **22** was isolated as a colorless oil (157 mg) in 66% yield.

R_f = 0.26 (CH_2Cl_2/cyclohexane = 1:9);
$[\alpha]_D^{20}$ = –31.6 (c 1.0, $CHCl_3$); Lit. $[\alpha]_D^{20}$ = –33.6 (c 0.72, CH_2Cl_2);
^{1}H NMR (300 MHz, $CDCl_3$) δ 6.24 (dt, J = 6.1, 2.0 Hz, 1H), 4.58 –4.50 (m, 1H), 4.05 – 3.91 (m, 2H), 2.20 – 2.16 (m, 1H), 1.97 (dddd, J = 16.8, 6.7, 3.3, 2.1 Hz, 1H), 1.18 (d, J = 6.4 Hz, 3H), 0.89 (s, 9H), 0.07 (s, 6H);
^{13}C NMR (75 MHz, $CDCl_3$) δ 141.9, 97.1, 73.1, 66.5, 27.7, 25.8, 18.1, 13.9, –4.5, –4.8;
HRMS calcd for $C_{12}H_{24}O_2SiNa$ $(M+Na)^+$: 265.1594 Found: 265.1595.
Spectroscopic data are in accordance with those reported in literature.

(2*S*,3*S*)-2-Methyl-3,4-dihydro-2*H*-pyran-3-ol (23)

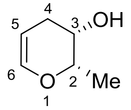

TBAF (1 M in THF, 0.420 mmol, 1.5 equiv) was added dropwise to a solution of **22** (0.280 mmol) in THF (4.2 mL) cooled at 0 °C. The solution was stirred at room temperature for 3 h. Saturated aqueous NH_4Cl (2 mL) were added and the product was extracted with Et_2O (3 × 5 mL). The combined organic layer was dried over $MgSO_4$, filtered and concentrated under reduced pressure (*P* > 200 mbar). The crude mixture was used directly in the next step.

^{1}H NMR (300 MHz, acetone-d6) *δ* 6.26 (dt, *J* = 6.3, 2.0 Hz, 1H), 4.56 – 4.52 (m, 1H), 3.98 – 3.93 (m, 1H), 3.87 – 3.83 (m, 1H), 3.61 (d, *J* = 6.3 Hz, 1H), 2.40 – 2.36 (m, 1H), 2.00 –1.95 (m, 1H), 1.21 (d, *J* = 6.5 Hz, 3H).

(2*S*,3*R*)-2-Methyl-3,4-dihydro-2*H*-pyran-3-ol (26)

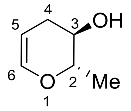

PPh_3 (0.690 mmol, 2 equiv) followed by 4-nitrobenzoic acid (0.518 mmol, 1.5 mmol) and DIAD (0.690 mmol, 2 equiv) were added to a solution of the alcohol **23** (0.345 mmol, 1 equiv) in THF (8.0 mL) cooled at 0 °C. The solution was stirred at room temperature for 16 h. Water (3 mL) was added and the product was extracted with Et_2O (3 × 4 mL). The combined organic layer was dried over $MgSO_4$, filtered and concentrated under reduced pressure. To the crude product in MeOH (0.85 mL) was added K_2CO_3 (72 mg). The solution was stirred at room temperature for 6 h. A saturated aqueous solution of NH_4Cl (1 mL) was added and the product was extracted with Et_2O (3 × 2 mL). The combined organic layer was dried over $MgSO_4$, filtered and concentrated under reduced pressure (*P* > 200 mbar). The crude product was purified by flash chromatography (Et_2O/pentane, 1:3 to 1:1) to afford the alcohol **26** (22 mg, 57%) as a colorless oil.

R_f = 0.23 (EtOAc/cyclohexane = 1:3);
$[\alpha]_D^{20}$ = +5.2 (c 1.0, $CHCl_3$);
^{1}H NMR (300 MHz, acetone-d6) *δ* 6.24 (dt, *J* = 6.0, 2.0 Hz, 1H), 4.58 (ddd, *J* = 5.8, 5.0, 2.5 Hz, 1H), 4.07 (d, *J* = 5.4 Hz, 1H), 3.65 – 3.44 (m, 2H), 2.27 – 2.22 (m, 1H), 1.96 – 1.92 (m, 1H), 1.27 (d, *J* = 6.2 Hz, 3H);
^{13}C NMR (300 MHz, acetone-d6) *δ* 143.8, 98.8, 76.4, 69.1, 30.3, 17.9.
HRMS calcd for $C_6H_{10}O_2Na$ $(M+Na)^+$: 137.0572 Found: 137.0572.

General procedure for carbamate preparation (17a,b, 24, 27):

TBAF (1 M in THF, 0.792 mmol, 2 equiv) was added dropwise to a solution of silylated alcohol **16a**,**b** or **22** (0.396 mmol) in THF (5.9 mL) cooled at 0 °C. The solution was stirred at room temperature for 16 h and then concentrated under reduced pressure (*P* > 200 mbar). The crude obtained (or purified **26**) was dissolved in CH_2Cl_2 (0.97 mL) and cooled at 0 °C. $Cl_3CCONCO$ (0.475 mmol, 1.2 equiv) was then added and the solution was stirred at 0 °C for 1 h then at room temperature for 4 h. The solution was then concentrated under reduced pressure. The crude was dissolved in MeOH (0.90 mL) and K_2CO_3 (39.6 µmol, 0.1 equiv) was added. The solution was stirred at room temperature for 17 h. A saturated aqueous solution of NH_4Cl (2 mL) was then added and the product was extracted with CH_2Cl_2 (3 × 2 mL). The combined organic layer was dried over $MgSO_4$, filtered and concentrated under reduced pressure. The crude product was purified by flash chromatography to afford the corresponding carbamate.

(2*S*,3*S*,4*R*)-2,4-Dimethyl-3,4-dihydro-2*H*-pyran-3-yl carbamate (17a)[5]

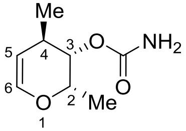

Prepared from **16a** and purified using a cyclohexane/ethyl acetate mixture (3:1) as eluent. The product **17a** was isolated as a white solid (53.6 mg) in 79% yield.

R_f = 0.17 (EtOAc/cyclohexane = 1:3);
mp: 100 – 105 °C;
$[\alpha]_D^{20}$ = –120.7 (c 1.0, $CHCl_3$); Lit. $[\alpha]_D^{22}$ = –84 (c 1.0, $CHCl_3$)
^{1}H NMR (300 MHz, $CDCl_3$) δ 6.37 (dd, *J* = 6.2, 1.5 Hz, 1H), 4.84 – 4.66 (br s, 2H), 4.68 (ddd, *J* = 6.0, 4.4, 1.5 Hz, 1H), 4.62 –4.57 (m, 1H), 3.99 (qd, *J* = 6.6, 1.6 Hz, 1H), 2.26 – 2.22 (m, 1H), 1.29 (d, *J* = 6.6 Hz, 3H), 1.08 (d, *J* = 7.2 Hz, 3H);
^{13}C NMR (75 MHz, $CDCl_3$) δ 156.5, 142.5, 104.1, 74.4, 68.2, 30.9, 21.0, 16.5.
Spectroscopic data are in accordance with those reported in literature.

(2*S*,3*R*,4*S*)-2,4-Dimethyl-3,4-dihydro-2*H*-pyran-3-yl carbamate (17b)

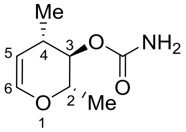

Prepared from **16b** and purified using a cyclohexane/ethyl acetate mixture (3:1) as eluent. The product **17b** was isolated as a white solid (55.6 mg) in 82% yield.

R_f = 0.27 (EtOAc/cyclohexane = 1:3);
mp: 174-176°C;
$[\alpha]_D^{20}$ = +10.9 (c 1.0, MeOH);
^{1}H NMR (300 MHz, methanol-d4) δ 6.27 (dd, *J* = 6.0, 2.4 Hz, 1H), 4.54 (dd, *J* = 6.0, 2.0 Hz, 1H), 4.32 (dd, *J* = 9.3, 9.3 Hz, 1H), 3.80 –3.75 (m, 1H), 2.39 – 2.33 (m, 1H), 1.23 (d, *J* = 6.3 Hz, 3H), 0.99 (d, *J* = 6.9 Hz, 3H);
^{13}C NMR (75 MHz, methanol-d4) δ 159.6, 143.4, 106.0, 78.0, 74.9, 34.8, 18.8, 17.6.
HRMS calcd for $C_8H_{13}NO_3Na$ $(M+Na)^+$: 194.0787 Found: 194.0788.

(2*S*,3*S*)-2-Methyl-3,4-dihydro-2*H*-pyran-3-yl carbamate (24)[6]

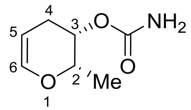

Prepared from **23** and purified using a cyclohexane/ethyl acetate mixture (1:1) as eluent. The product **24** was isolated as a white solid (34.2 mg) in 55% yield.

R_f = 0.36 (EtOAc/cyclohexane = 1:1);
mp 135-137 °C;
$[\alpha]_D^{20}$ = –15.6 (c 1.0, $CHCl_3$); lit. $[\alpha]_D^{20}$ = –30 (c 0.37, $CHCl_3$);
^{1}H NMR (300 MHz, methanol-d4) δ 6.35 –6.31 (m, 1H), 4.89 – 4.85 (m, 1H), 4.64 –4.61 (m, 1H), 4.07 – 4.01 (m, 1H), 2.43 – 2.35 (m, 1H), 2.08 –2.04 (m, 1H), 1.23 (d, *J* = 6.5 Hz, 3H);
^{13}C NMR (75 MHz, methanol-d4) δ 159.6, 144.3, 98.2, 72.6, 69.6, 26.9, 16.6.
HRMS calcd for $C_7H_{12}NO_3Na$ $(M+Na)^+$: 180.0631 Found: 180.0631.
Spectroscopic data are in accordance with those reported in literature.

(2*S*,3*R*)-2-Methyl-3,4-dihydro-2*H*-pyran-3-yl carbamate (27)[6]

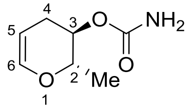

Prepared from alcohol **26** and purified using a cyclohexane/ethyl acetate mixture (2:1) as eluent. The product **27** was isolated as a white solid (40.4 mg) in 65% yield.

R_f = 0.15 (EtOAc/cyclohexane = 1:2);
mp 110-112°C;
$[\alpha]_D^{20}$ = –78 (c 1.0, $CHCl_3$); lit. $[\alpha]_D^{20}$ = –100 (c 0.92, $CHCl_3$);
^{1}H NMR (300 MHz, $CDCl_3$) δ 6.34 – 6.31 (m, 1H), 4.78 – 4.73 (m, 2H), 4.67 –4.63 (m, 1H), 4.64 (m, 1H), 4.02 (dq, *J* = 6.8, 6.4 Hz, 1H), 2.47 –2.39 (m, 1H), 2.15 – 2.07 (m, 1H), 1.28 (d, *J* = 6.4 Hz, 3H);
^{13}C NMR (75 MHz, $CDCl_3$) δ 159.6, 144.3, 98.2, 72.6, 69.6, 26.9, 16.6;
HRMS calcd for $C_7H_{12}NO_3Na$ $(M+Na)^+$: 180.0631 Found: 180.0631.
Spectroscopic data are in accordance with those reported in literature.

General procedure for 2-oxazolidinone preparation (1, 3):

A solution of carbamate **17a** or **24** (1.18 mmol, dried twice by azeotropic removal of toluene), MgO (2.38 mmol, 2.0 equiv), $PhI(OAc)_2$ (1.45 mmol, 1.2 equiv) and $Rh_2(OAc)_4$ (0.103 mmol, 0.09 equiv) in degassed CH_2Cl_2 (4.4 mL) was heated in a sealed tube at 40 °C for 23 h. After cooling, the solution was filtered through a pad of celite and concentrated under reduced pressure. The crude product was purified by flash chromatography to afford the corresponding oxazolidinone .

(3aS,4S,7aS)-4,7a-Dimethyl-1,3a,4,7a-tetrahydro-2*H*-pyrano[4,3-*d*]oxazol-2-one (1)[5]

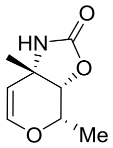

Prepared from **17a** and purified using a cyclohexane/ethyl acetate mixture (6:4) as eluent. The product **1** was isolated as a white solid (245 mg) in 80% yield.

R_f = 0.17 (EtOAc/cyclohexane = 4:6);
mp: 181-183 °C;
$[\alpha]_D^{20}$ = +63 (c 1.0, $CHCl_3$); lit.[x] $[\alpha]_D^{22}$ = +67 (c 0.43, $CHCl_3$);
^{1}H NMR (300 MHz, $CDCl_3$) δ 6.41 (d, *J* = 6.2 Hz, 1H), 6.42 – 6.37 (bs, 1H), 4.74 (dd, *J* = 6.2, 1.5 Hz, 1H), 4.20 (s, 1H), 3.99 – 3.84 (m, 1H), 1.46 (d, *J* = 6.6 Hz, 3H), 1.43 (s, 3H);
^{13}C NMR (75 MHz, $CDCl_3$) δ 158.4, 144.3, 105.3, 82.8, 68.6, 53.6, 26.6, 17.0;
HRMS calcd for $C_8H_{11}NO_3Na$ $(M+Na)^+$: 192.0631 Found: 192.0632.
Spectroscopic data are in accordance with those reported in literature.

(3aS,4S,7aS)-4-Methyl-1,3a,4,7a-tetrahydro-2*H*-pyrano[4,3-*d*]oxazol-2-one (3)[6]

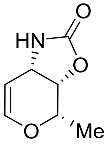

Prepared from **24** and purified using a cyclohexane/ethyl acetate mixture (1:2) as eluent. The product **3** was isolated as a white solid (137 mg) in 49% yield.

R_f = 0.22 (EtOAc/cyclohexane = 2:1);
mp: 123-125 °C;
$[\alpha]_D^{20}$ = –54 (c 1.0, $CHCl_3$); lit. $[\alpha]_D^{20}$ = –50 (c 0.58, $CHCl_3$)
^{1}H NMR (300 MHz, acetone-d6) δ 6.68 – 6.66 (bs, 1H), 6.55 (d, *J* = 6.3 Hz, 1H), 4.82 (ddd, *J* = 6.3, 2.7, 1.2 Hz, 1H), 4.69 (d, *J* = 7.6 Hz, 1H), 4.25 (dd, *J* = 7.6, 2.4 Hz, 1H), 4.02 (q, *J* = 6.3 Hz, 1H), 1.38 (d, *J* = 6.3 Hz, 3H); ^{13}C NMR (75 MHz, acetone-d6) δ 158.8, 146.9, 102.3, 77.2, 70.9, 47.2, 17.2.
HRMS calcd for $C_7H_9NO_3Na$ $(M+Na)^+$: 178.0474 Found: 178.0474.
Spectroscopic data are in accordance with those reported in literature.

References

1) Shimano, M.; Kamei, N.; Shibata, T.; Inogushi, K.; Itoh, N.; Ikari, T.; Senda, H. *Tetrahedron* **1998**, *54*, 12745-12774.
2) Roush, W.; Bennett, C. E.; Roberts, S. E. *J. Org. Chem*. **2001**, *66*, 6389-6393.
3) Ramirez-Fernandez, J.; Botubol, J. M.; Bustillo, A. J.; Aleu, J.; Collado, I. G.; Hernandez-Galan, R. *Nat. Prod. Comun*. **2011**, *6*, 443-450.
4) Schmidt, B.; Biernat, A. *Chem. Eur. J*. **2008**, *14*, 6135-6141.
5) Parker, K. A.; Chang, W. *Org. Lett*. **2003**, *5*, 3891-3893.
6) Parker, K. A.; Chang, W. *Org. Lett*. **2005**, *7*, 1785-1788.

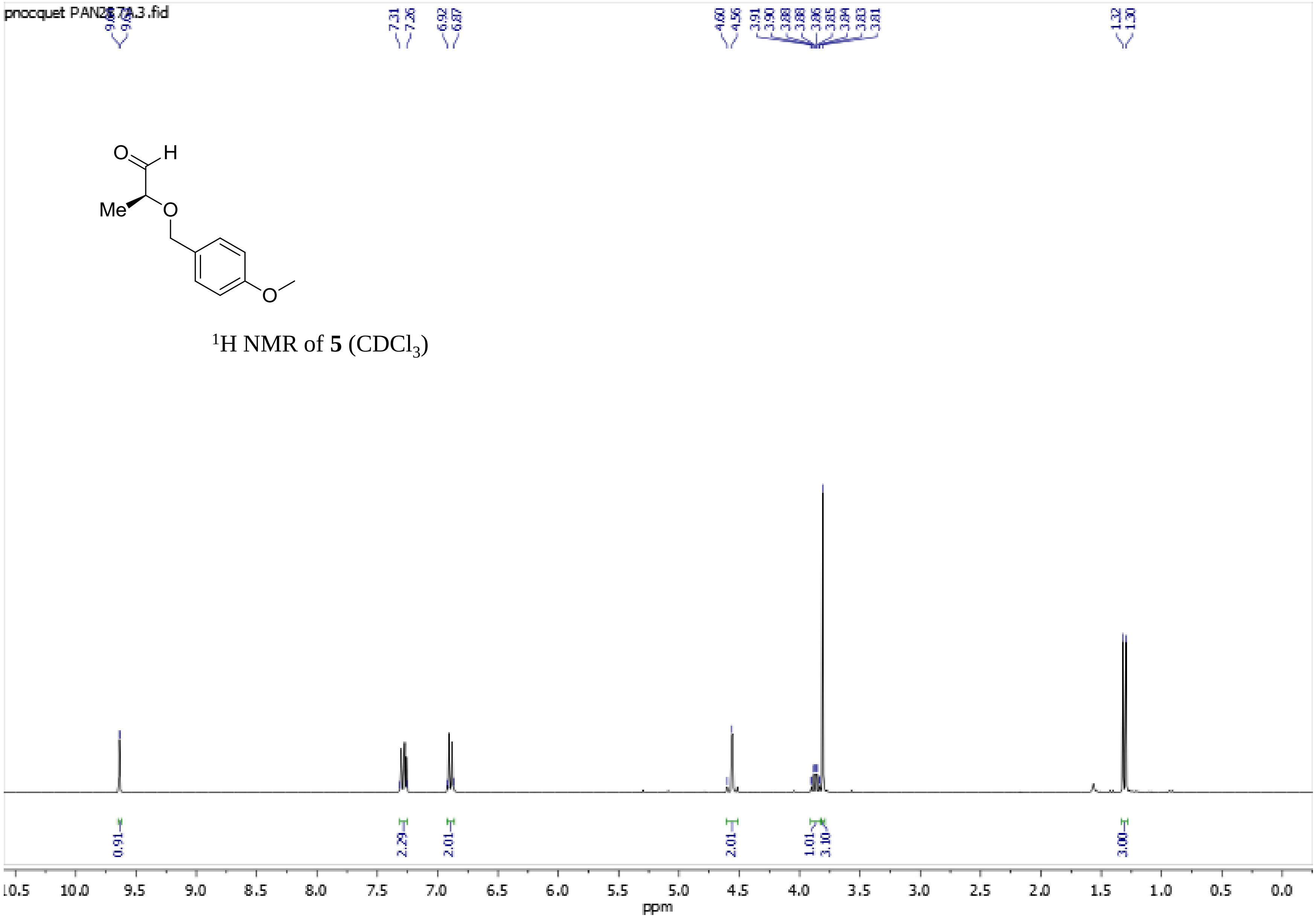

^{1}H NMR of **5** ($CDCl_3$)

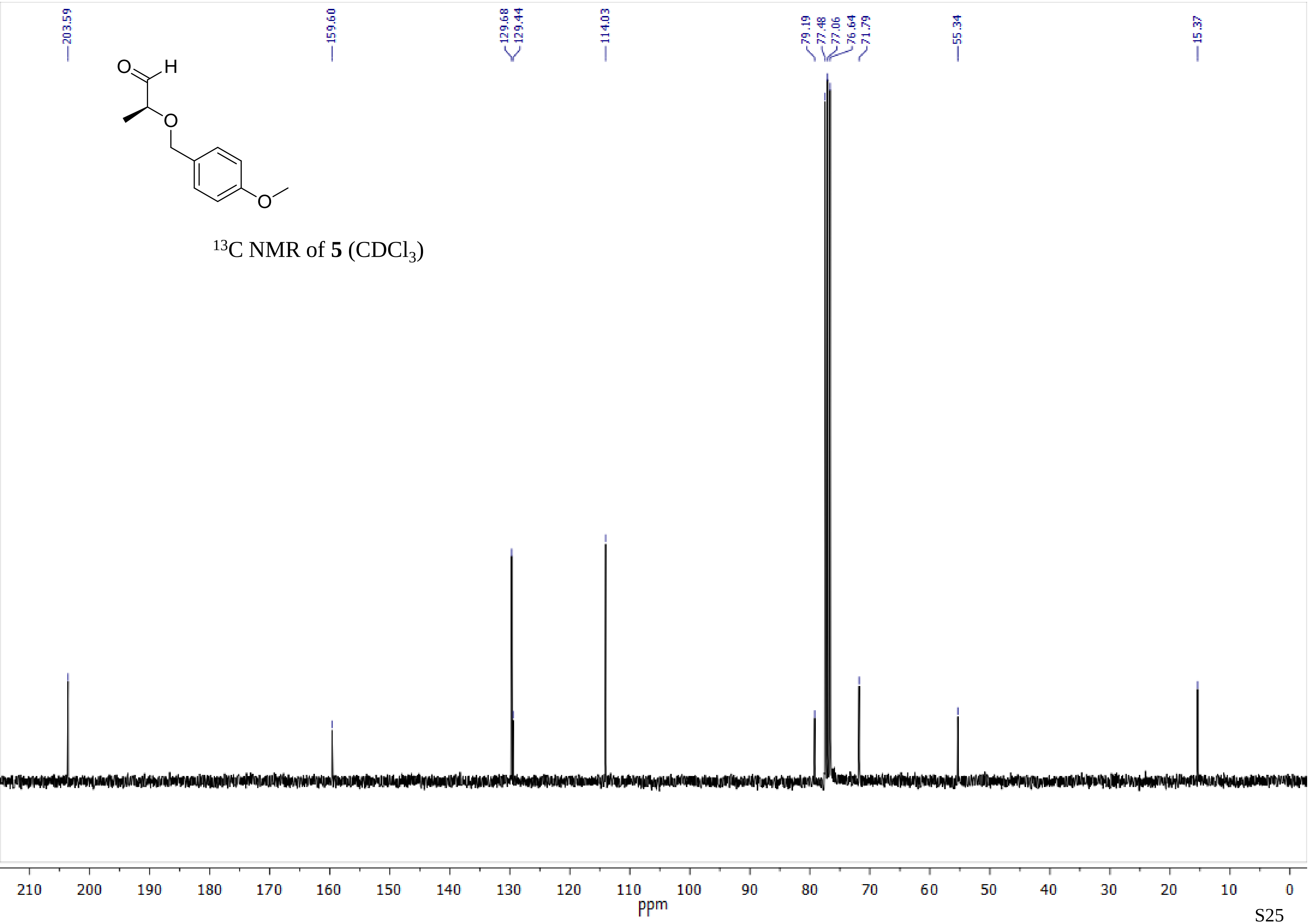

^{13}C NMR of **5** ($CDCl_3$)

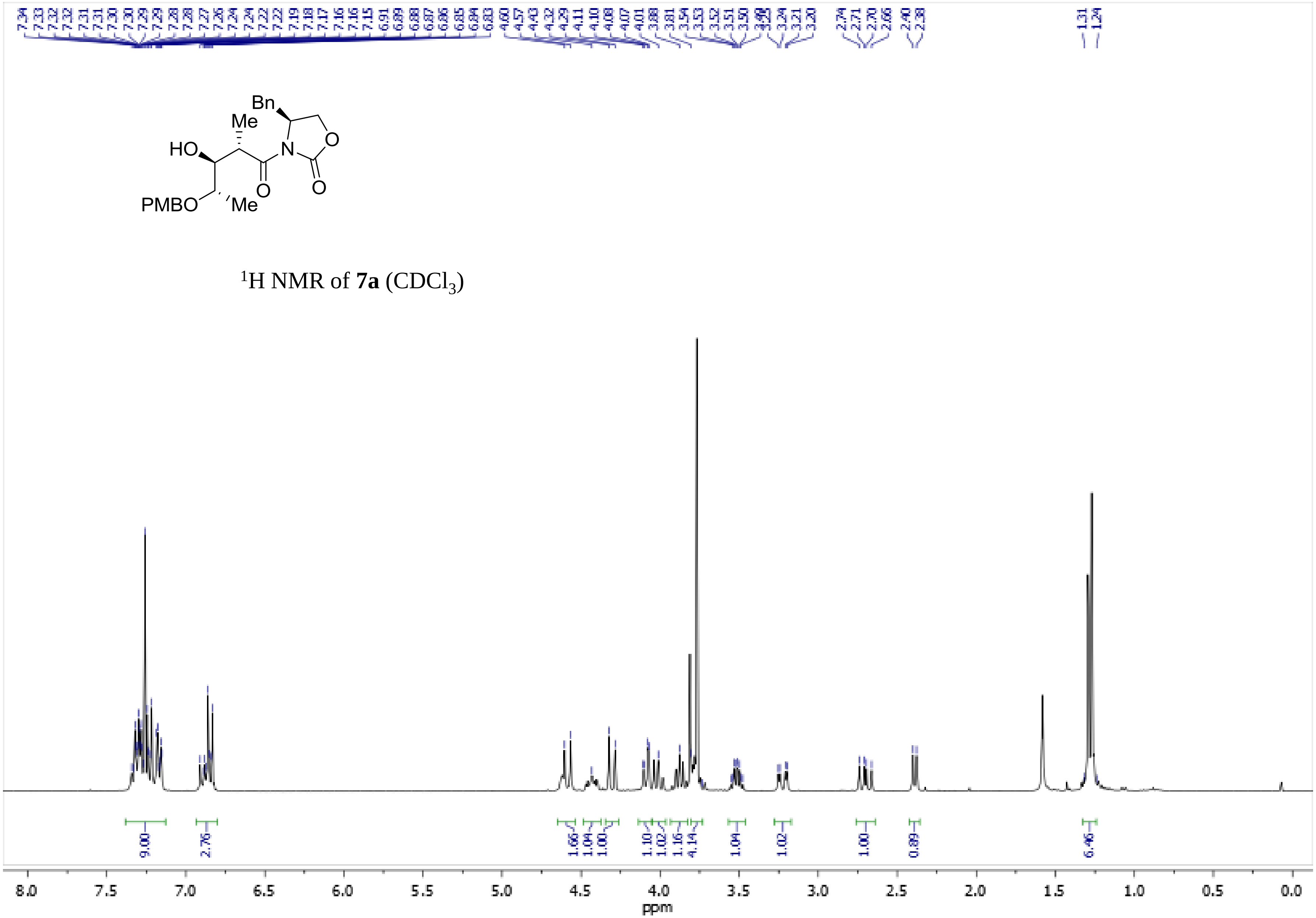

^{1}H NMR of **7a** ($CDCl_3$)

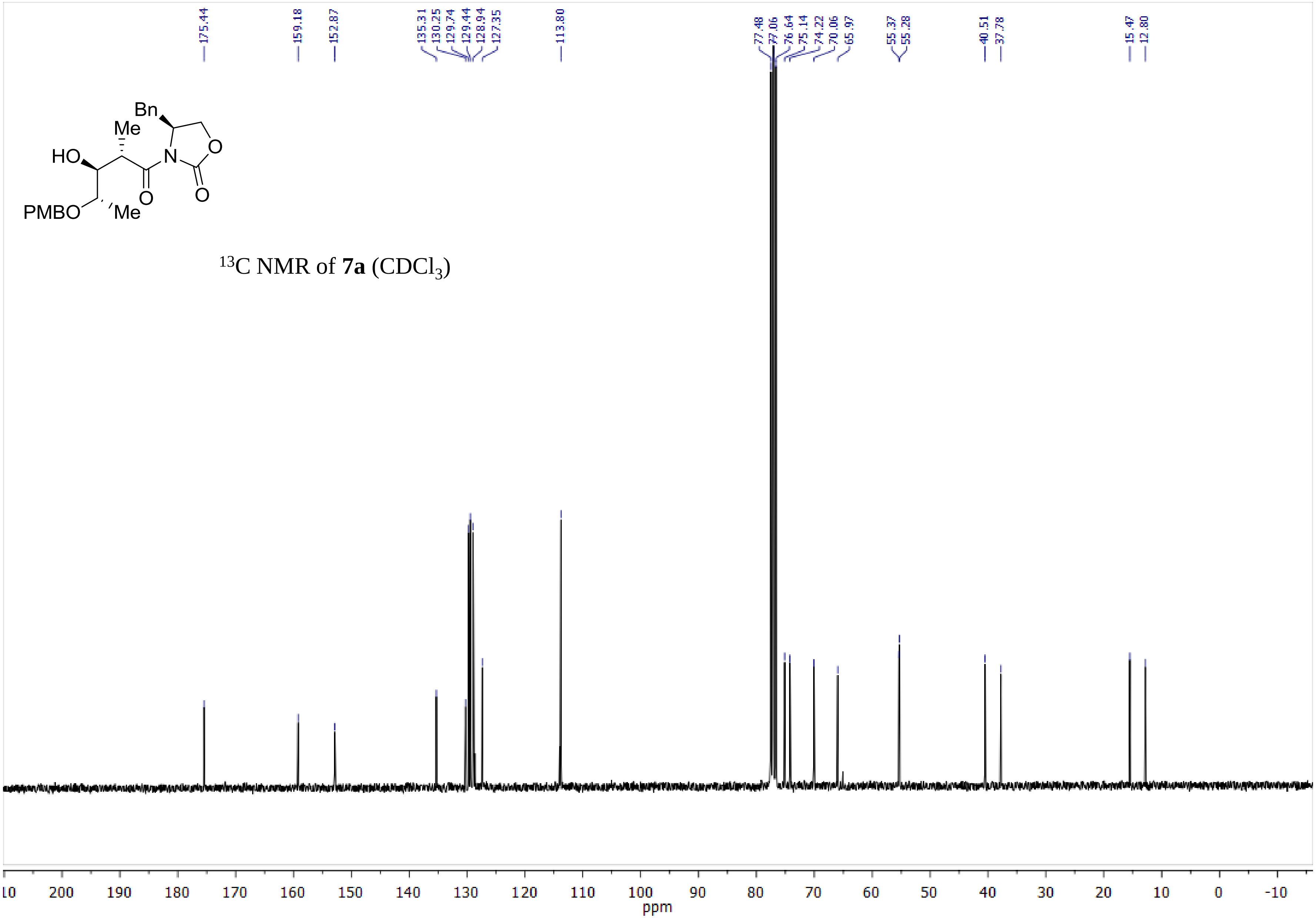

^{13}C NMR of **7a** ($CDCl_3$)

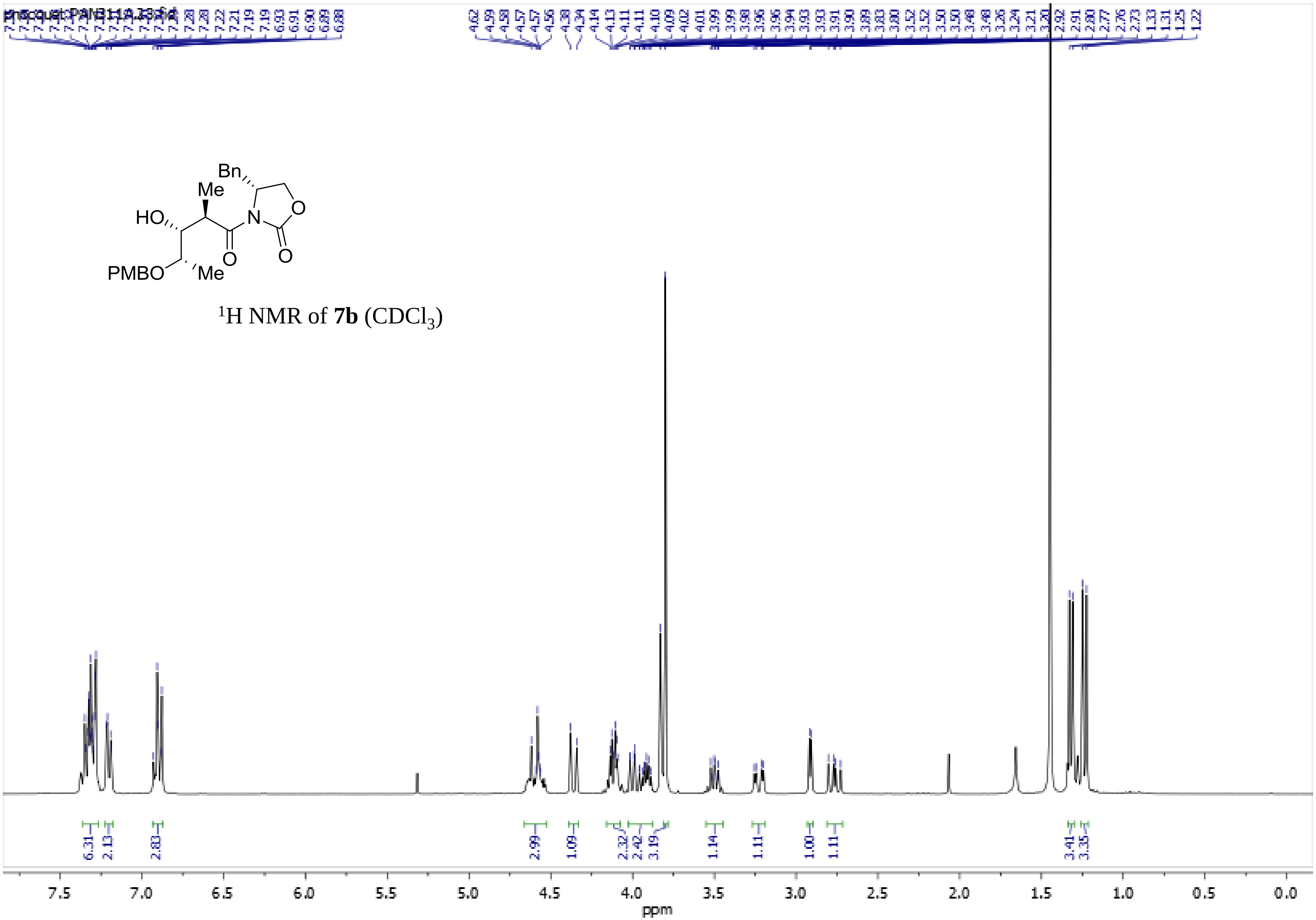

^{1}H NMR of **7b** ($CDCl_3$)

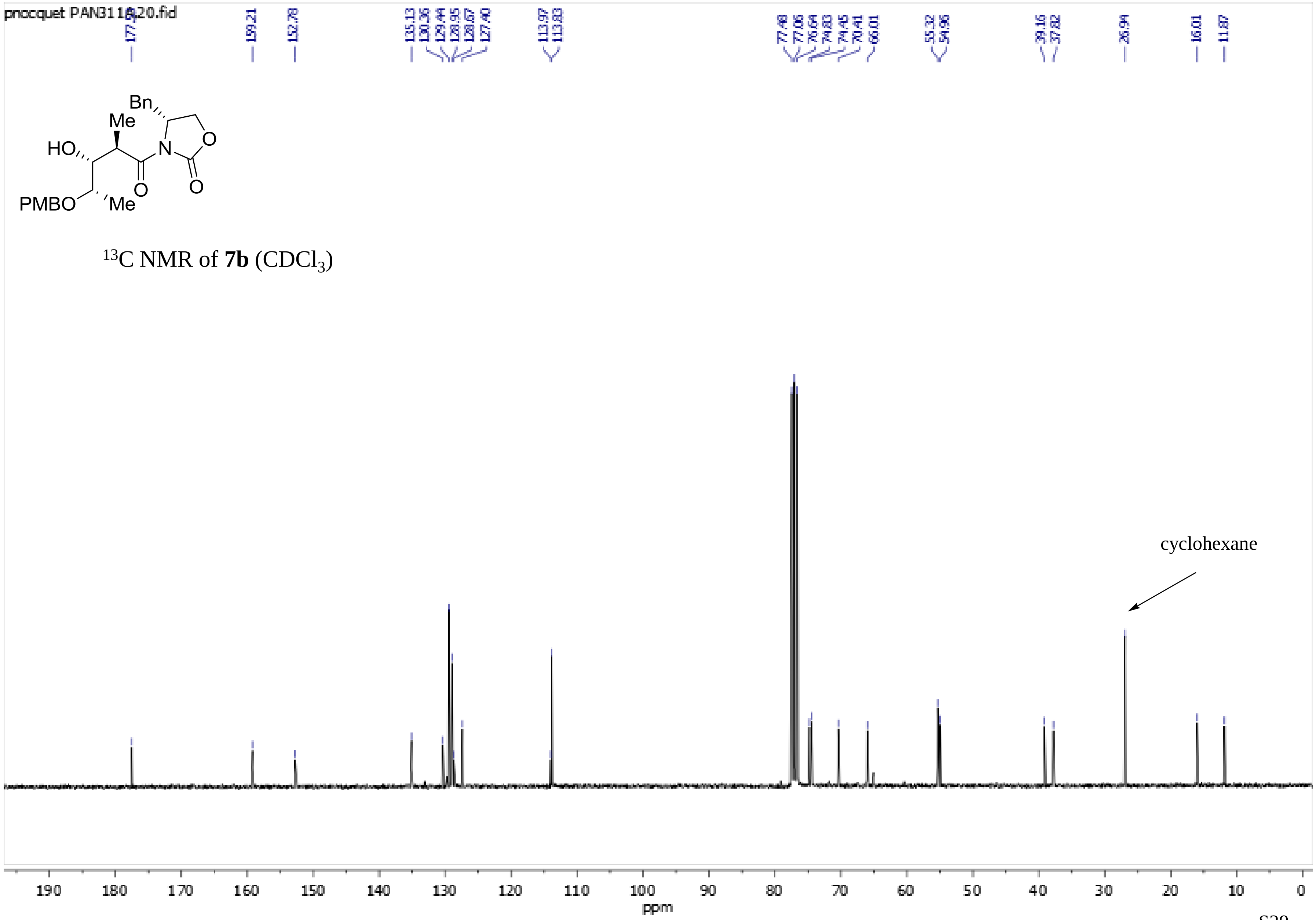

^{13}C NMR of **7b** ($CDCl_3$)

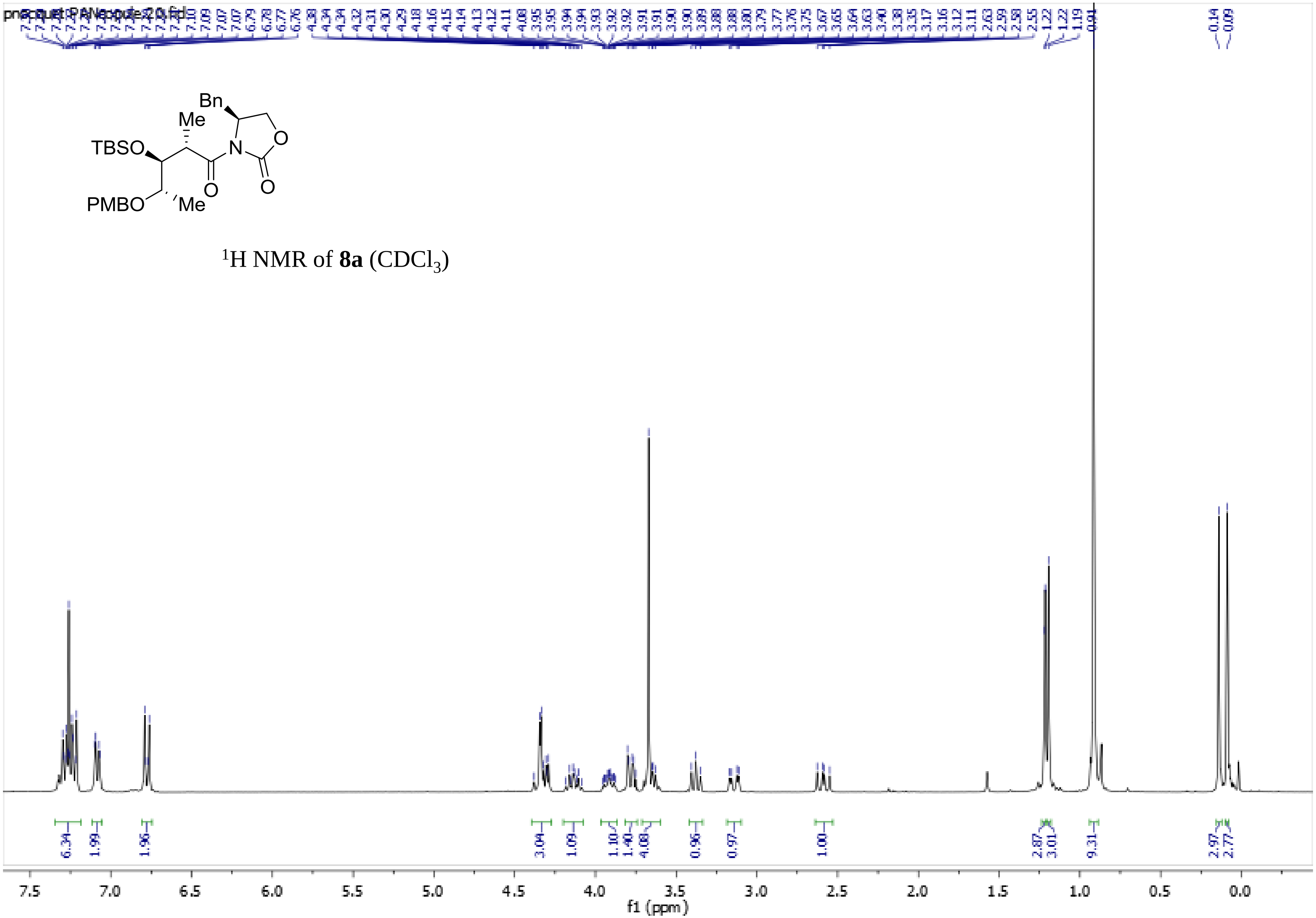

^{1}H NMR of **8a** ($CDCl_3$)

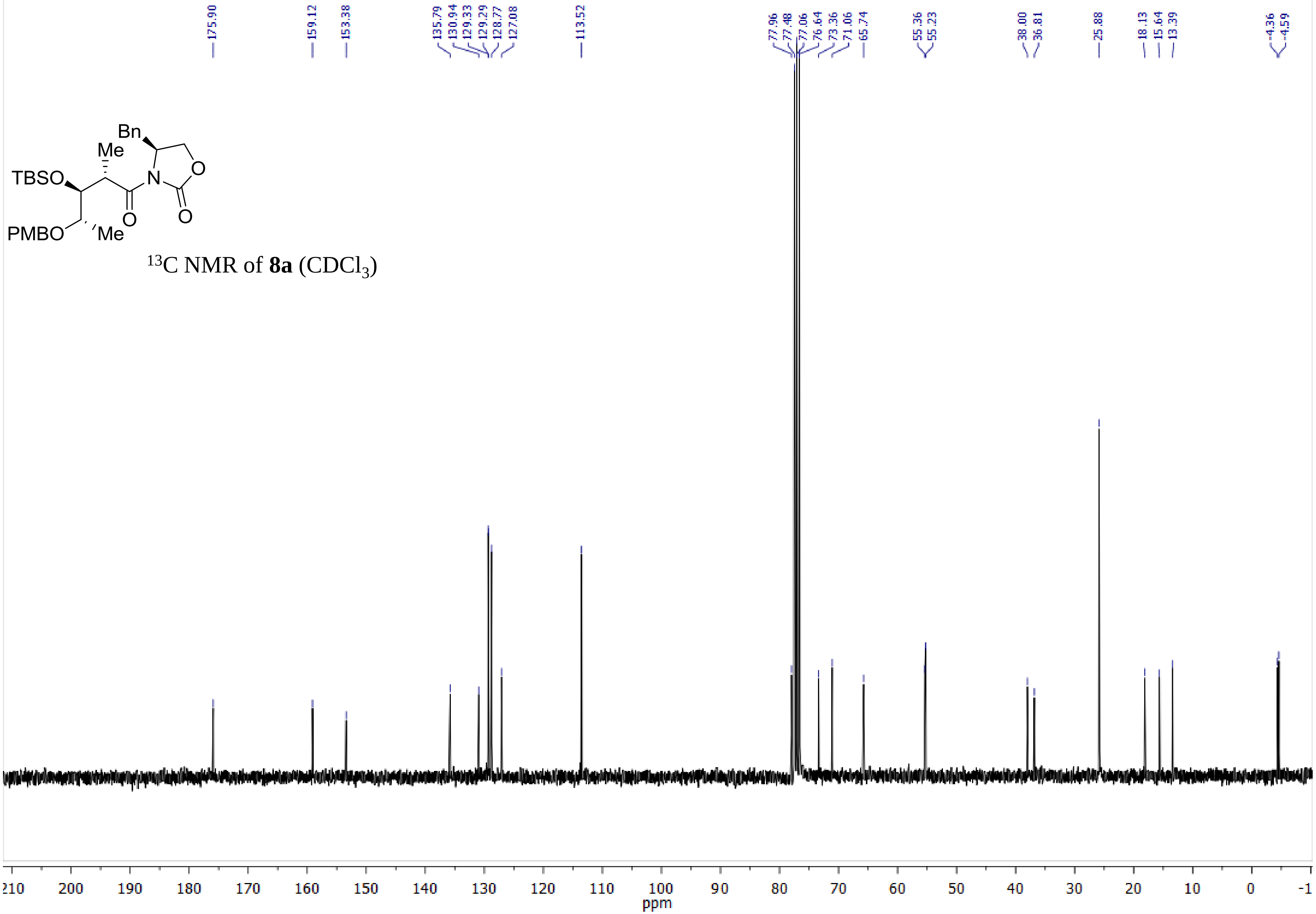

^{13}C NMR of **8a** ($CDCl_3$)

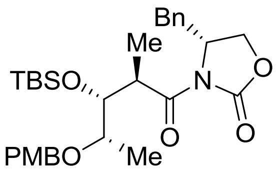

^{1}H NMR of **8b** ($CDCl_3$)

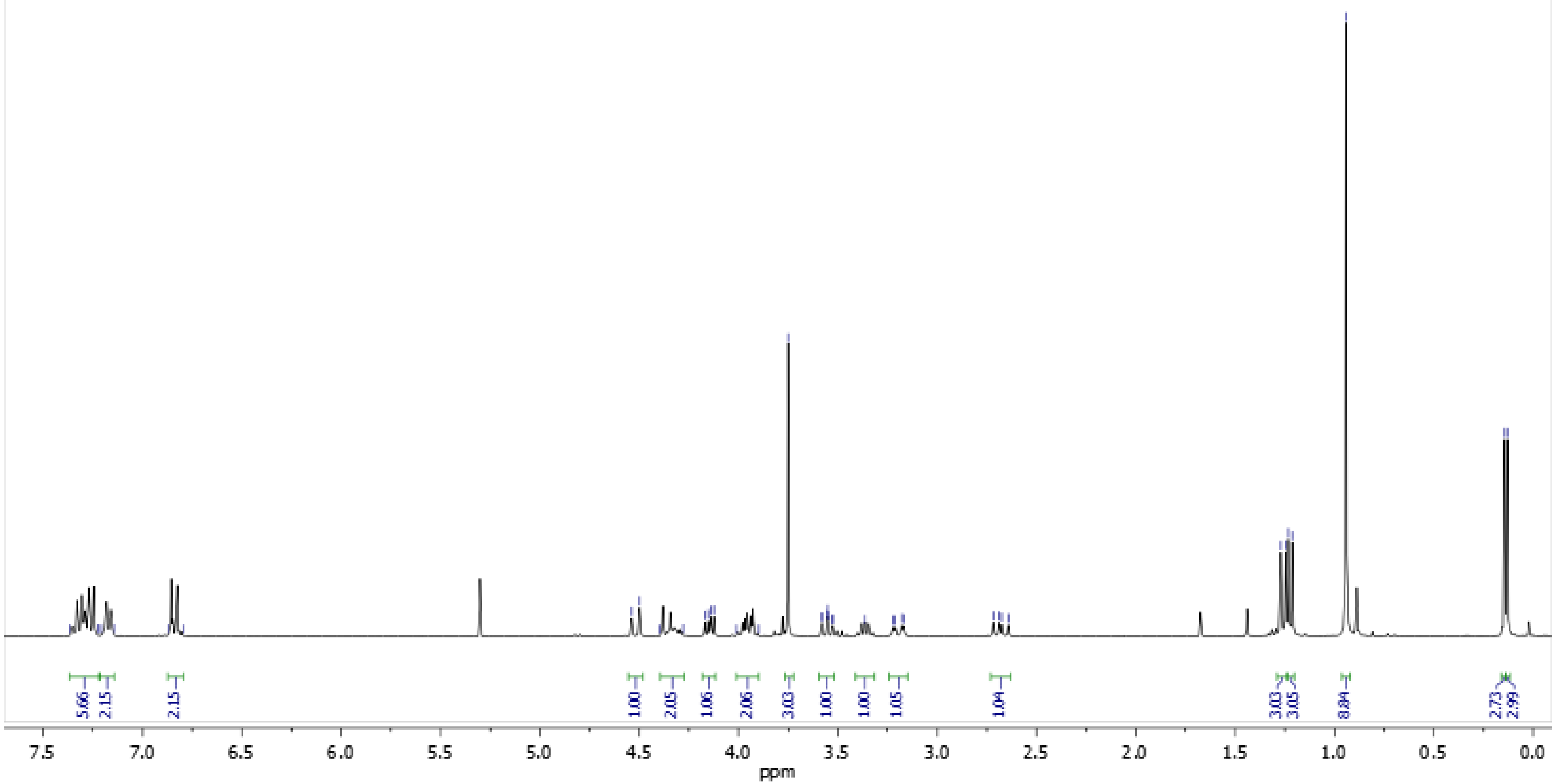

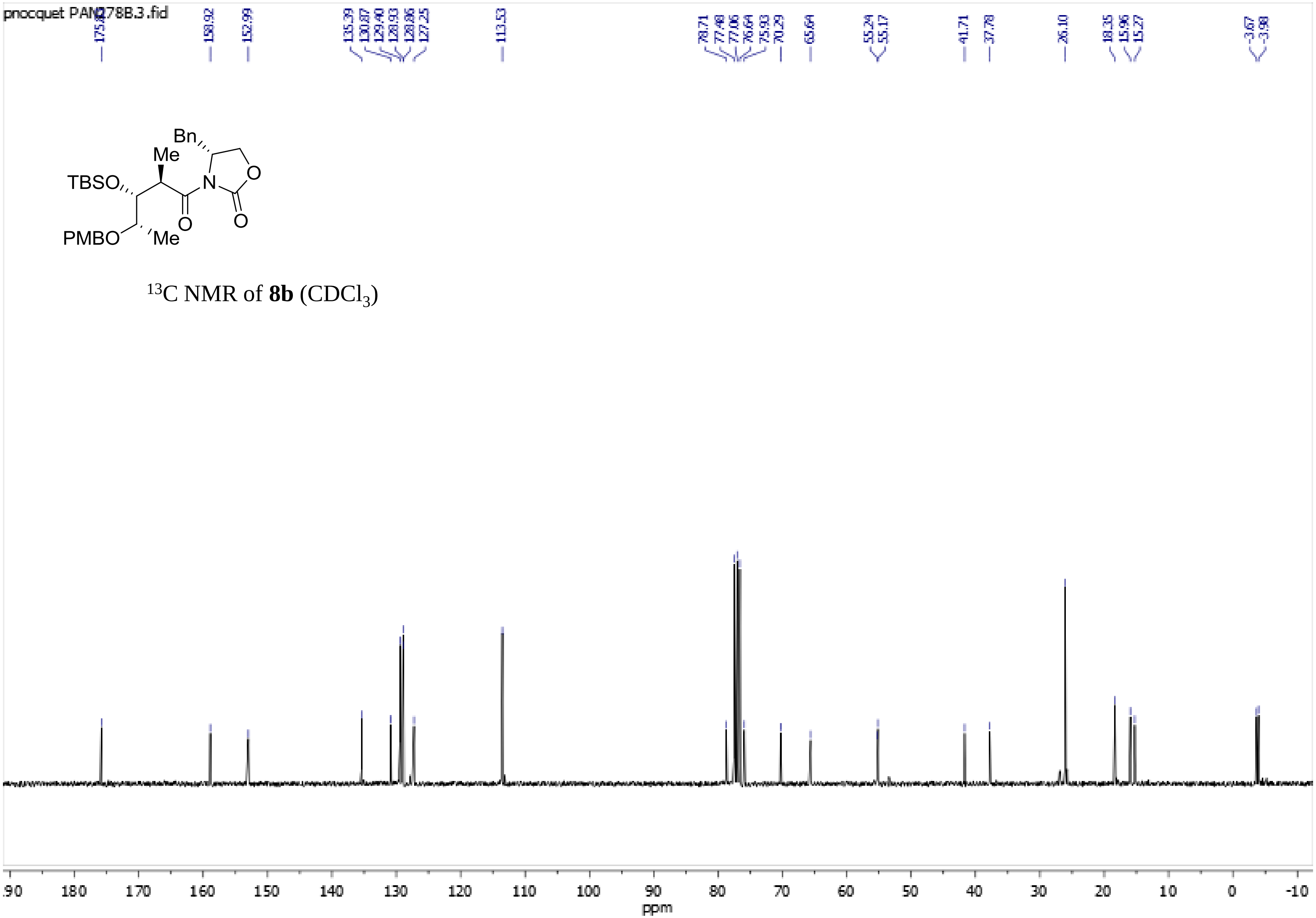

^{13}C NMR of **8b** ($CDCl_3$)

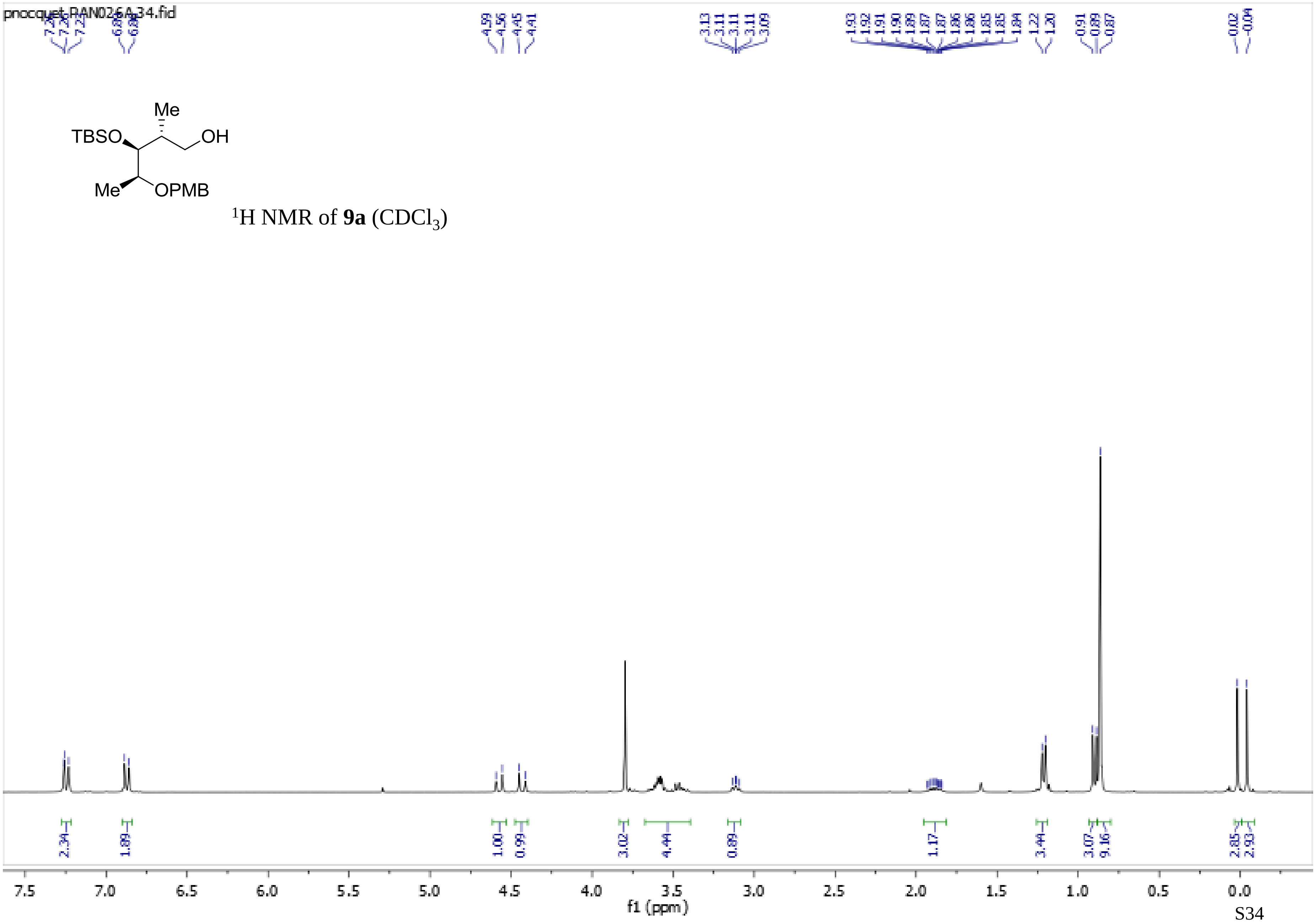

^{1}H NMR of **9a** ($CDCl_3$)

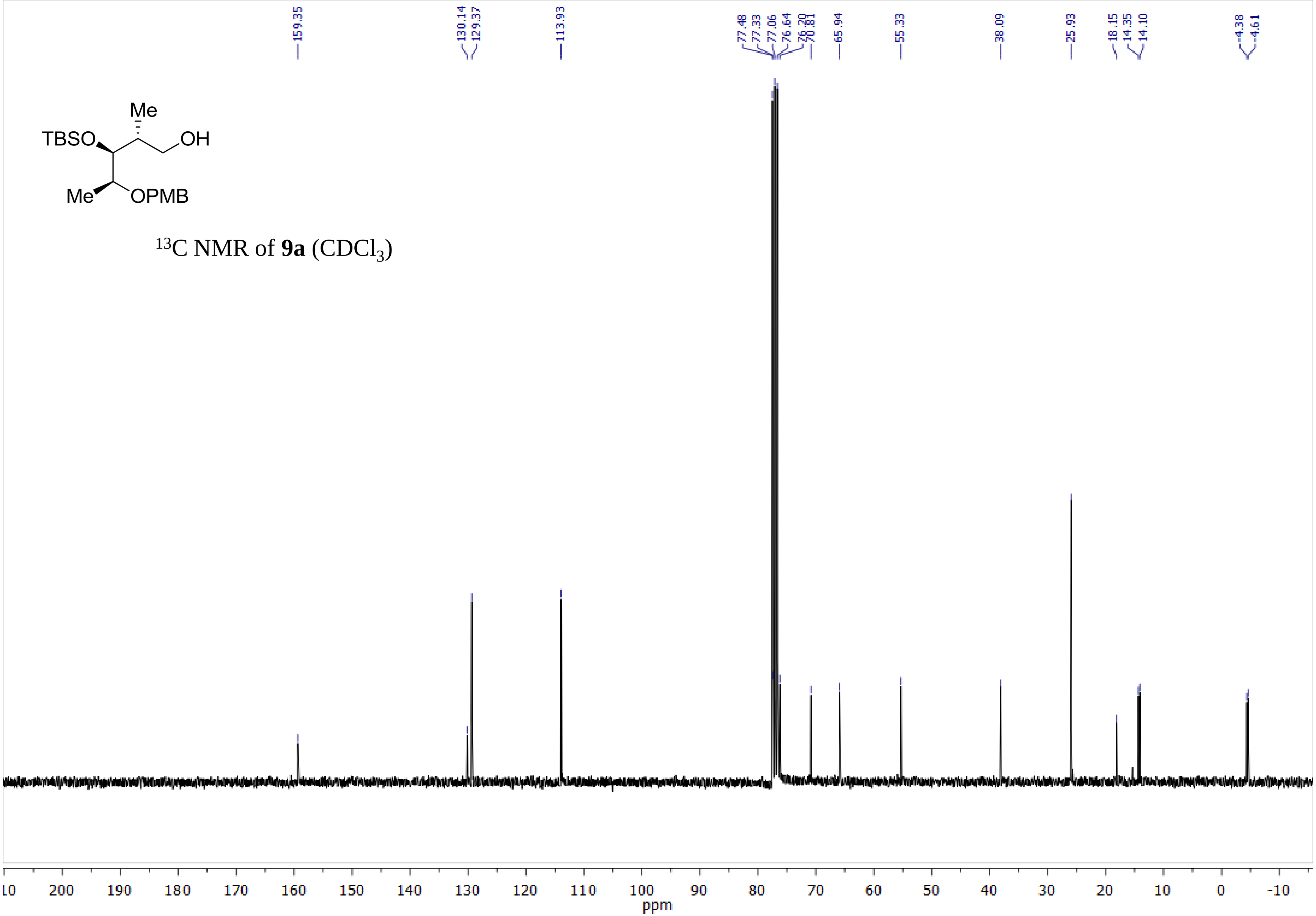

^{13}C NMR of **9a** ($CDCl_3$)

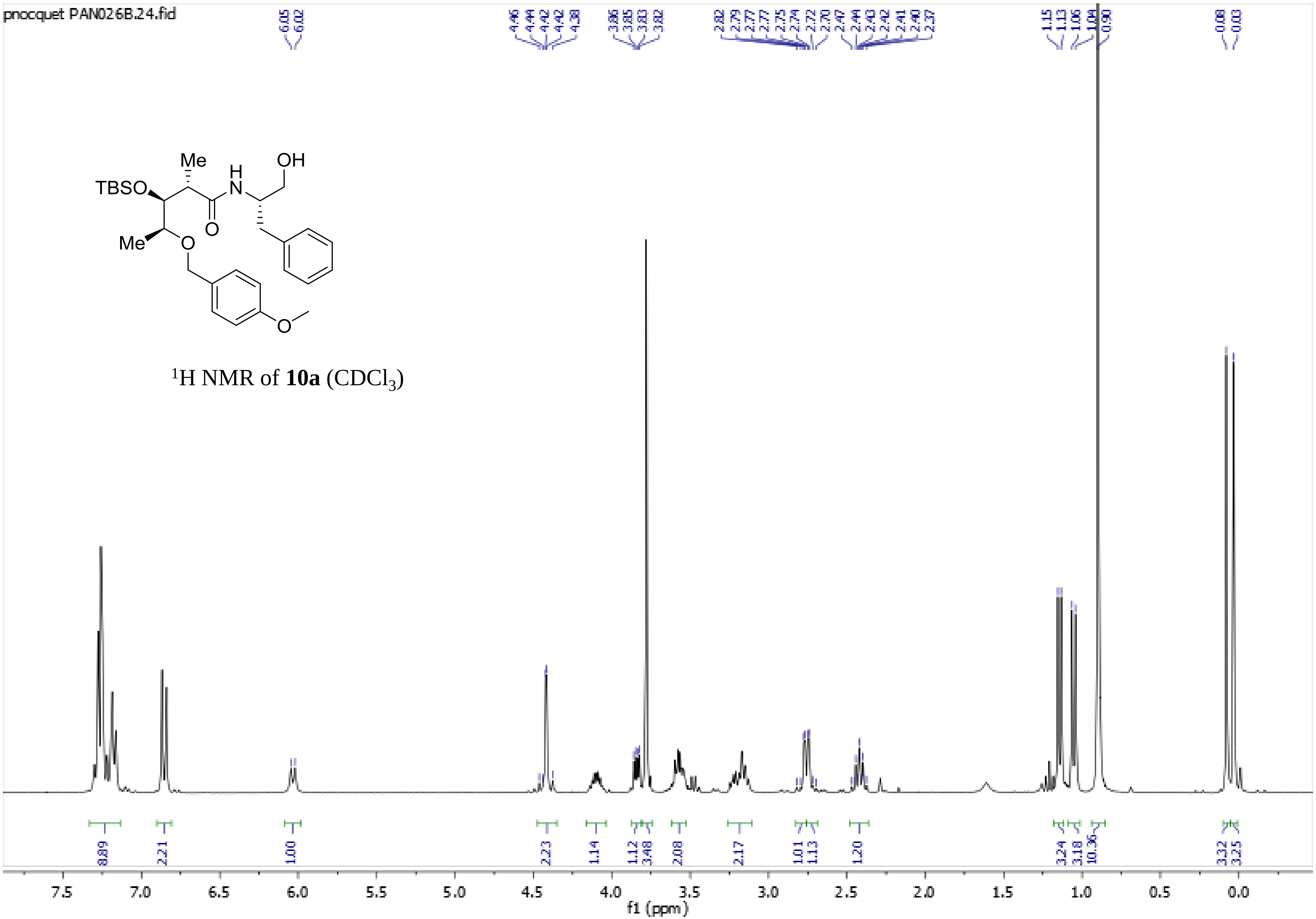

^{1}H NMR of **10a** ($CDCl_3$)

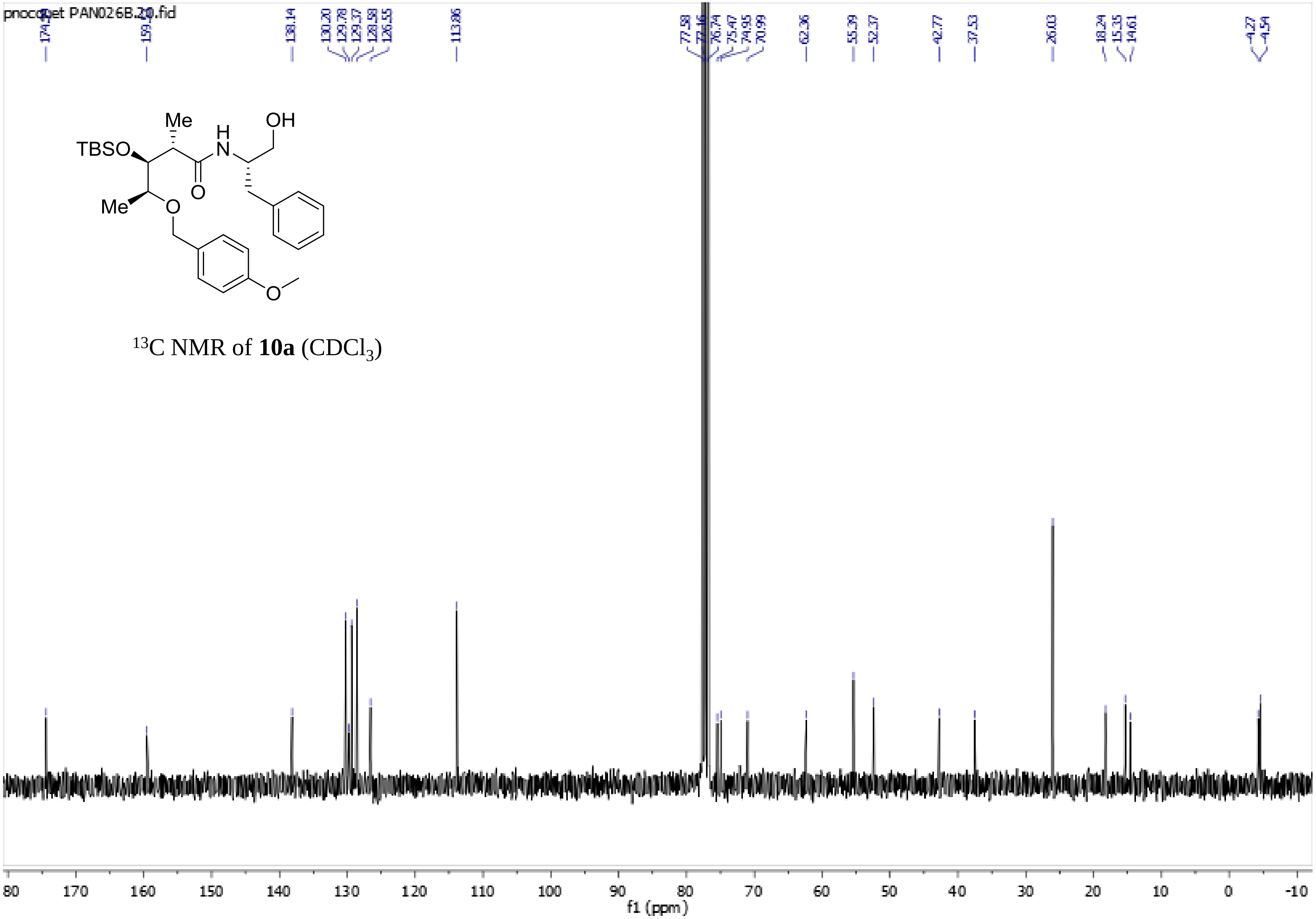

^{13}C NMR of **10a** ($CDCl_3$)

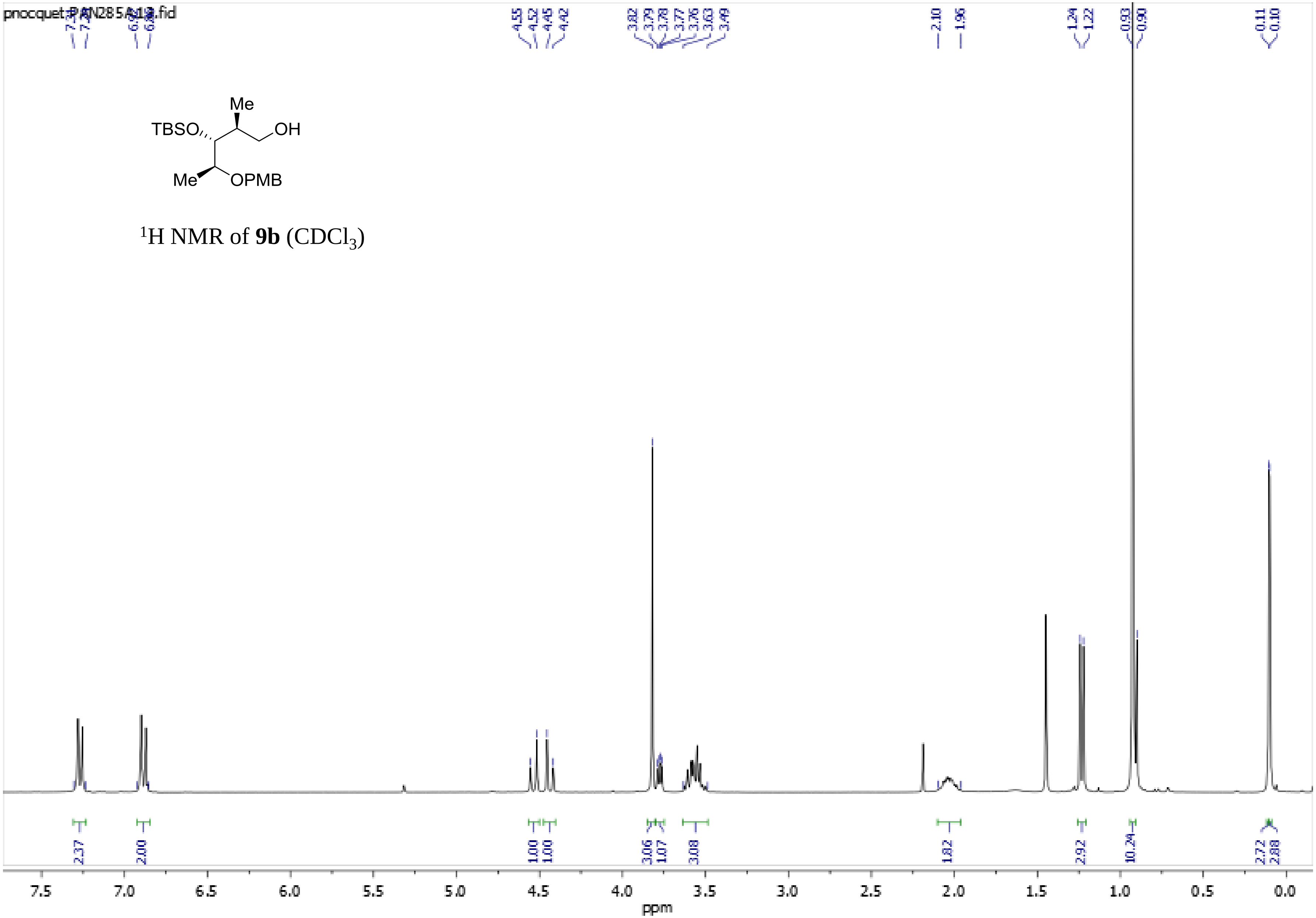

^{1}H NMR of **9b** ($CDCl_3$)

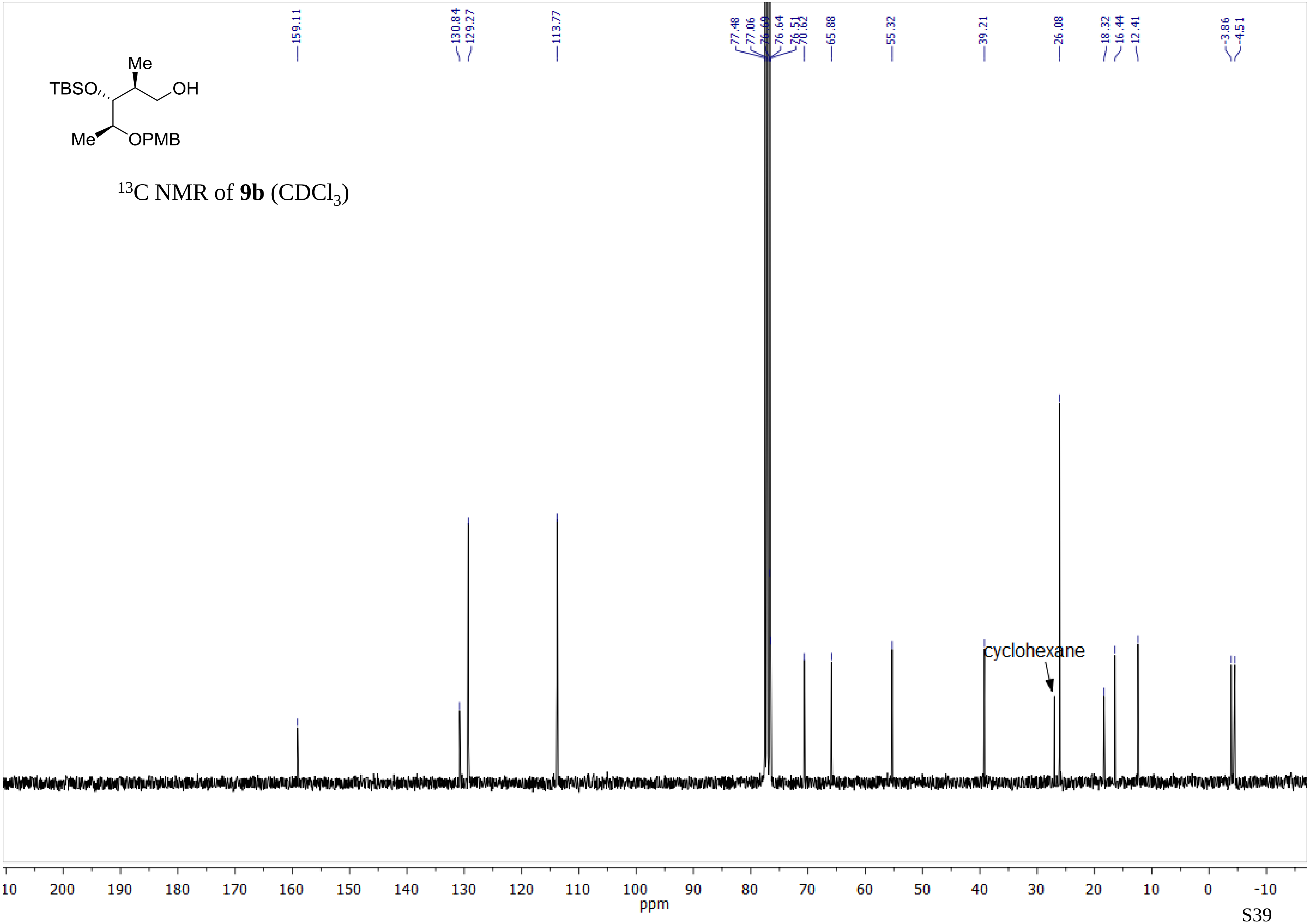

^{13}C NMR of **9b** ($CDCl_3$)

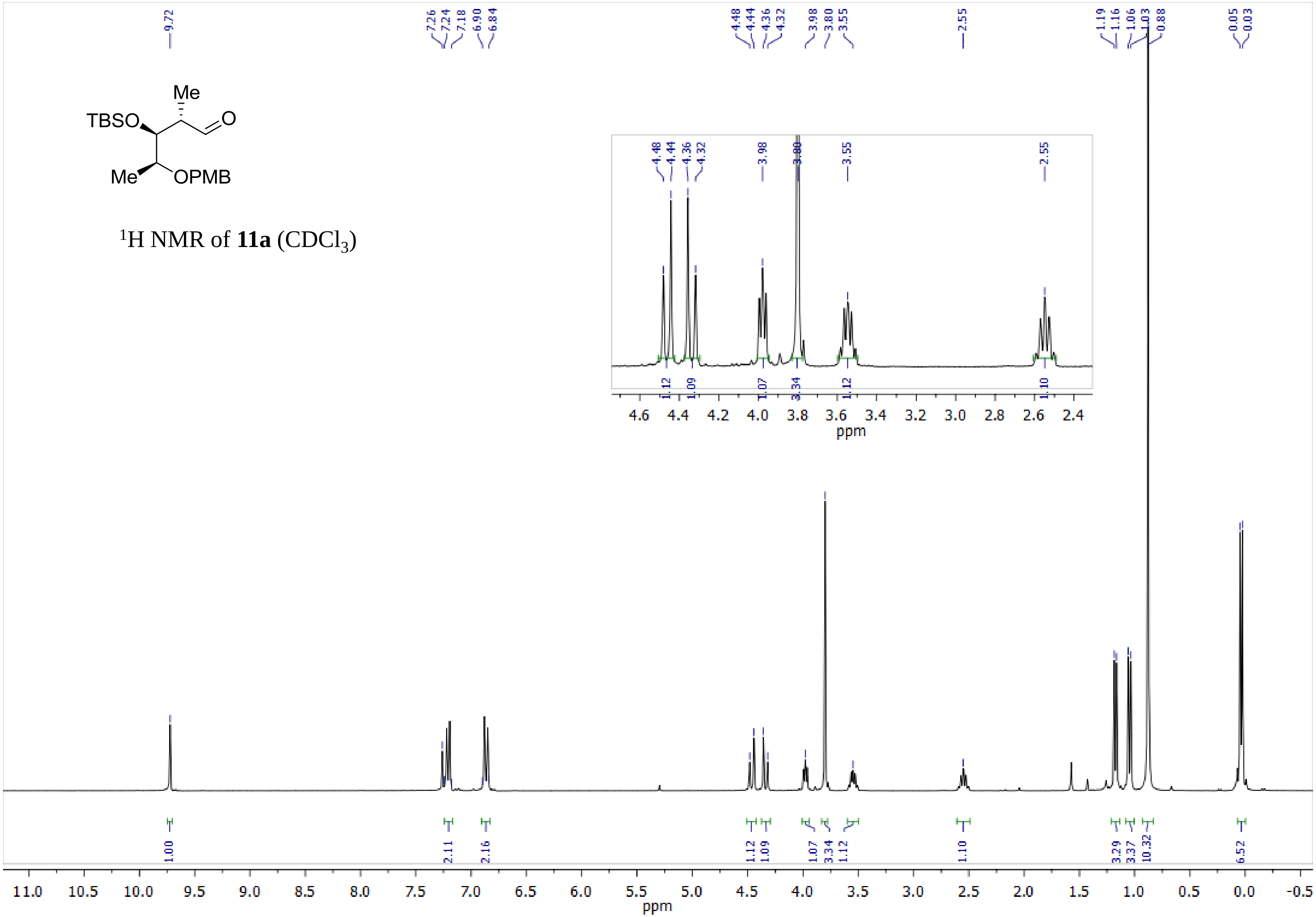

^{1}H NMR of **11a** ($CDCl_3$)

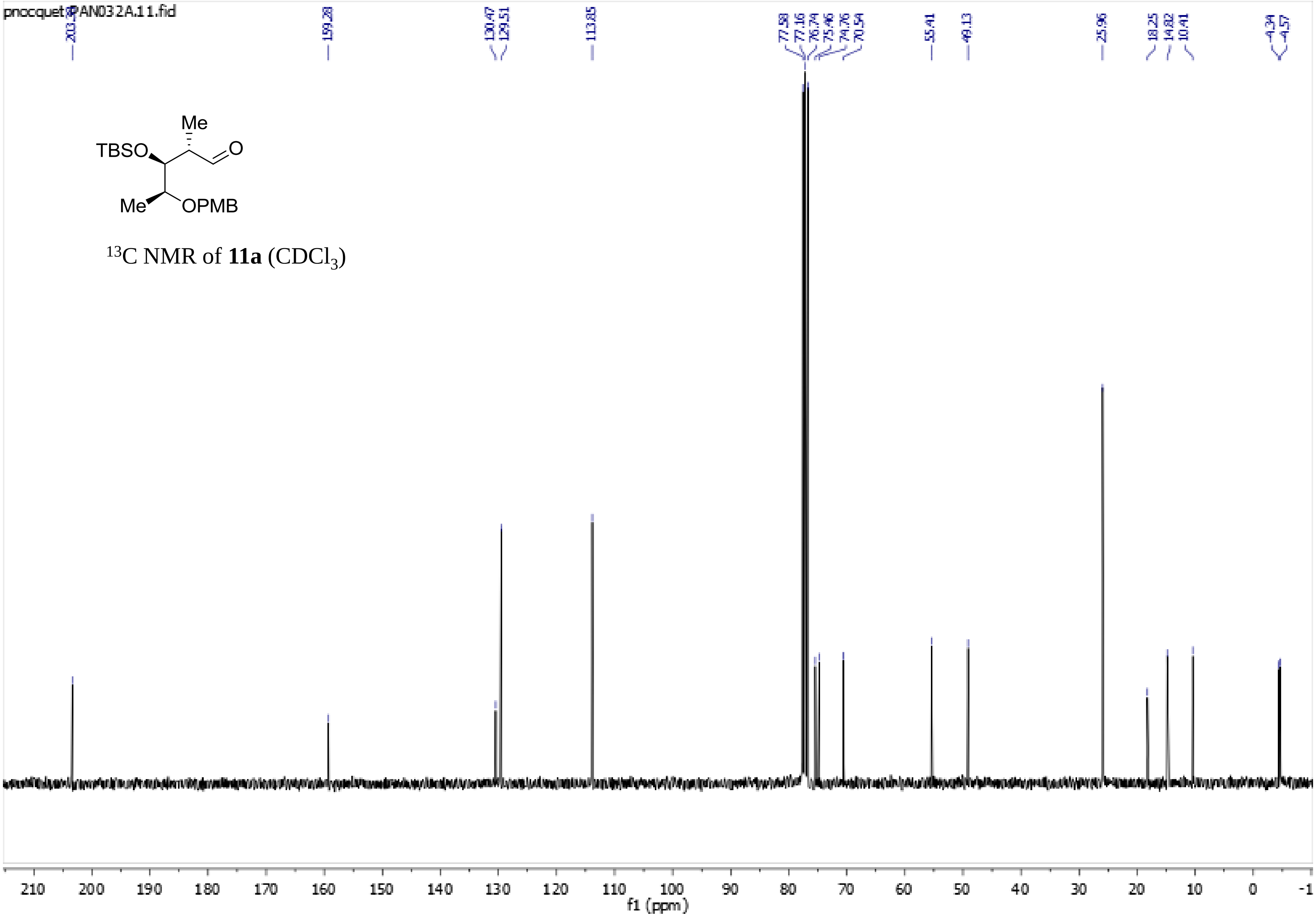

^{13}C NMR of **11a** ($CDCl_3$)

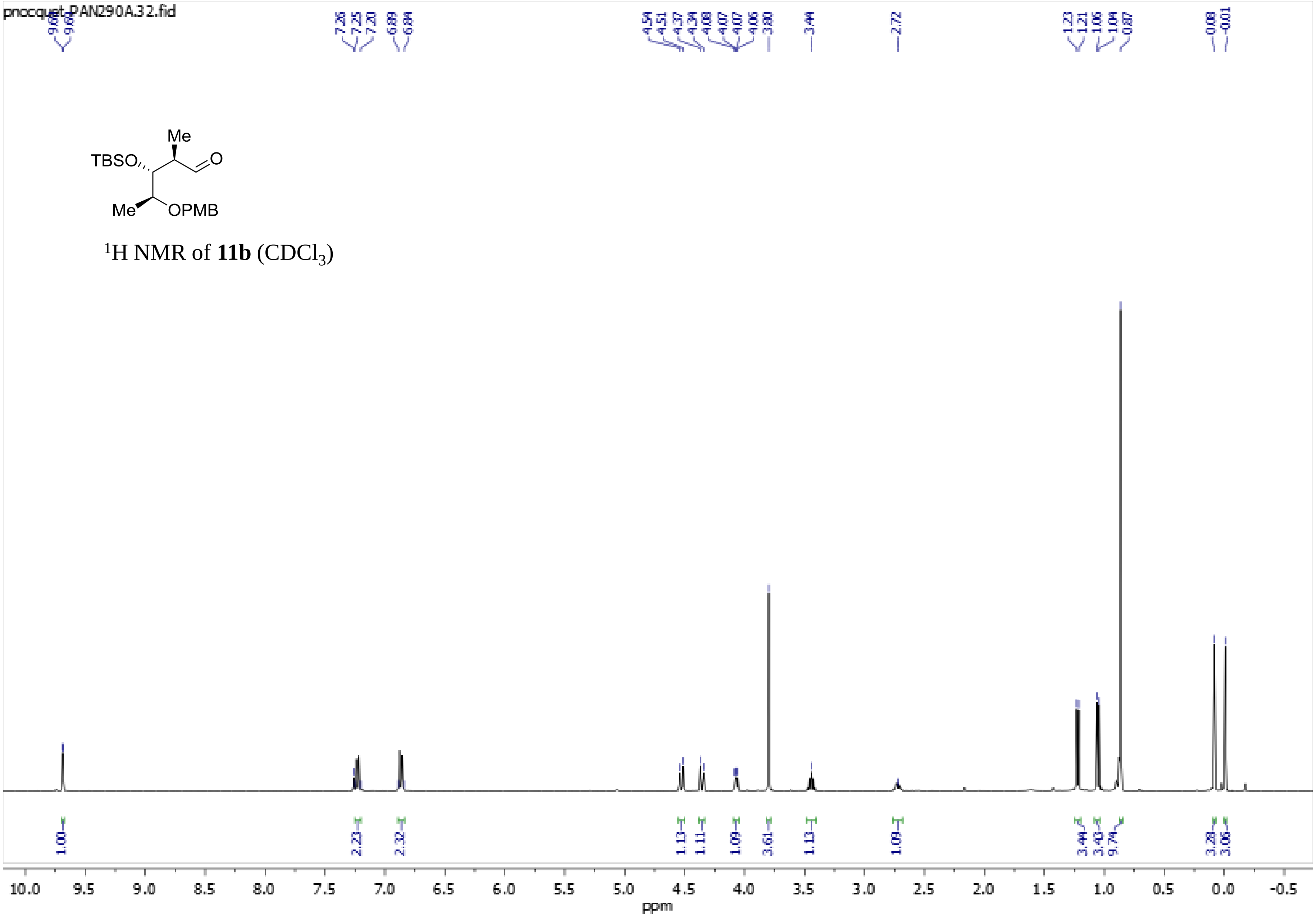

^{1}H NMR of **11b** ($CDCl_3$)

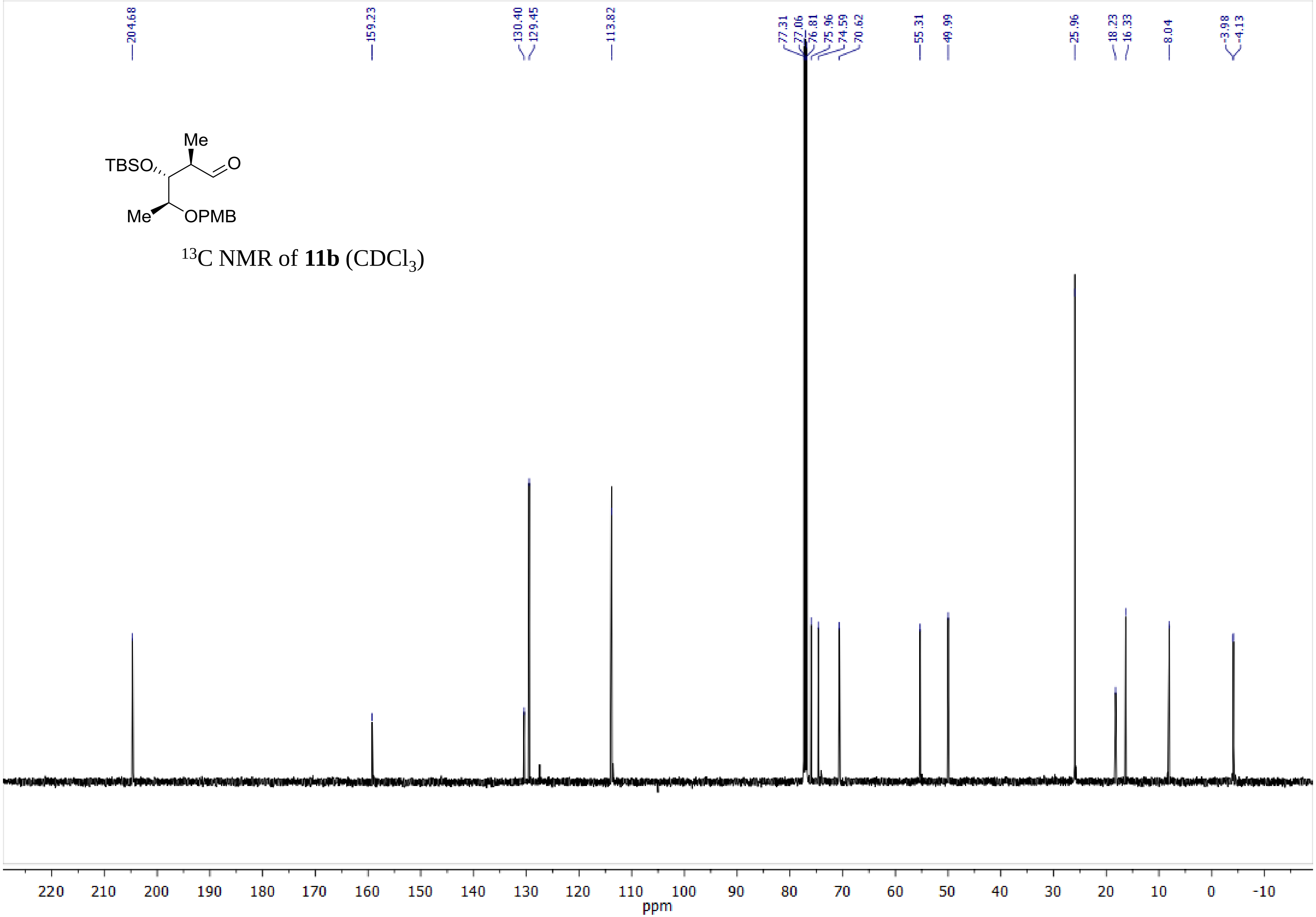

^{13}C NMR of **11b** ($CDCl_3$)

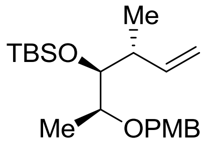

^{1}H NMR of **13a** ($CDCl_3$)

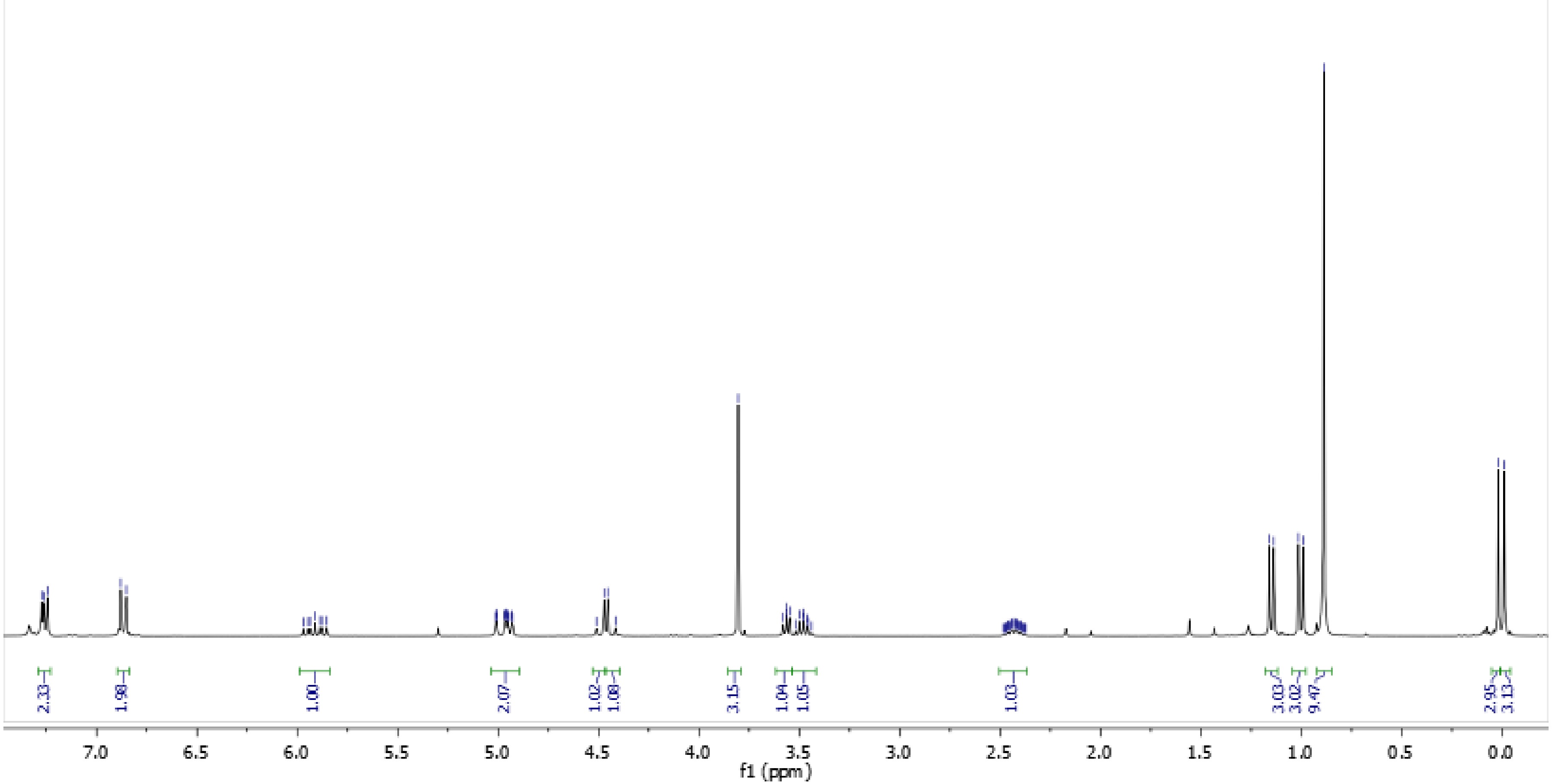

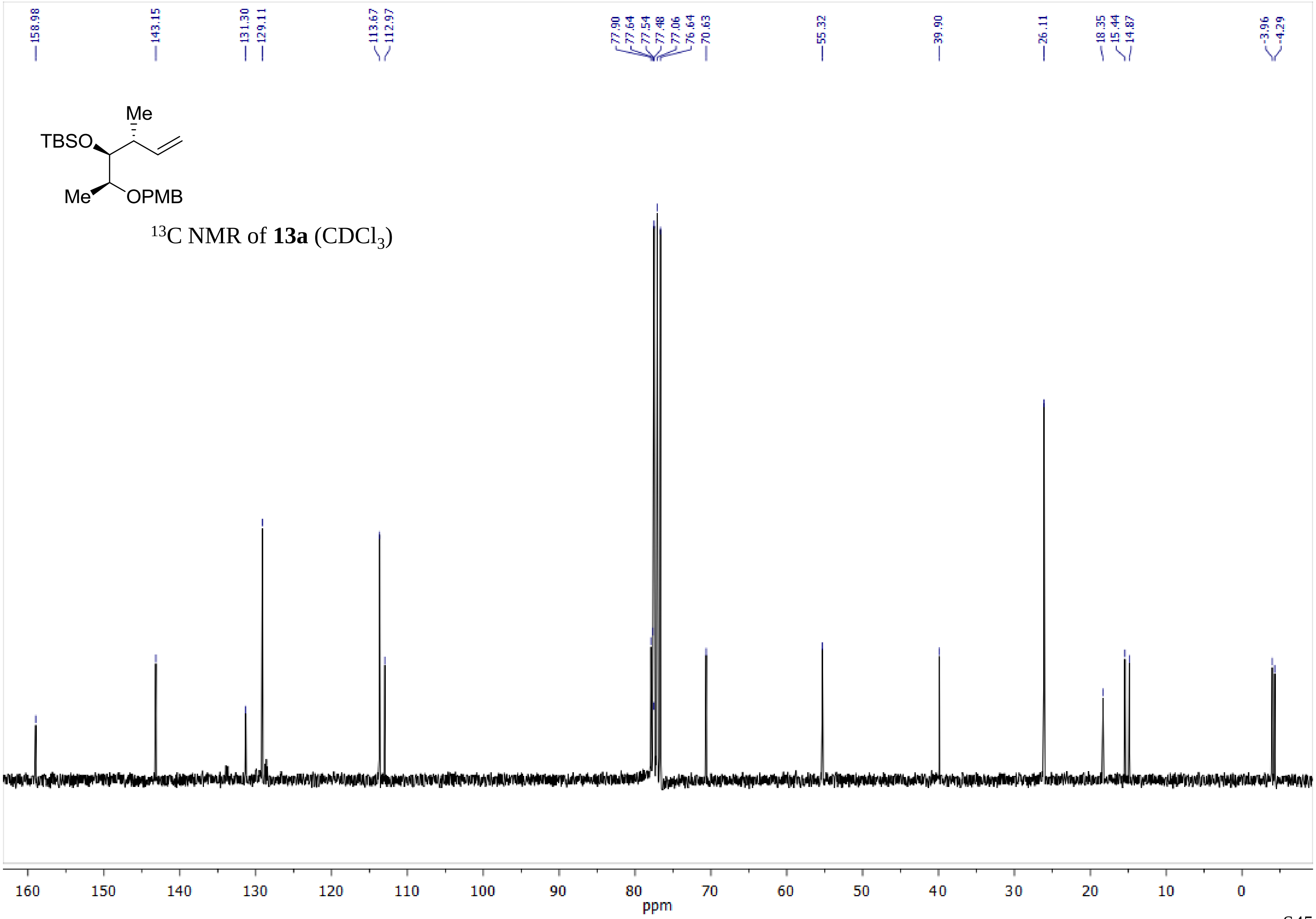

^{13}C NMR of **13a** ($CDCl_3$)

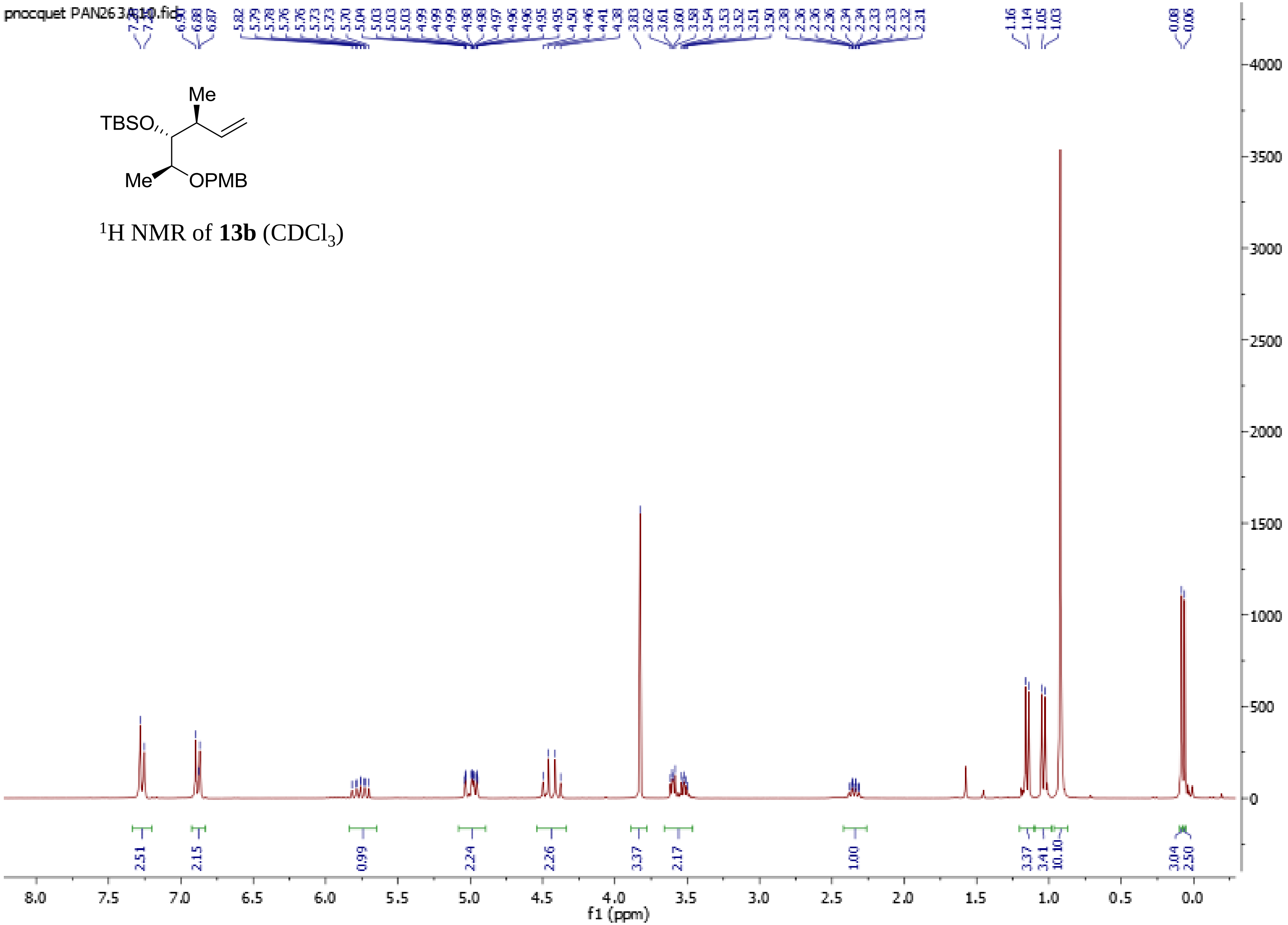

^{1}H NMR of **13b** ($CDCl_3$)

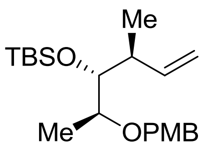

^{13}C NMR of **13b** ($CDCl_3$)

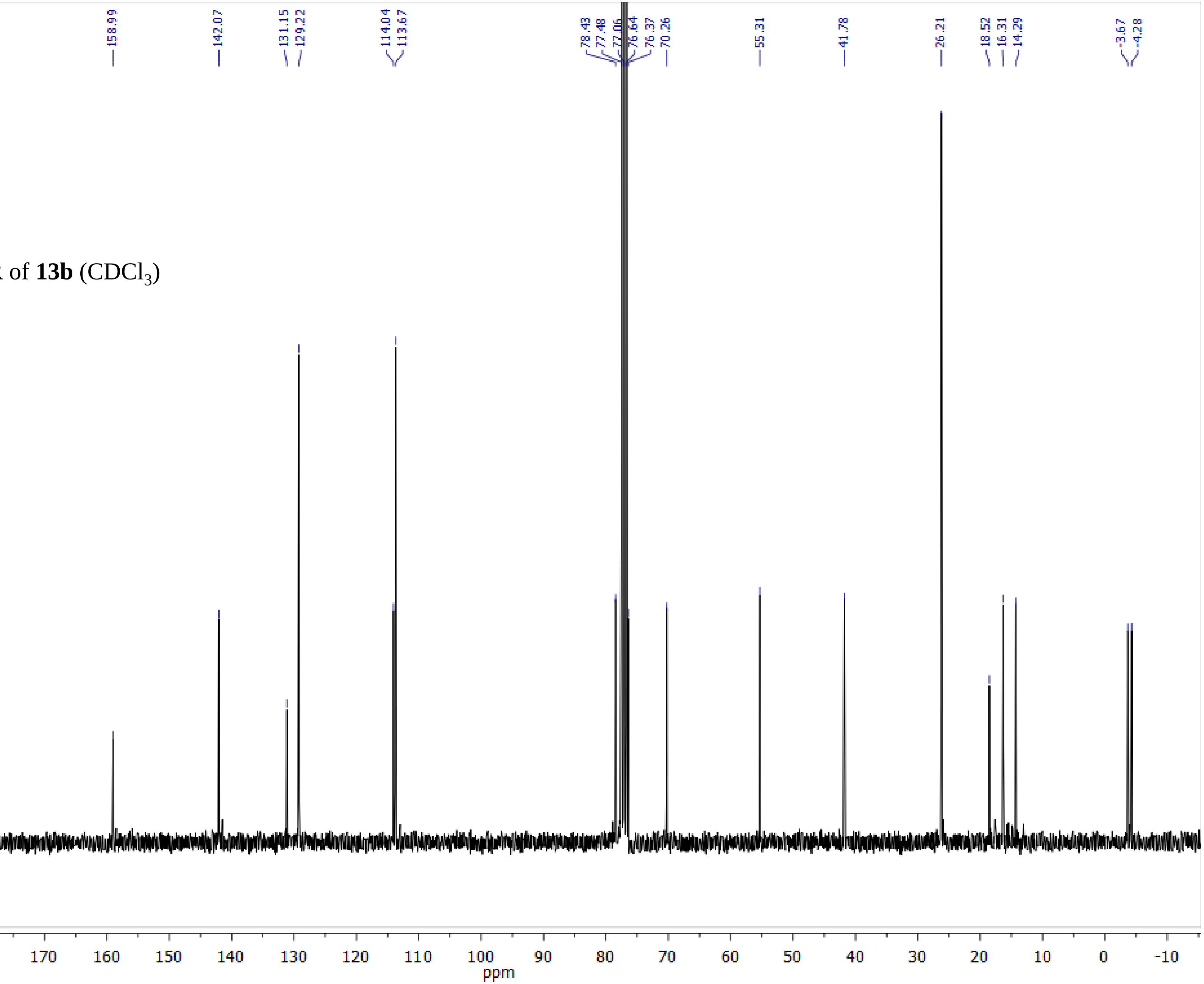

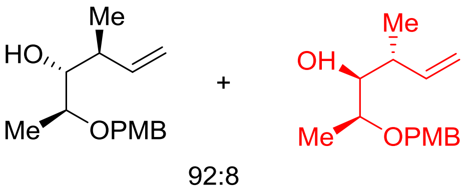

^{1}H NMR of **12b** ($CDCl_3$)
Obtained by diastereoselective allylboration

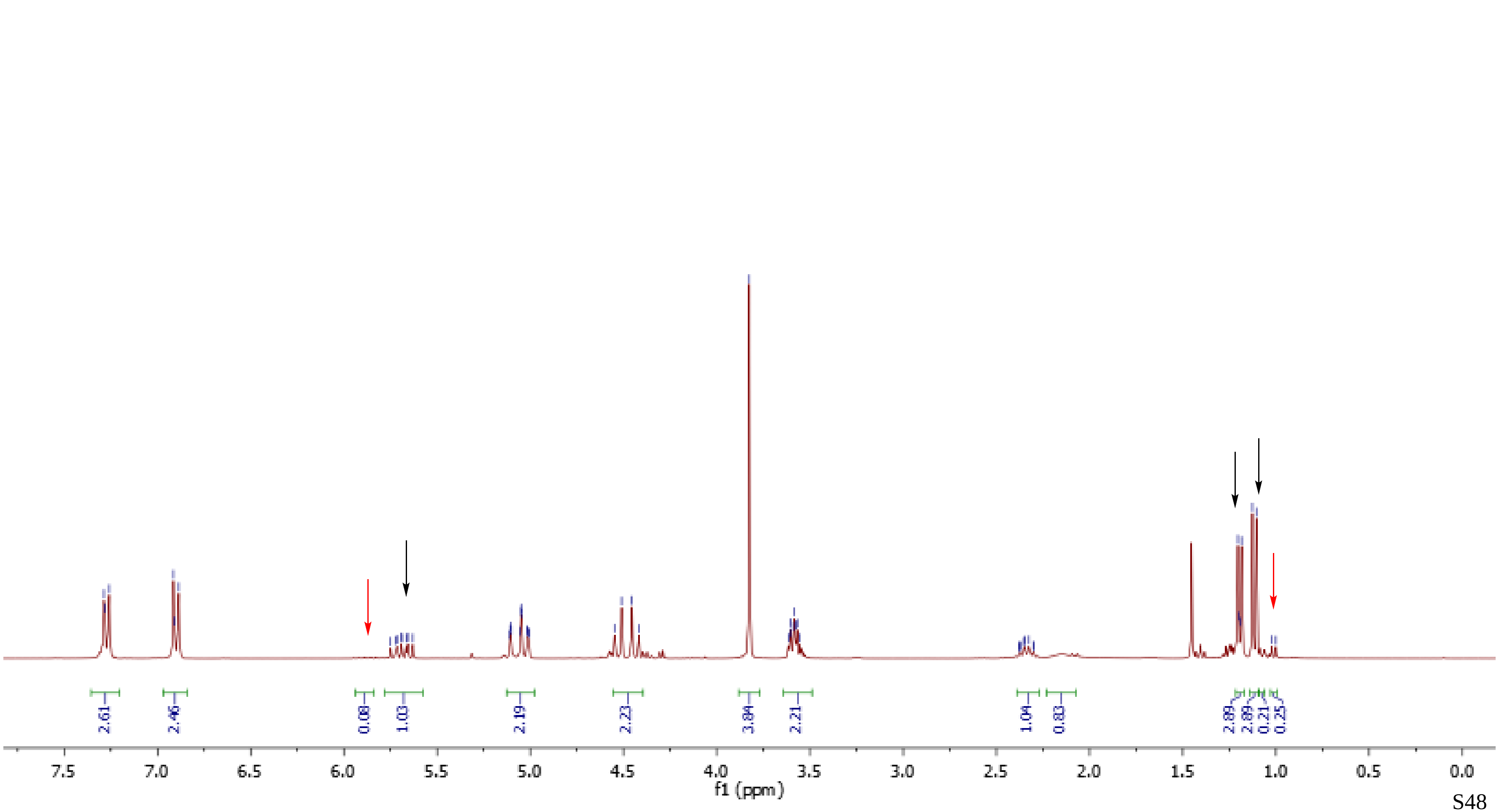

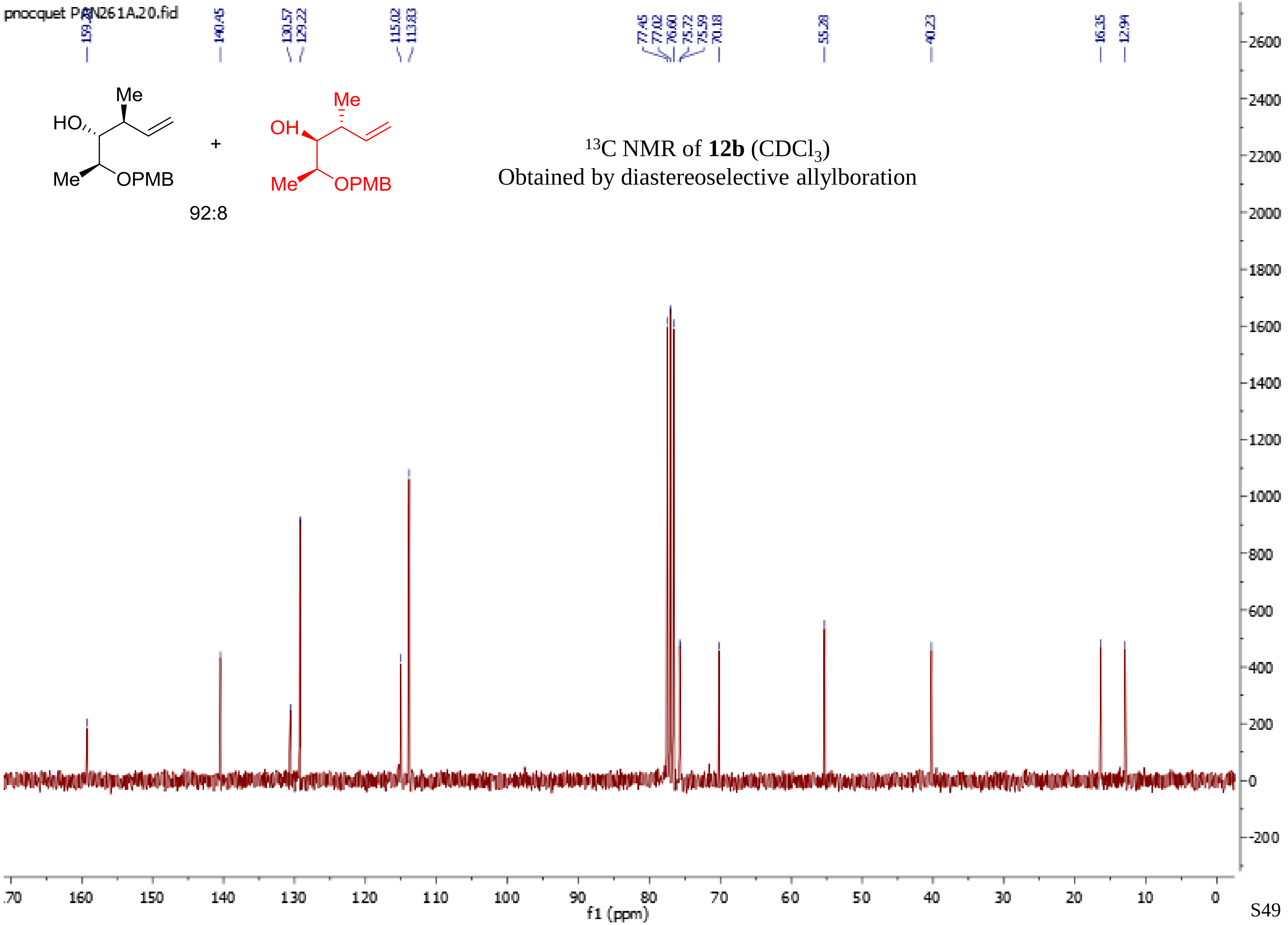

^{13}C NMR of **12b** ($CDCl_3$)
Obtained by diastereoselective allylboration

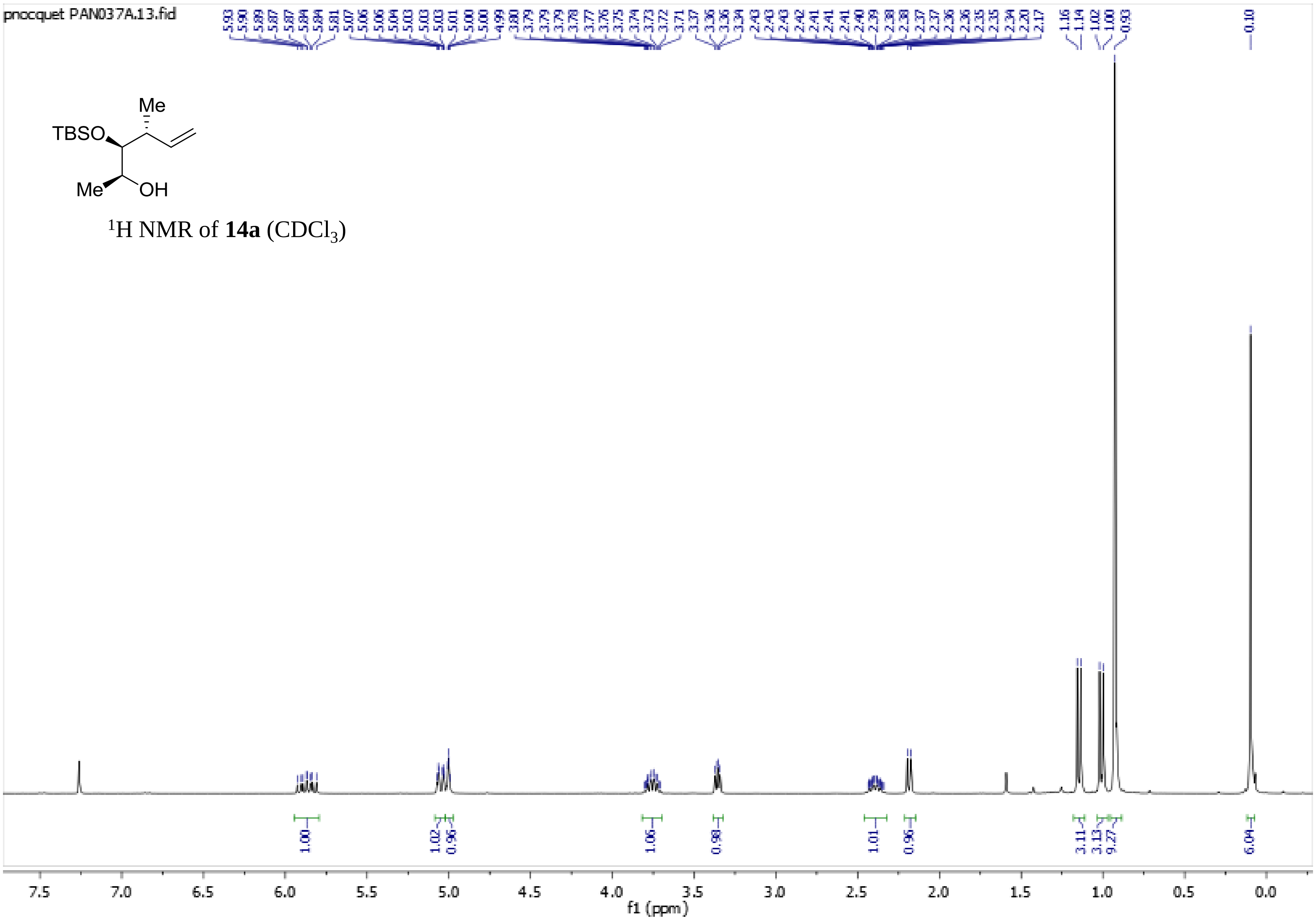

^{1}H NMR of **14a** ($CDCl_3$)

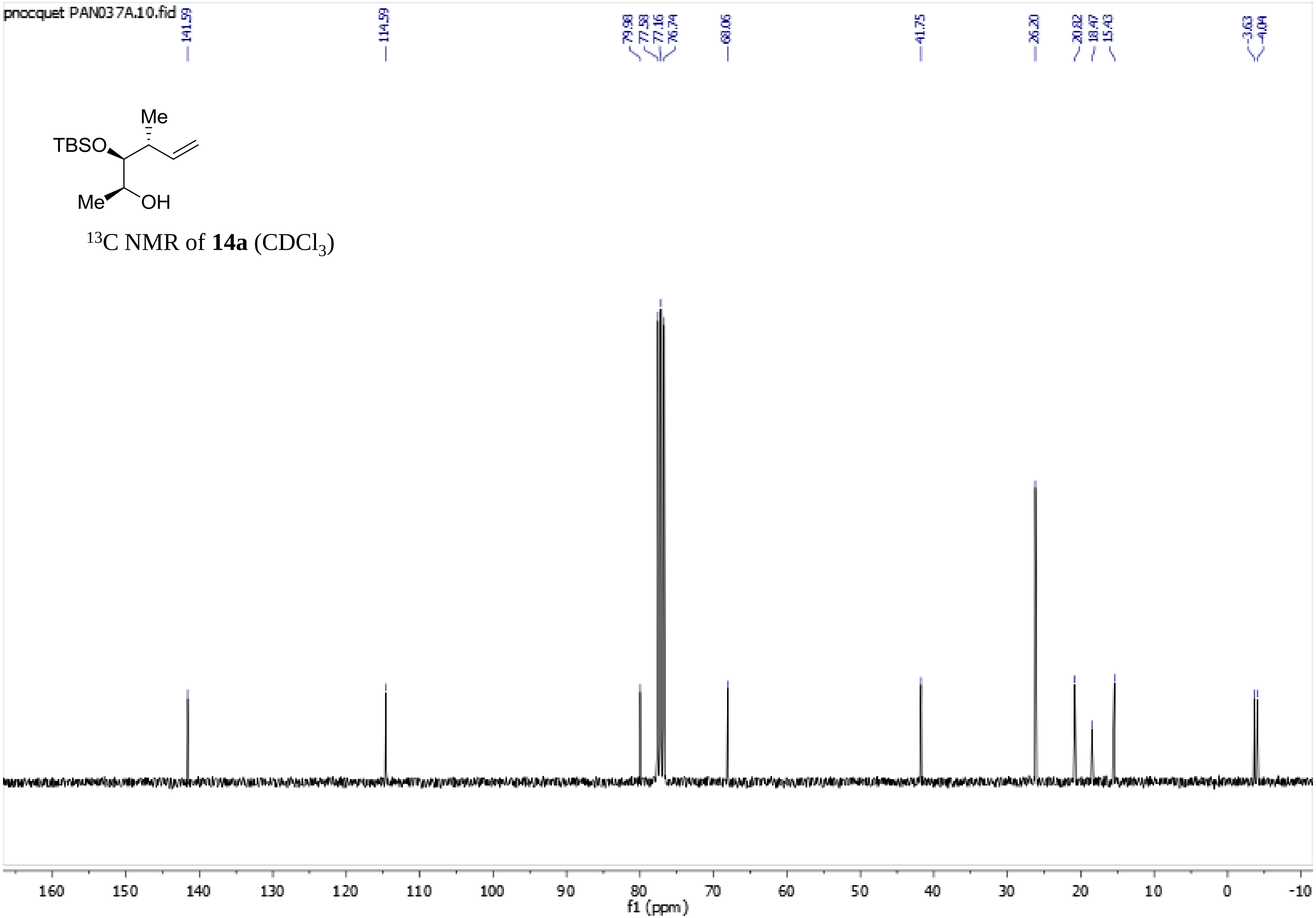

^{13}C NMR of **14a** ($CDCl_3$)

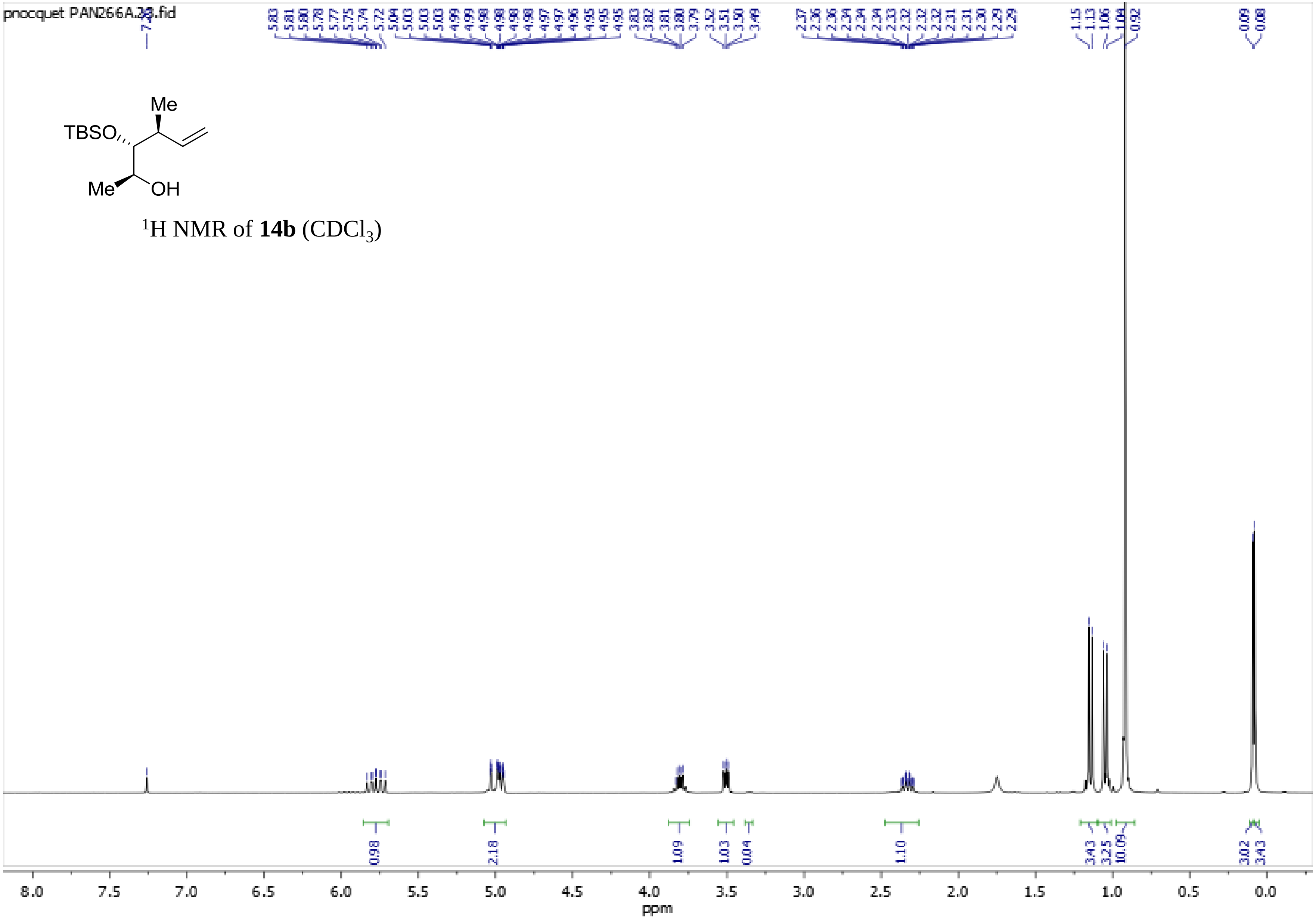

^{1}H NMR of **14b** ($CDCl_3$)

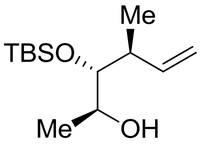

^{13}C NMR of **14b** ($CDCl_3$)

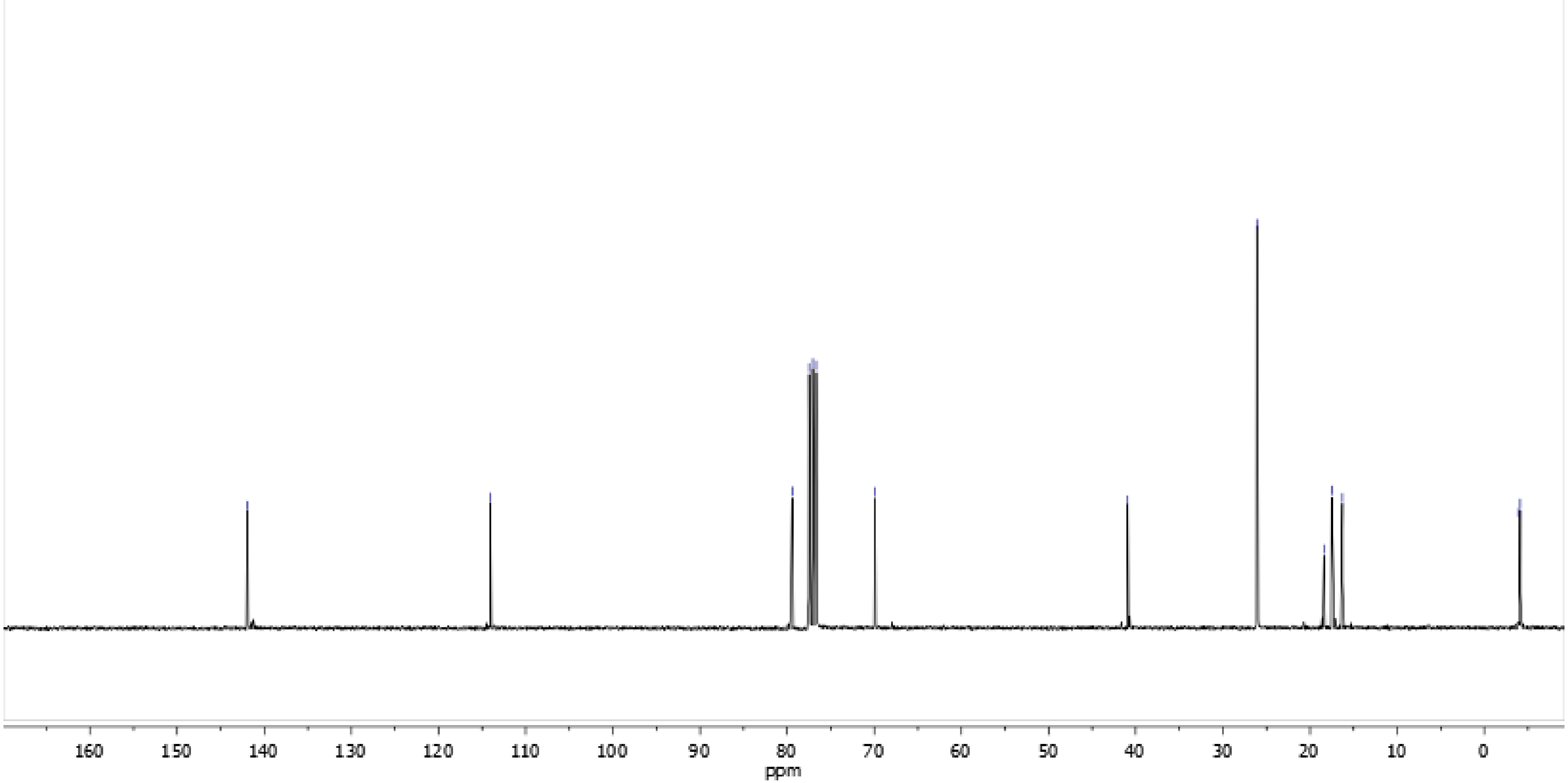

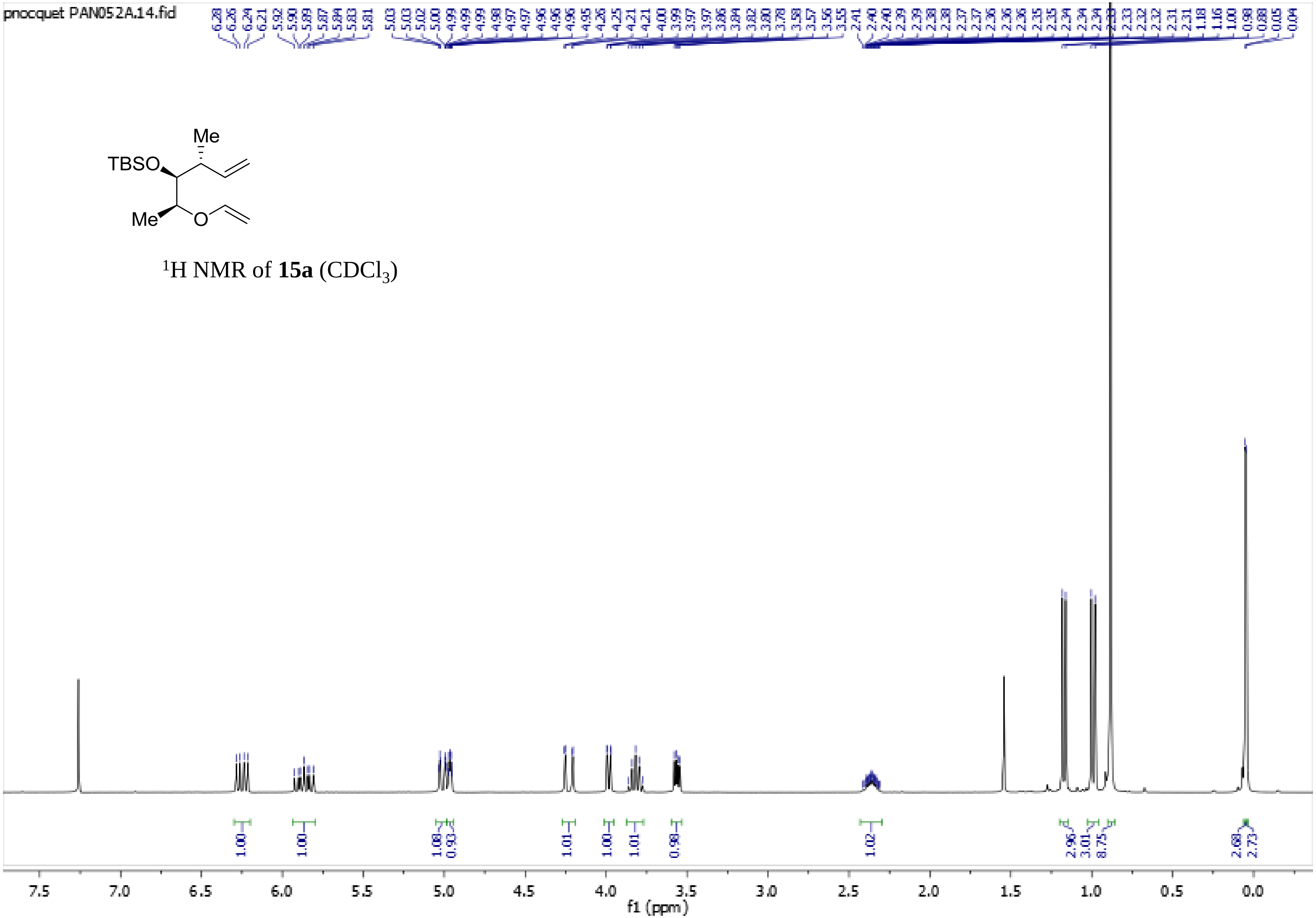

^{1}H NMR of **15a** ($CDCl_3$)

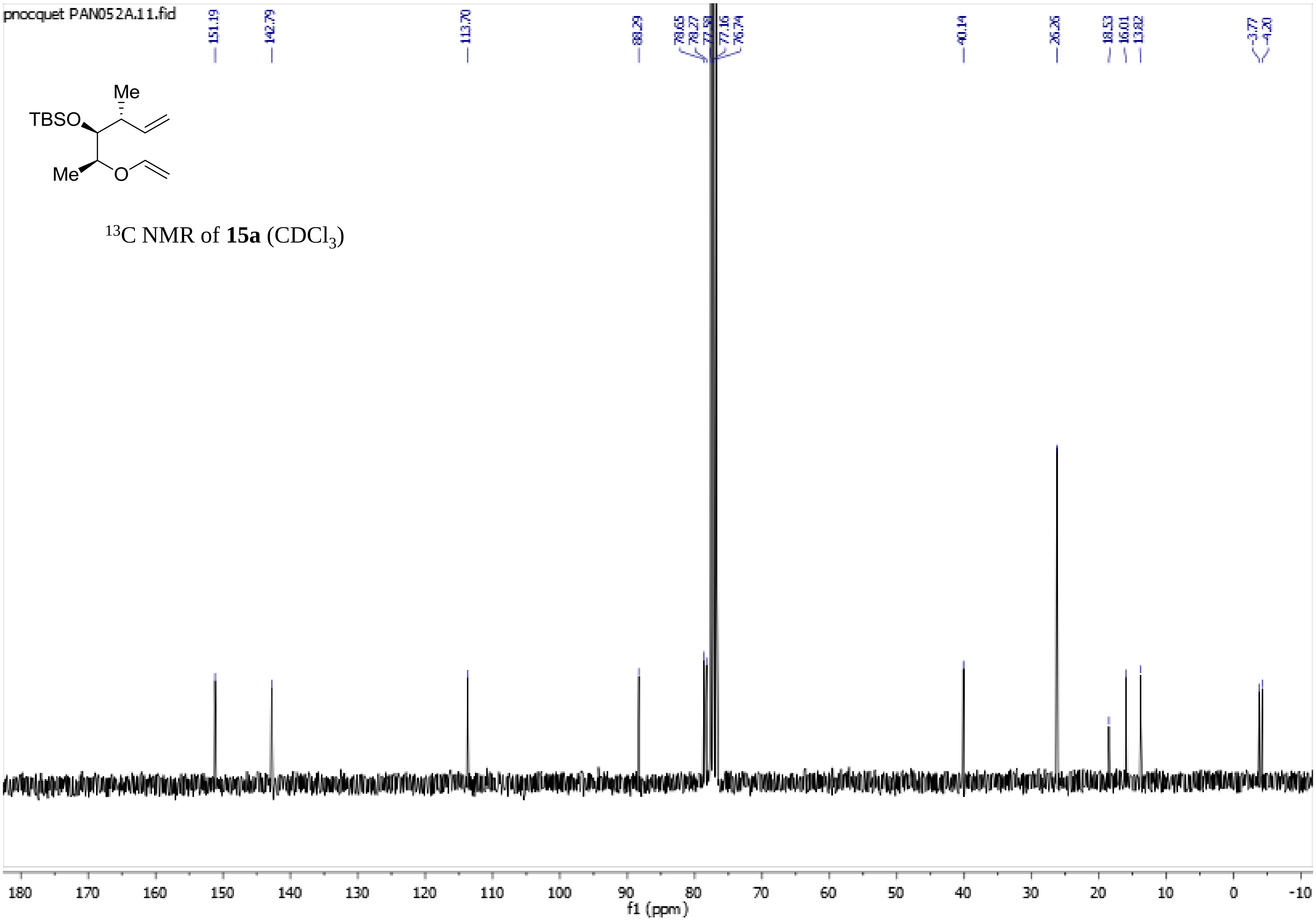

^{13}C NMR of **15a** ($CDCl_3$)

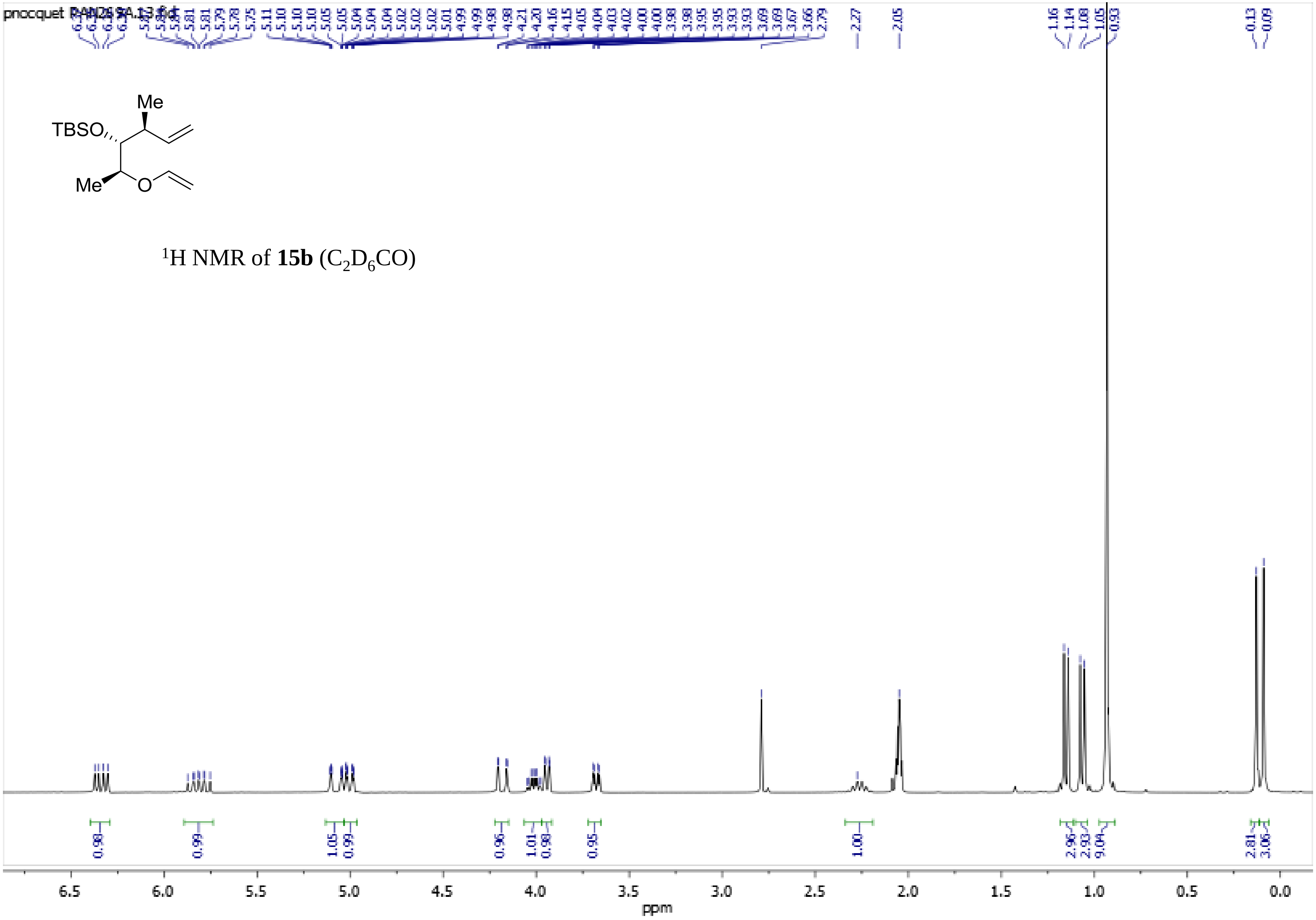

^{1}H NMR of **15b** (C_2D_6CO)

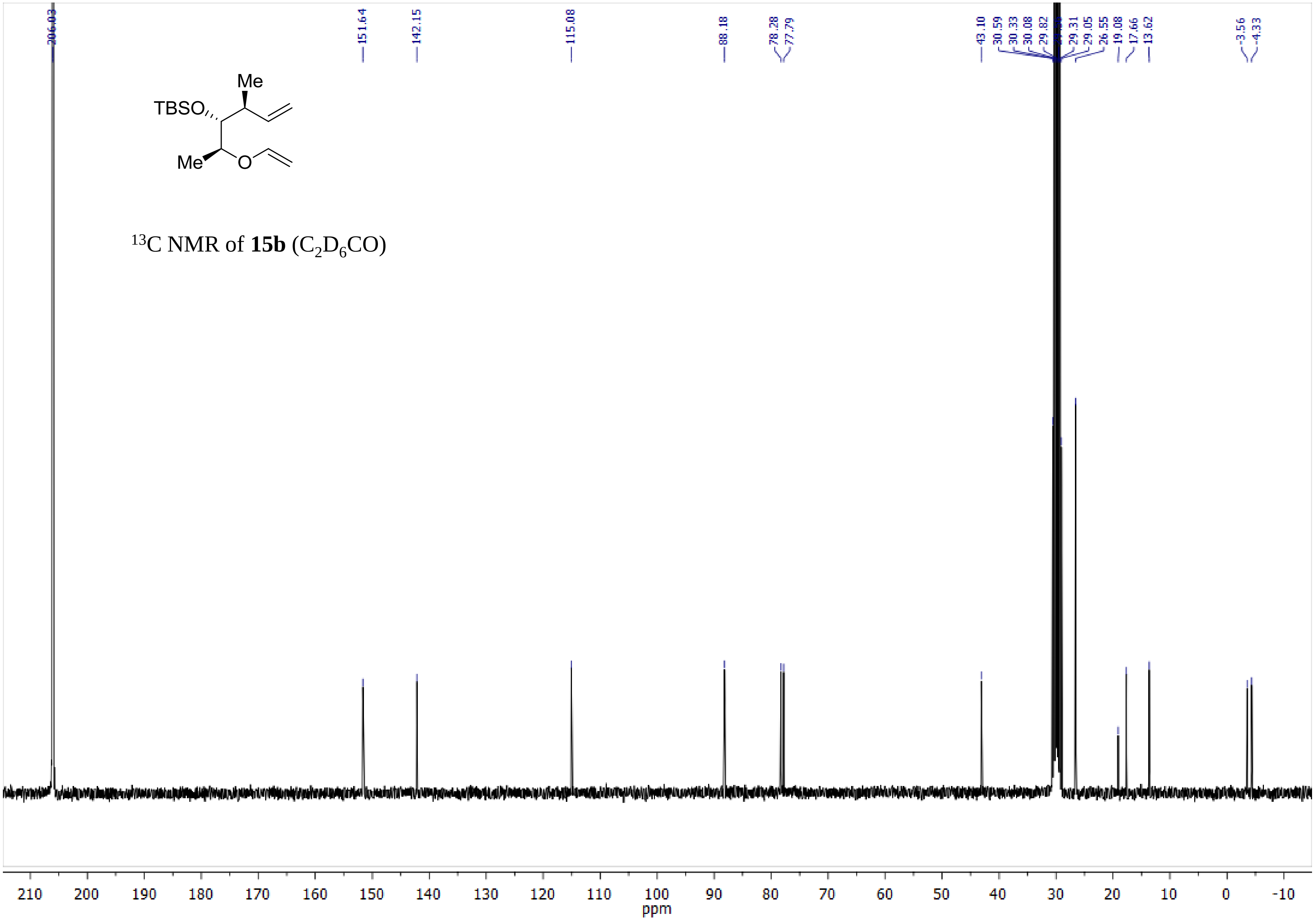

^{13}C NMR of **15b** (C_2D_6CO)

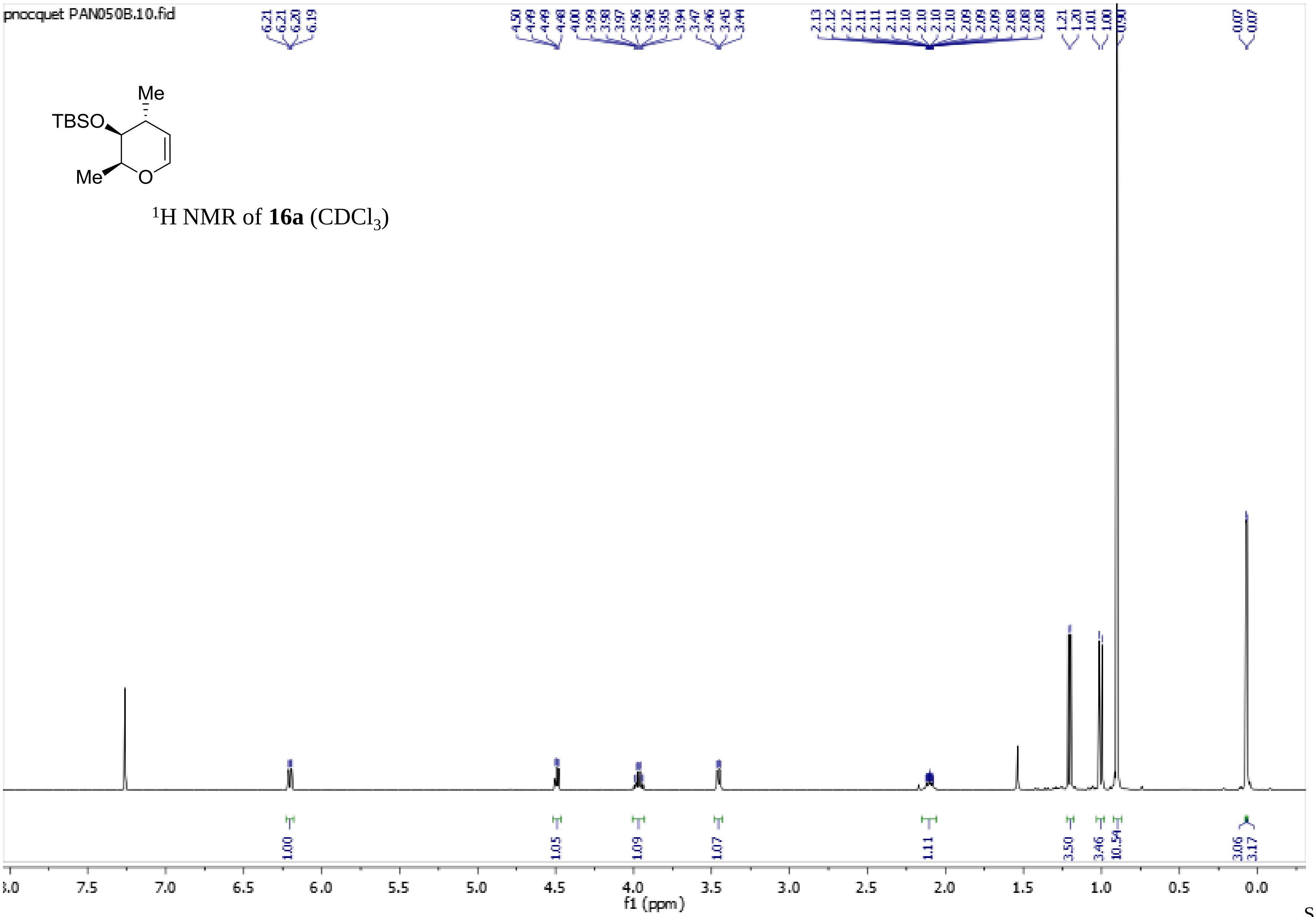

^1H NMR of **16a** ($CDCl_3$)

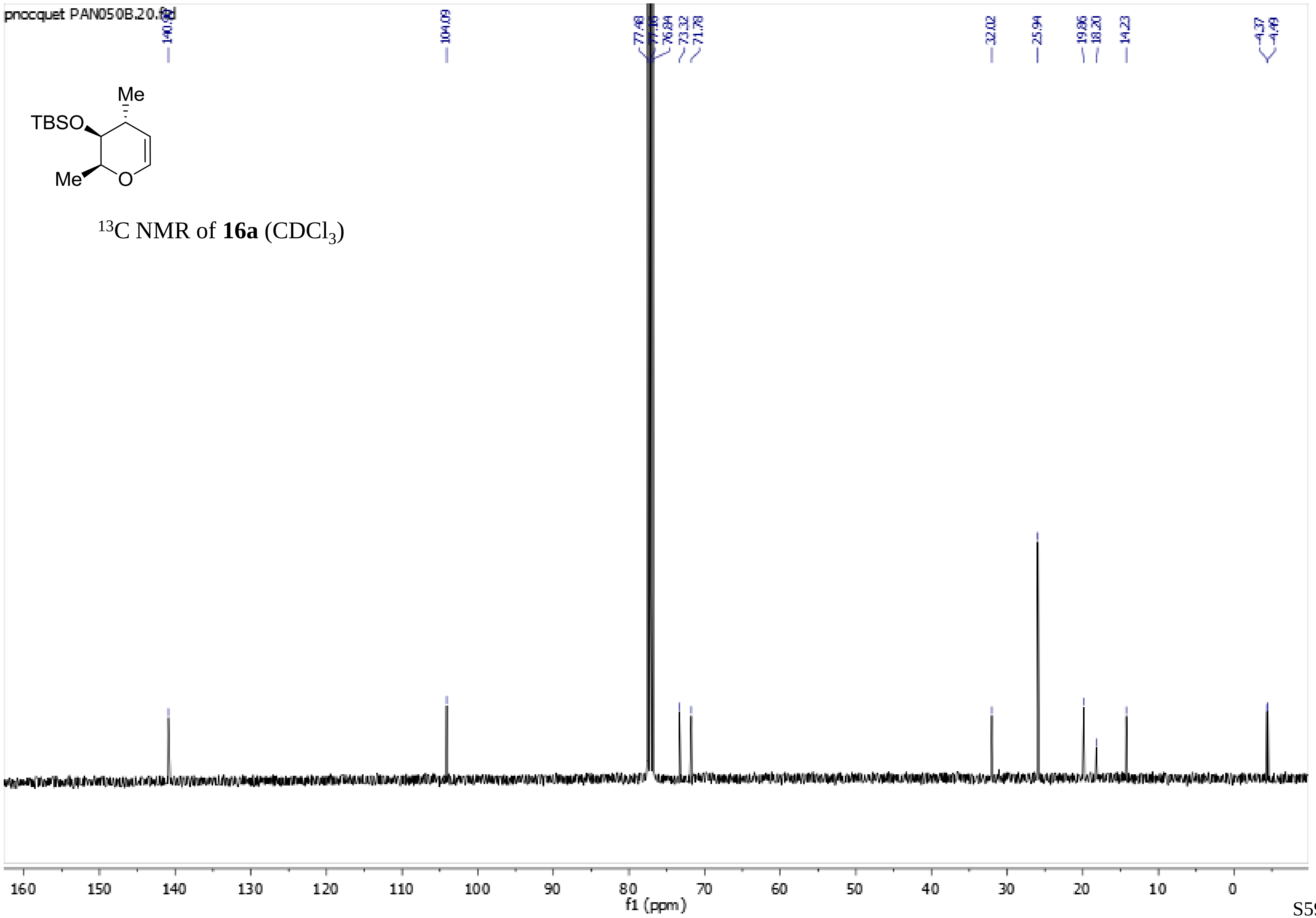

^{13}C NMR of **16a** ($CDCl_3$)

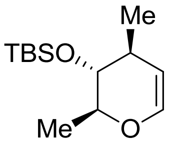

^{1}H NMR of **16b** (C_2D_6CO)

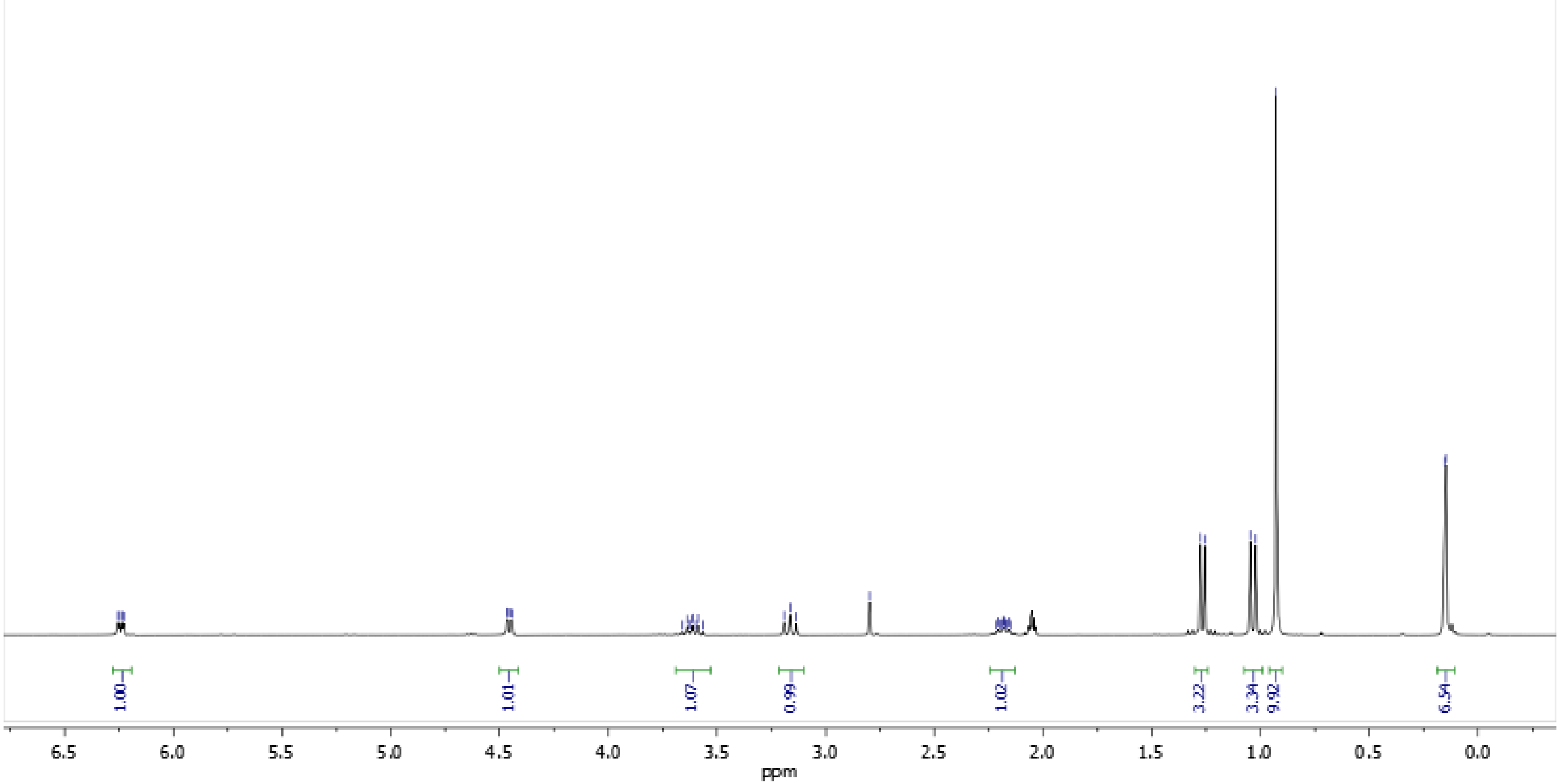

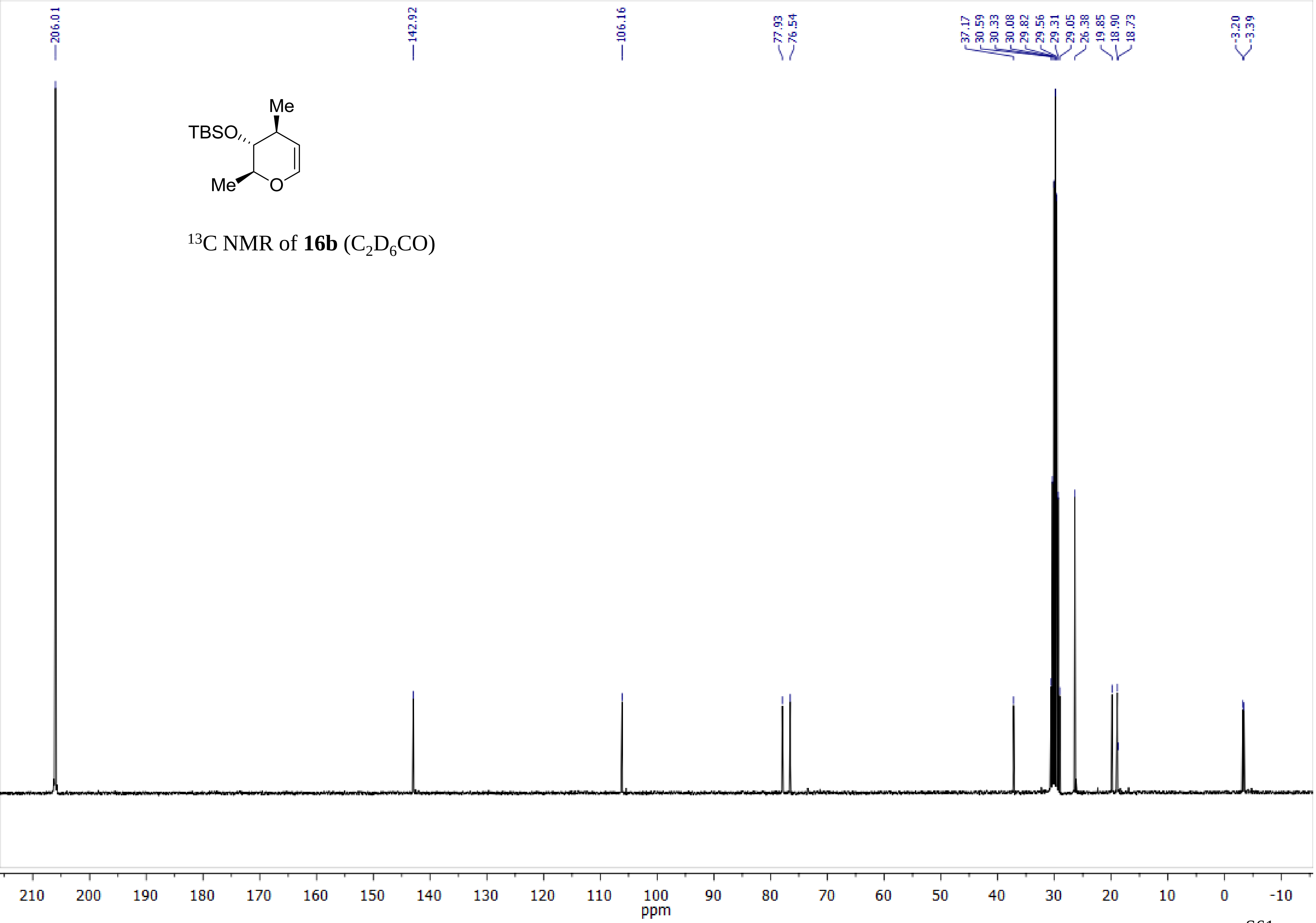

^{13}C NMR of **16b** (C_2D_6CO)

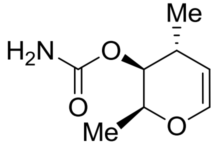

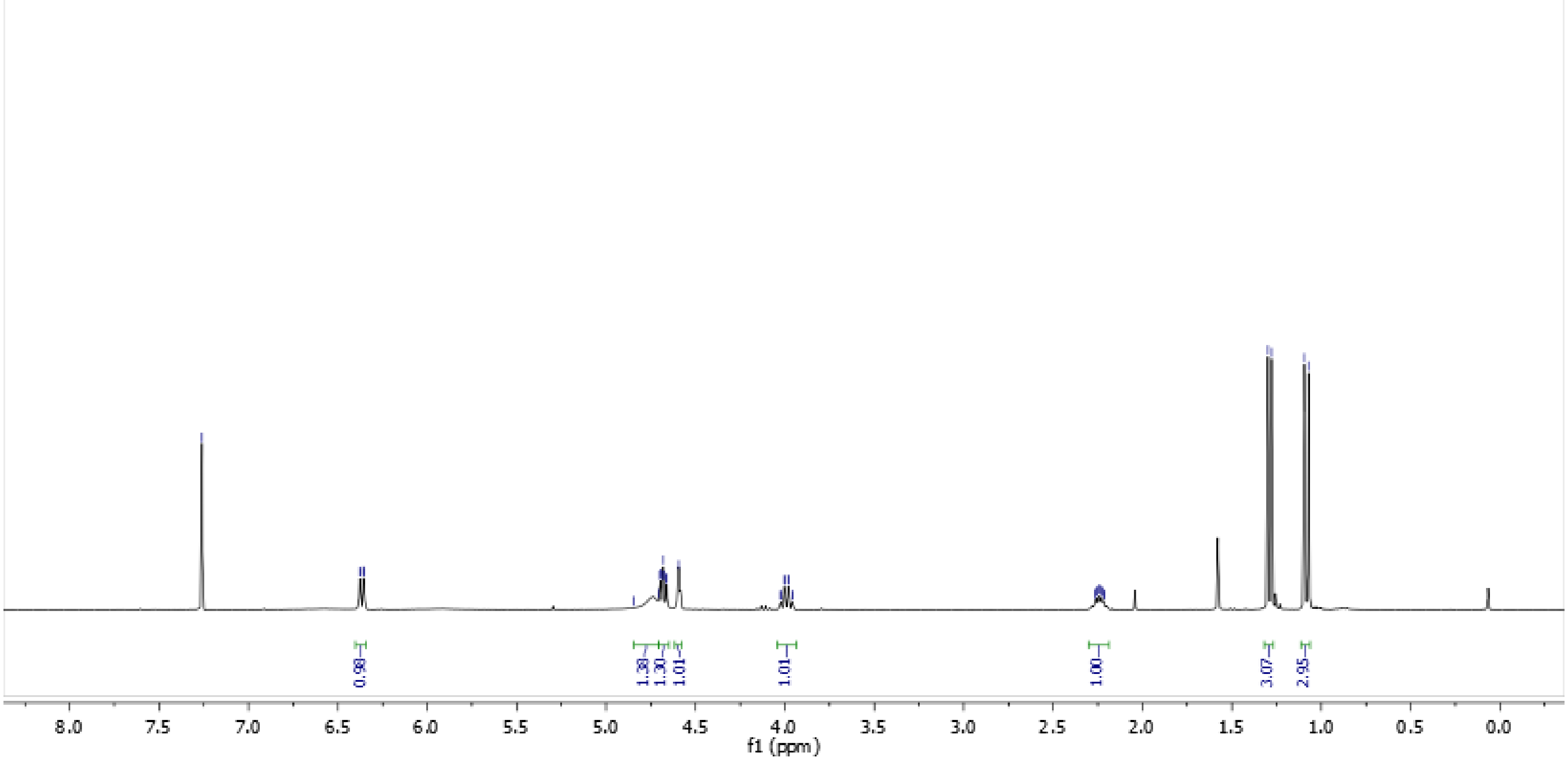

^{1}H NMR of **17a** ($CDCl_3$)

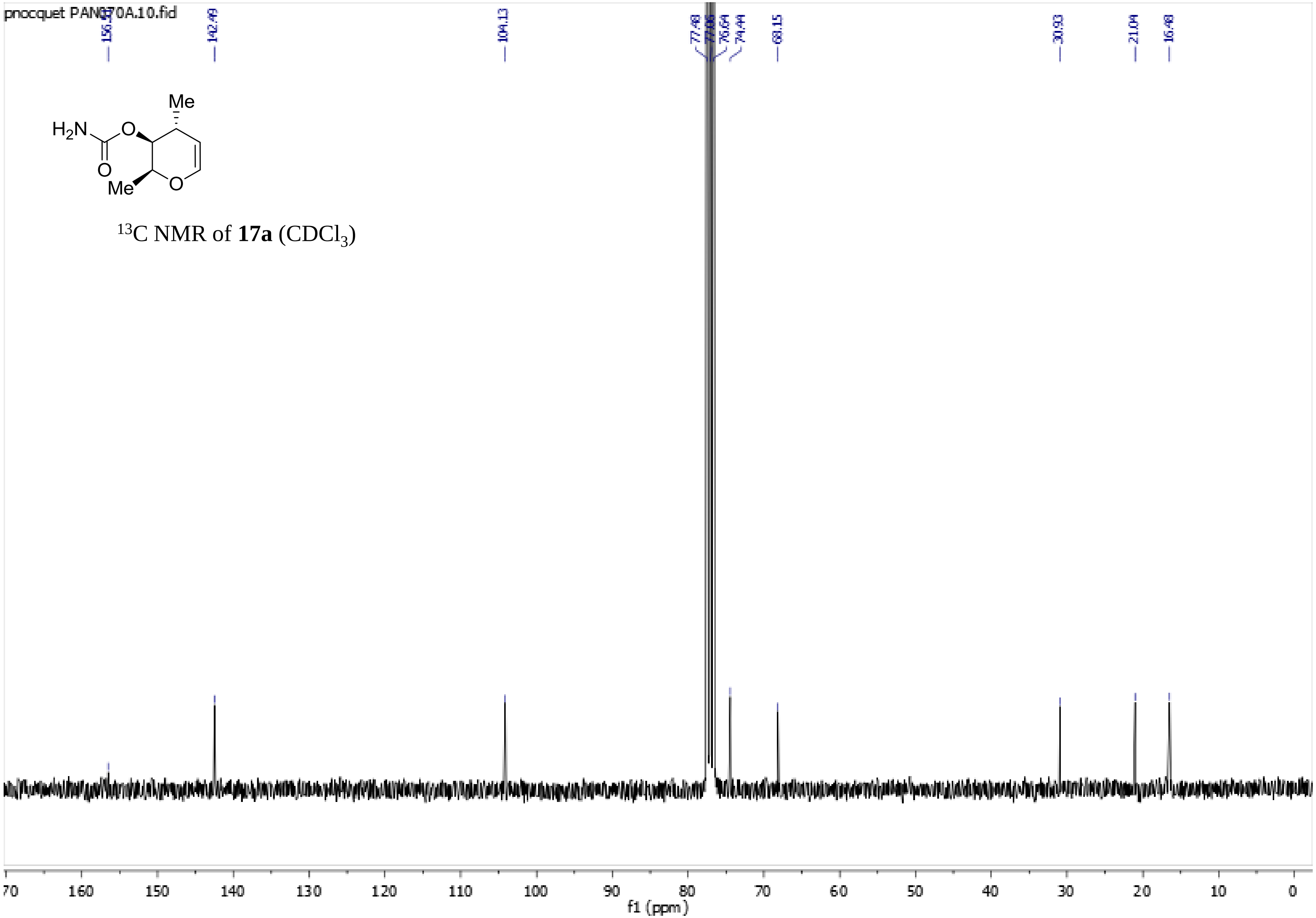

^{13}C NMR of **17a** ($CDCl_3$)

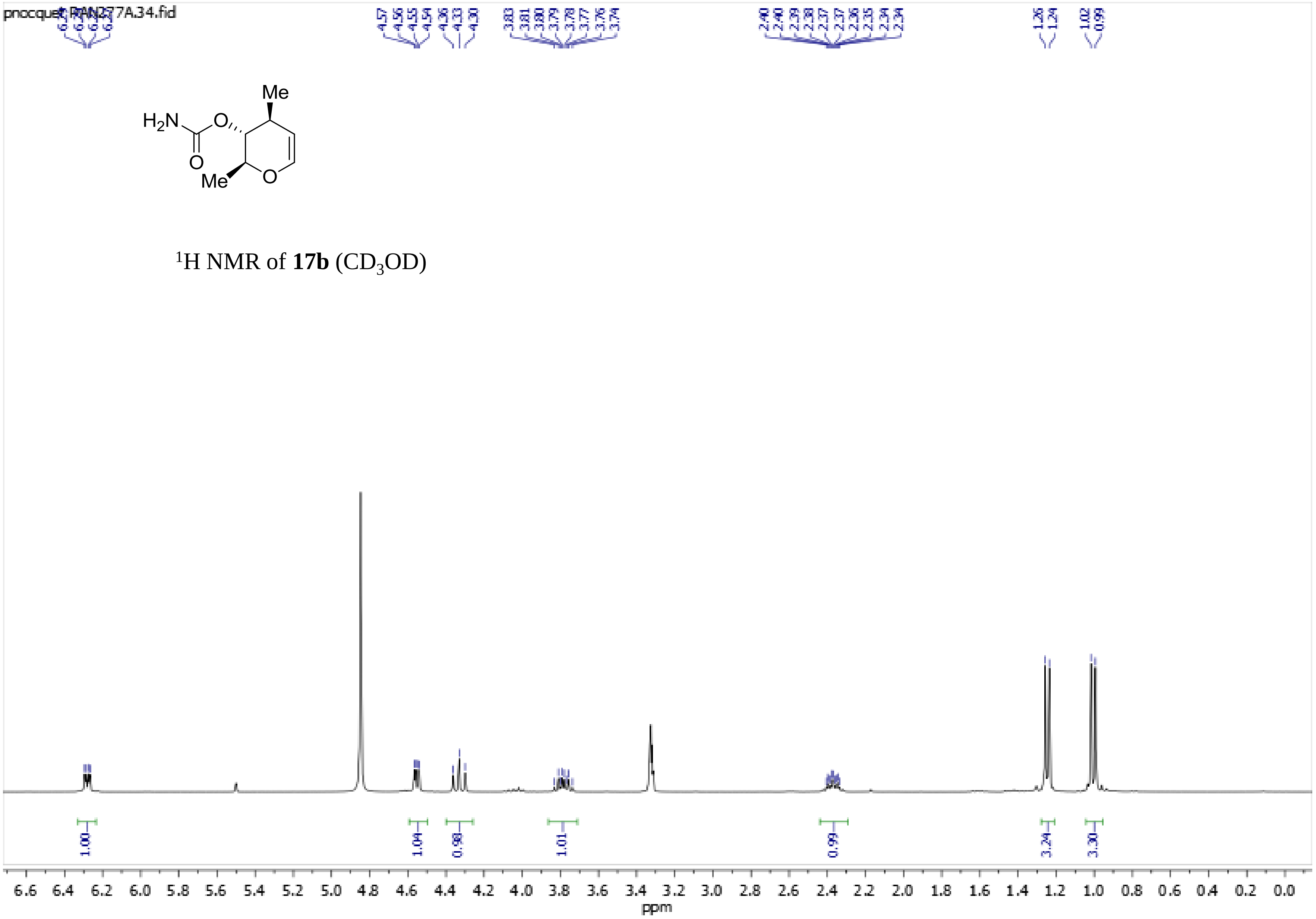

^{1}H NMR of **17b** (CD_3OD)

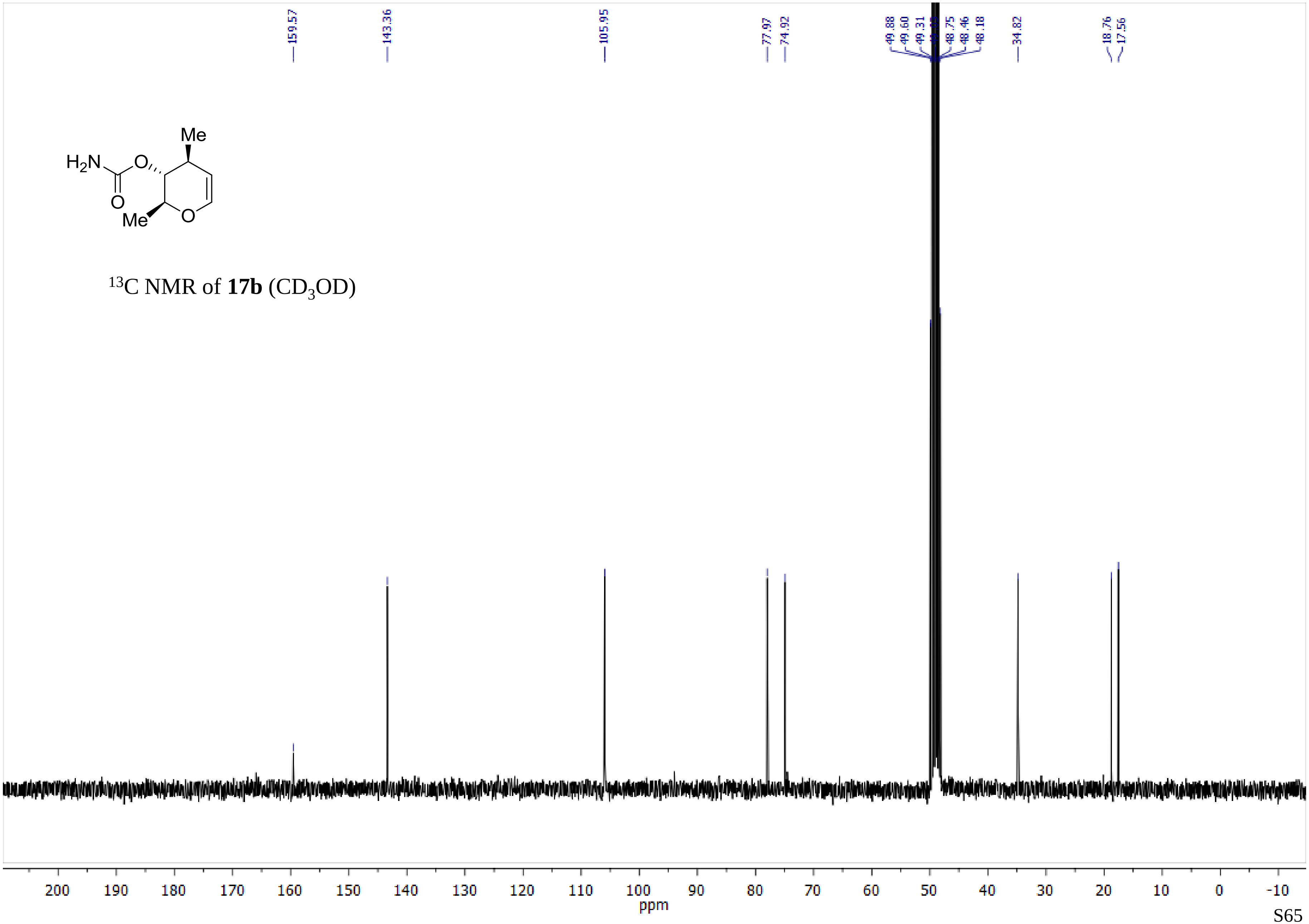

^{13}C NMR of **17b** (CD_3OD)

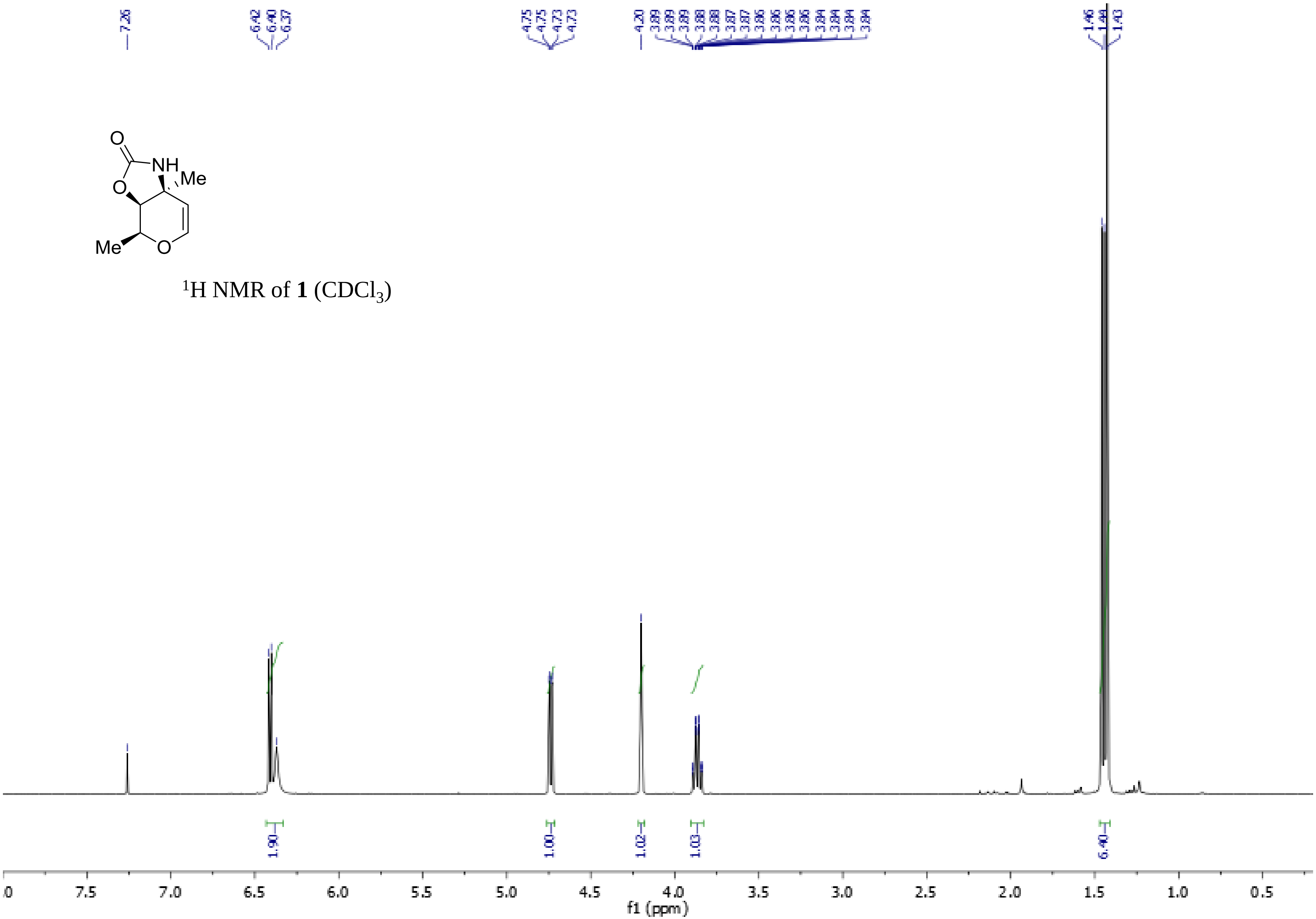

^{1}H NMR of **1** ($CDCl_3$)

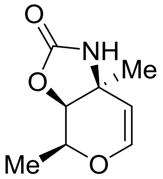

^{13}C NMR of **1** ($CDCl_3$)

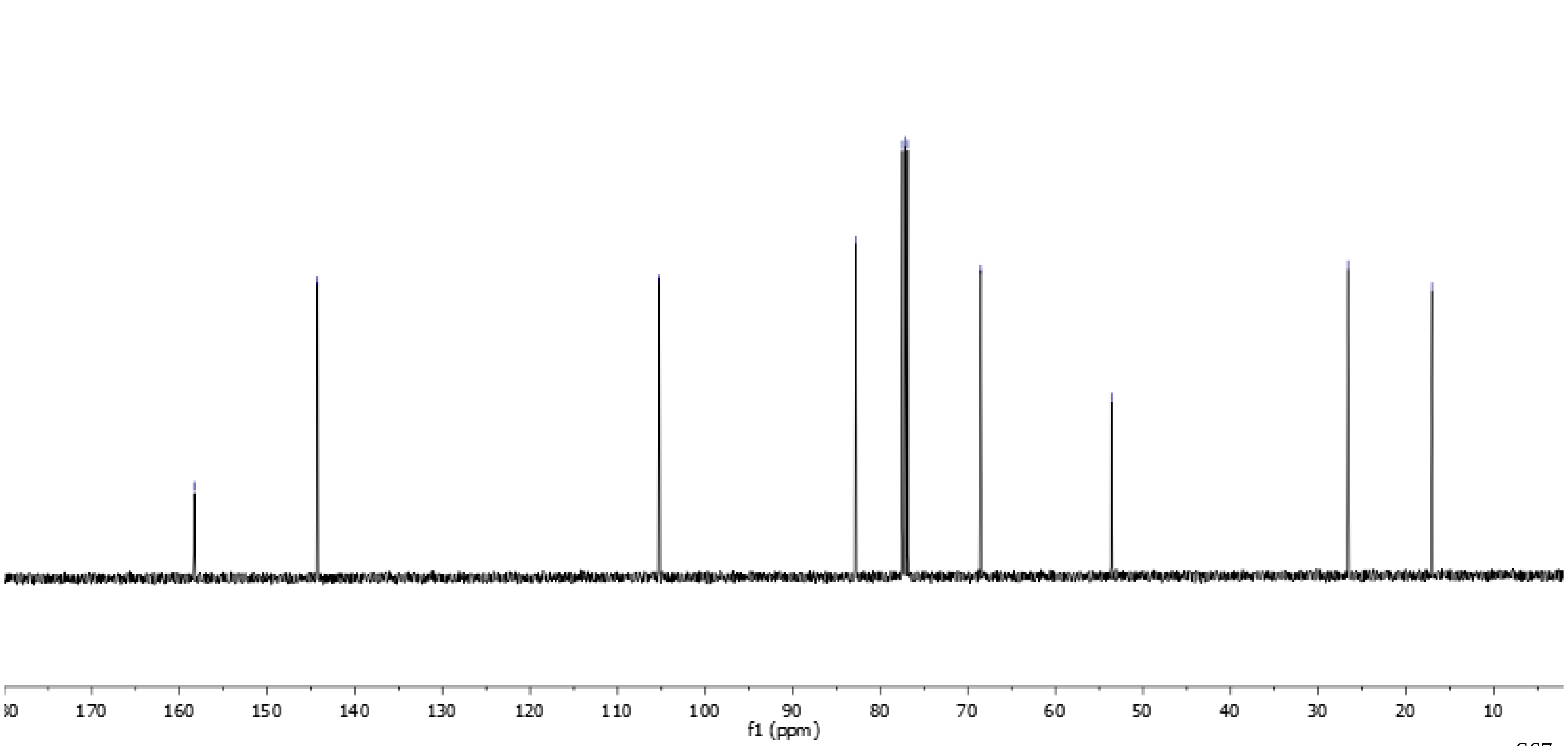

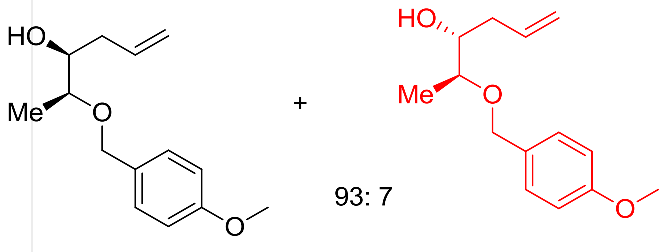

^{1}H NMR of **18** ($CDCl_3$)

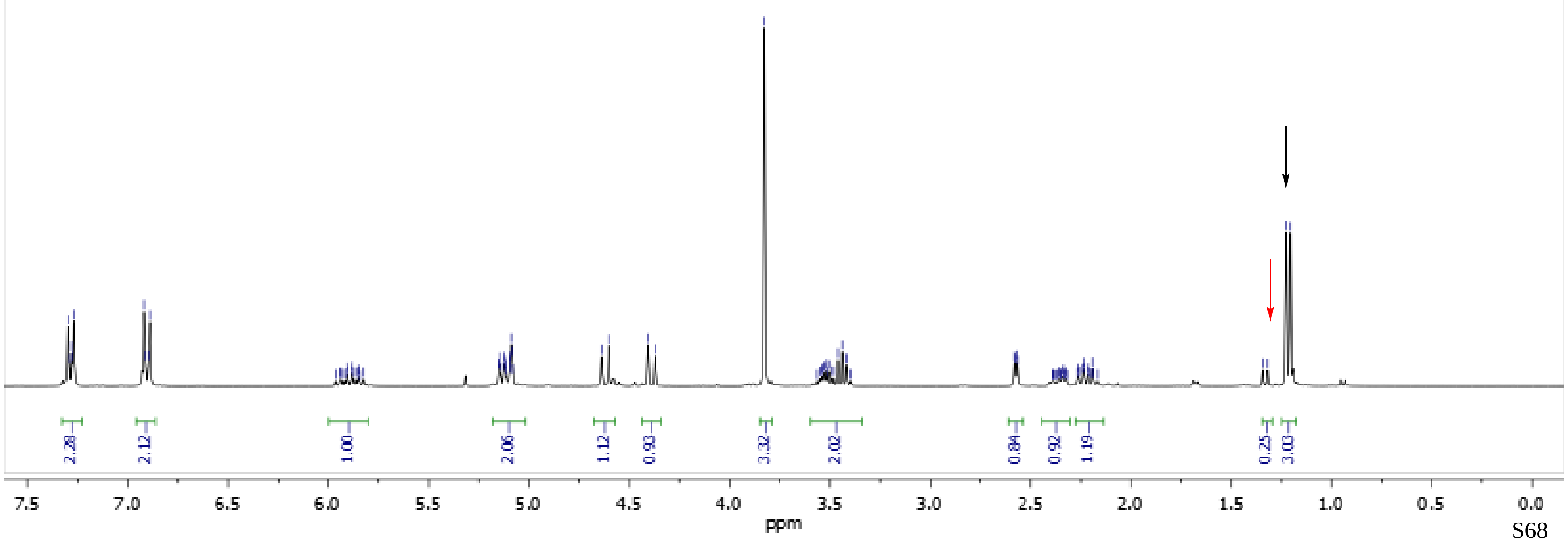

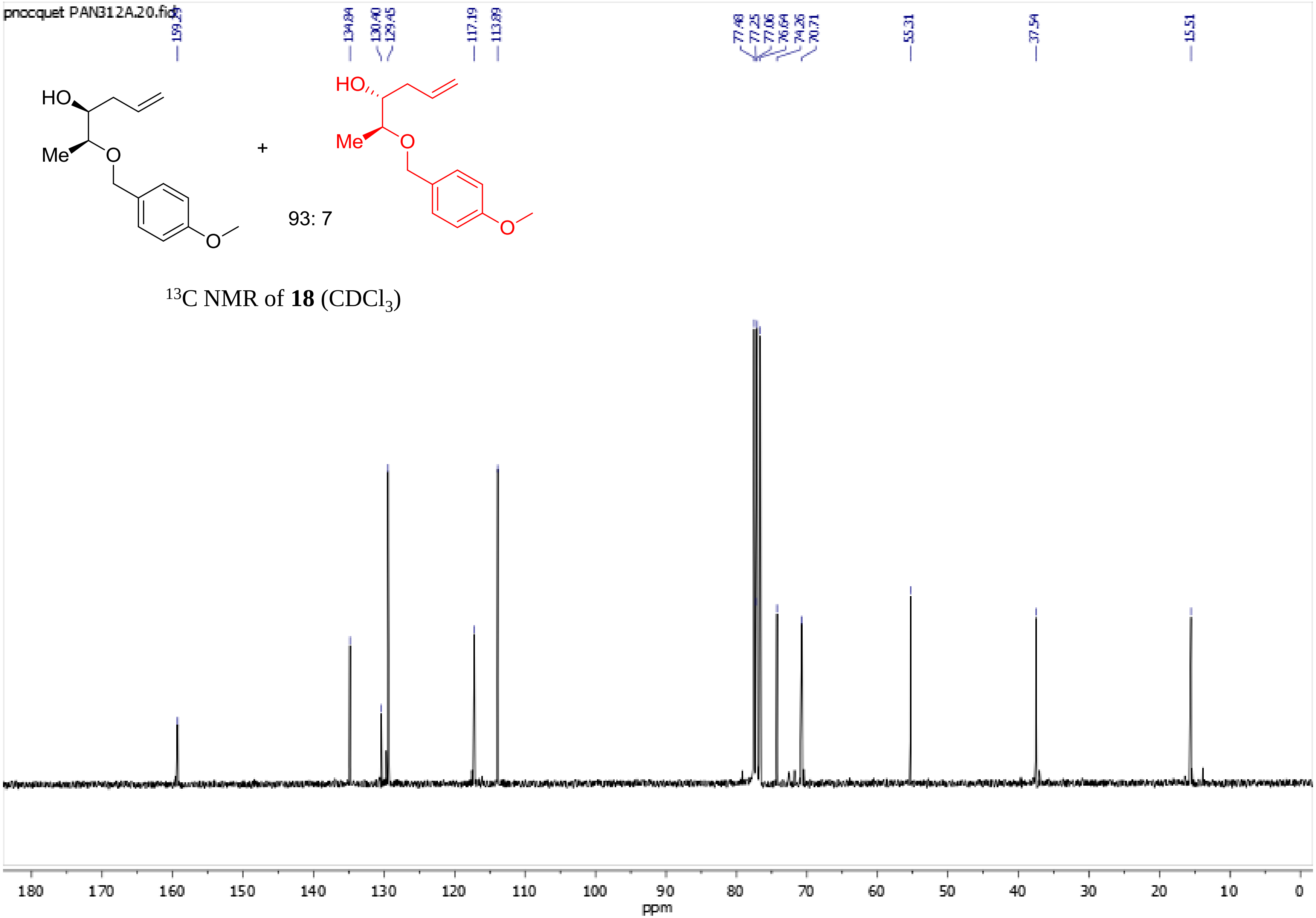

^{13}C NMR of **18** ($CDCl_3$)

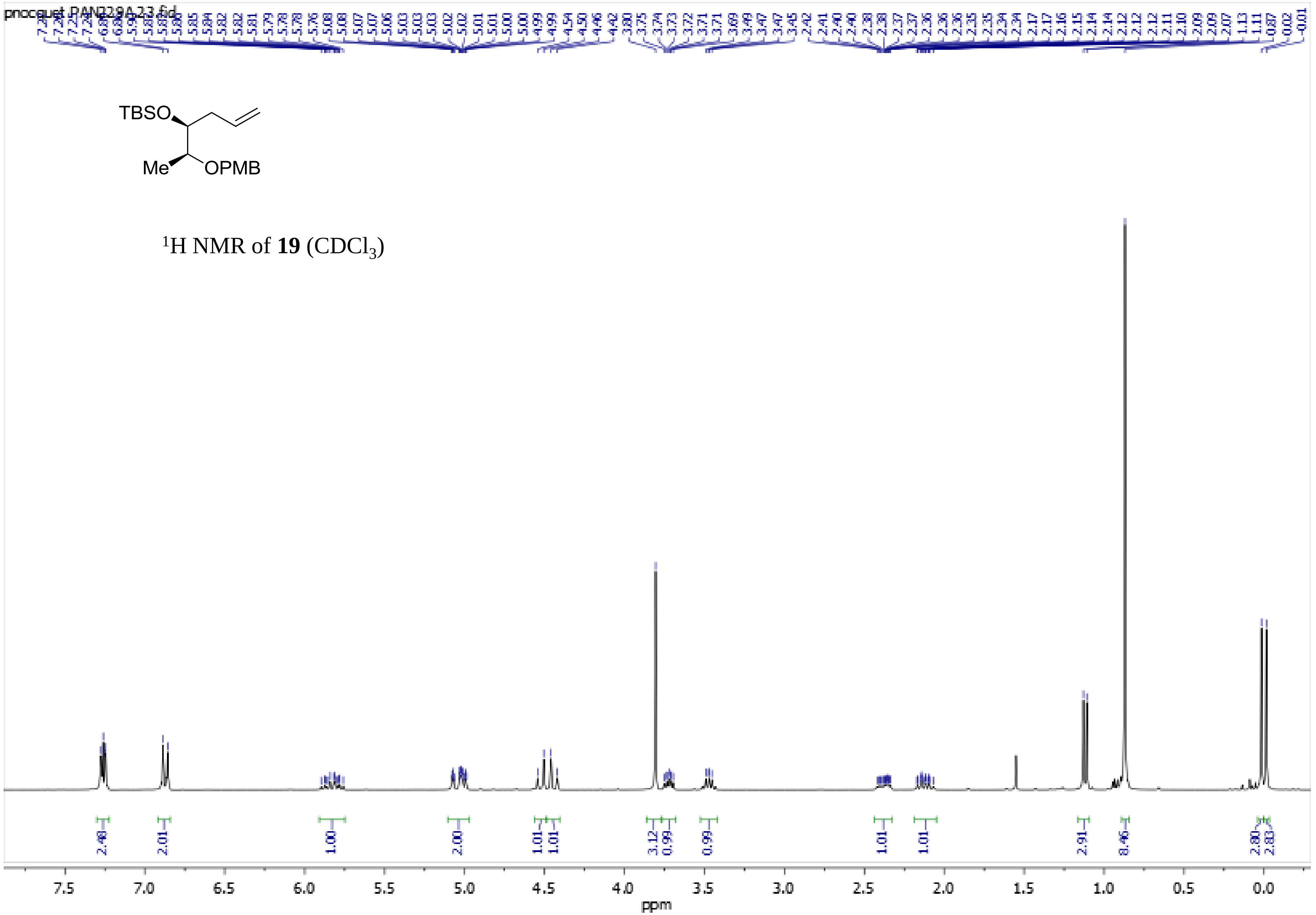

^{1}H NMR of **19** ($CDCl_3$)

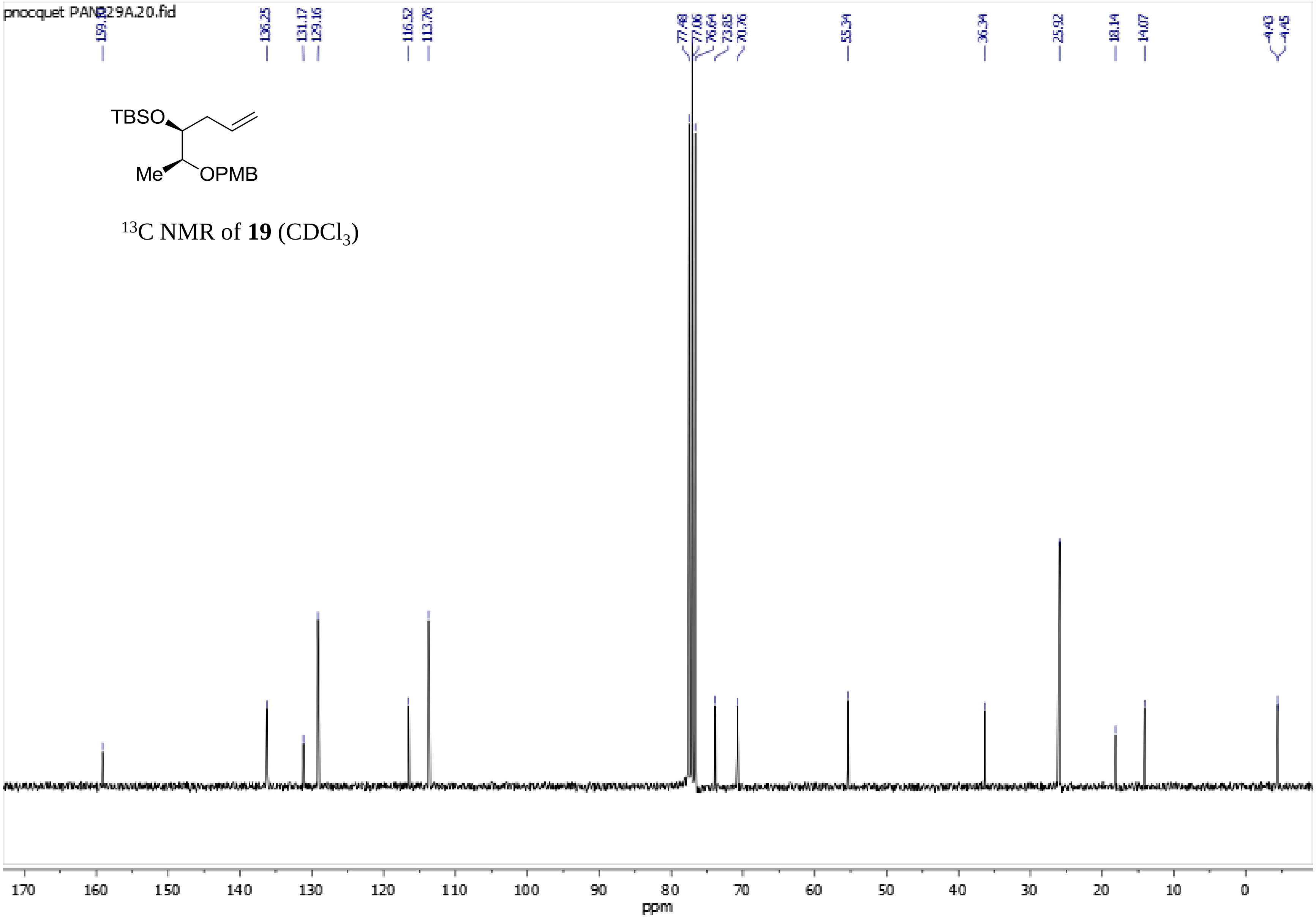

^{13}C NMR of **19** ($CDCl_3$)

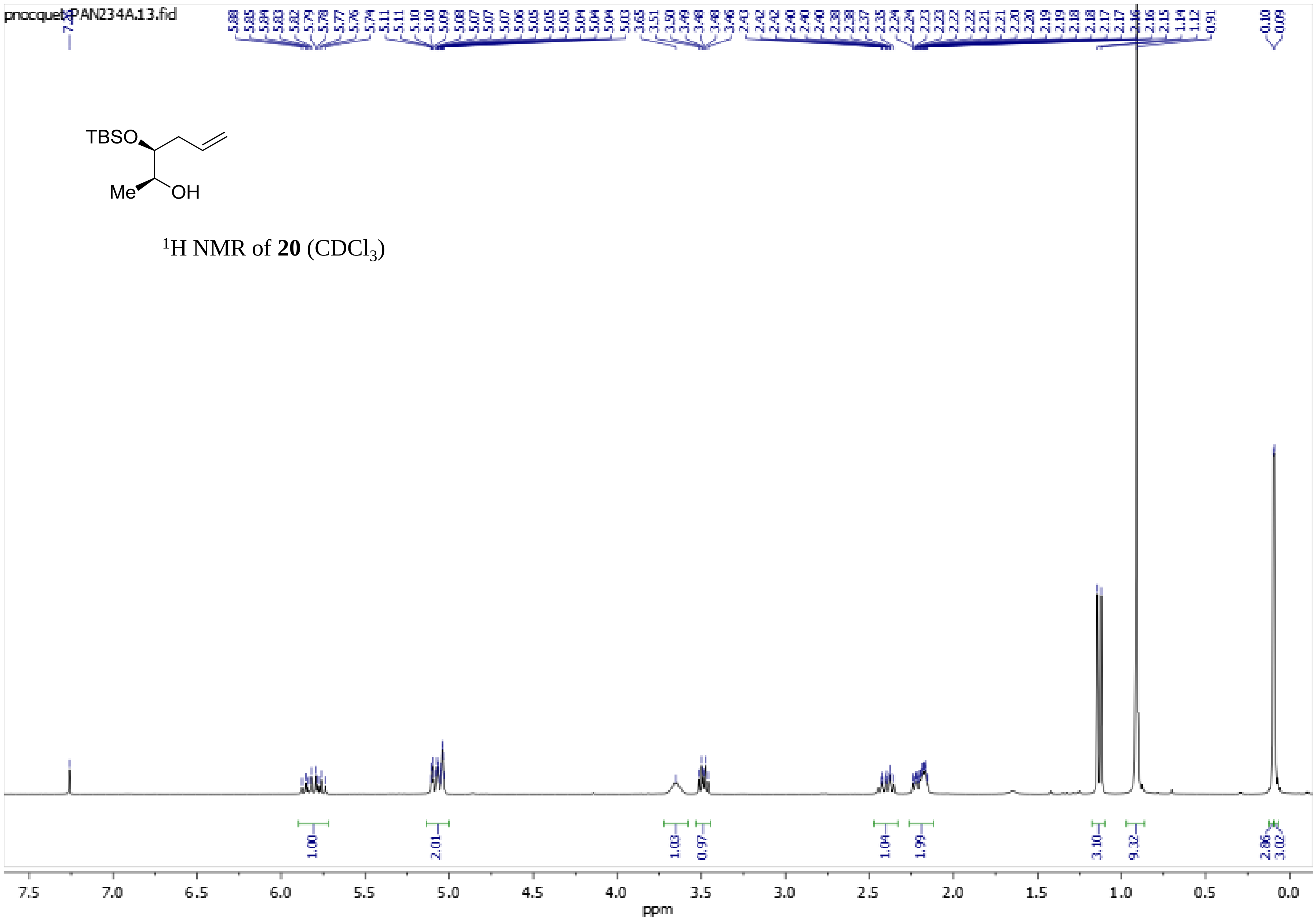

^{1}H NMR of **20** ($CDCl_3$)

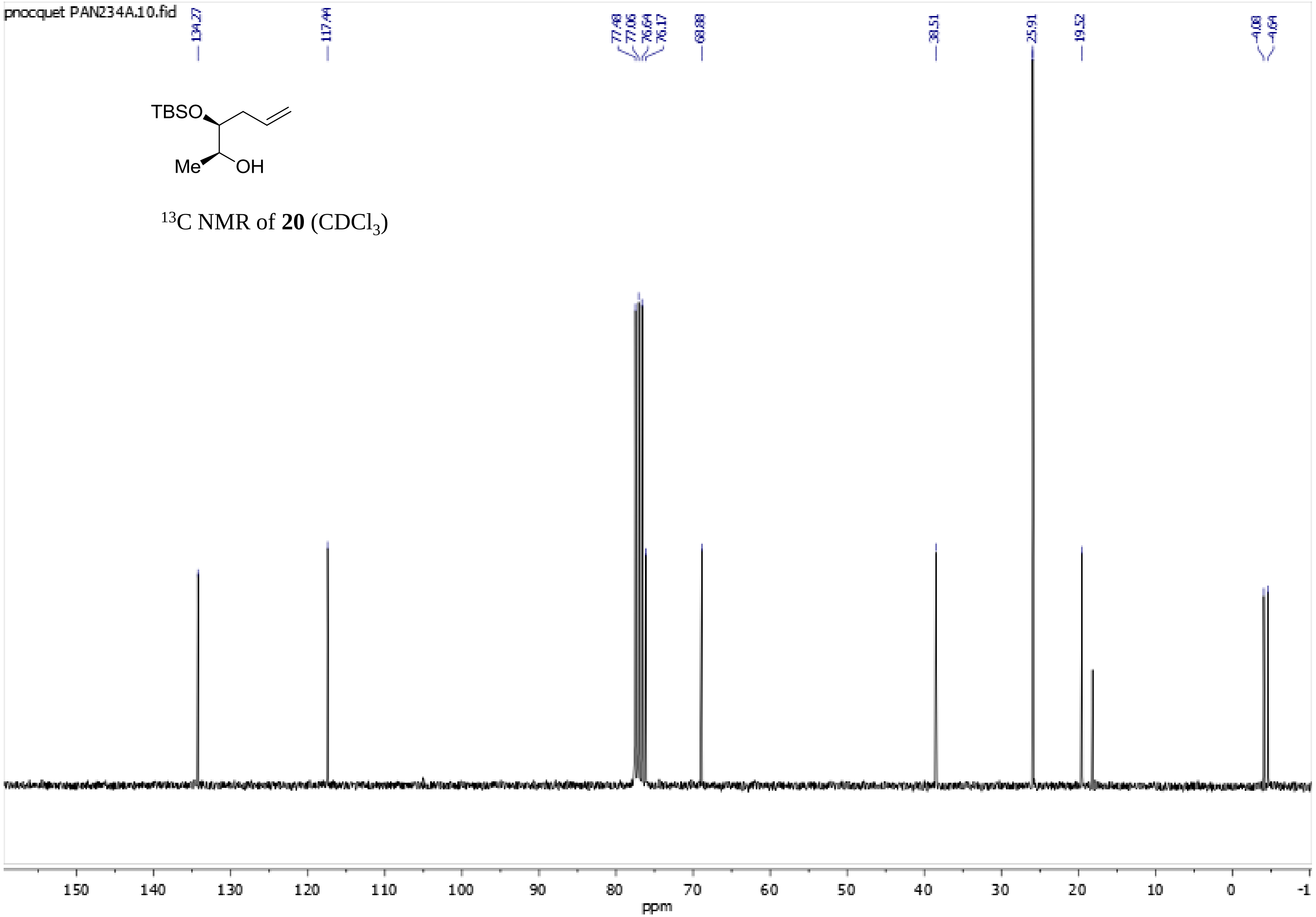

^{13}C NMR of **20** ($CDCl_3$)

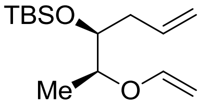

^{1}H NMR of **21** ($CDCl_3$)

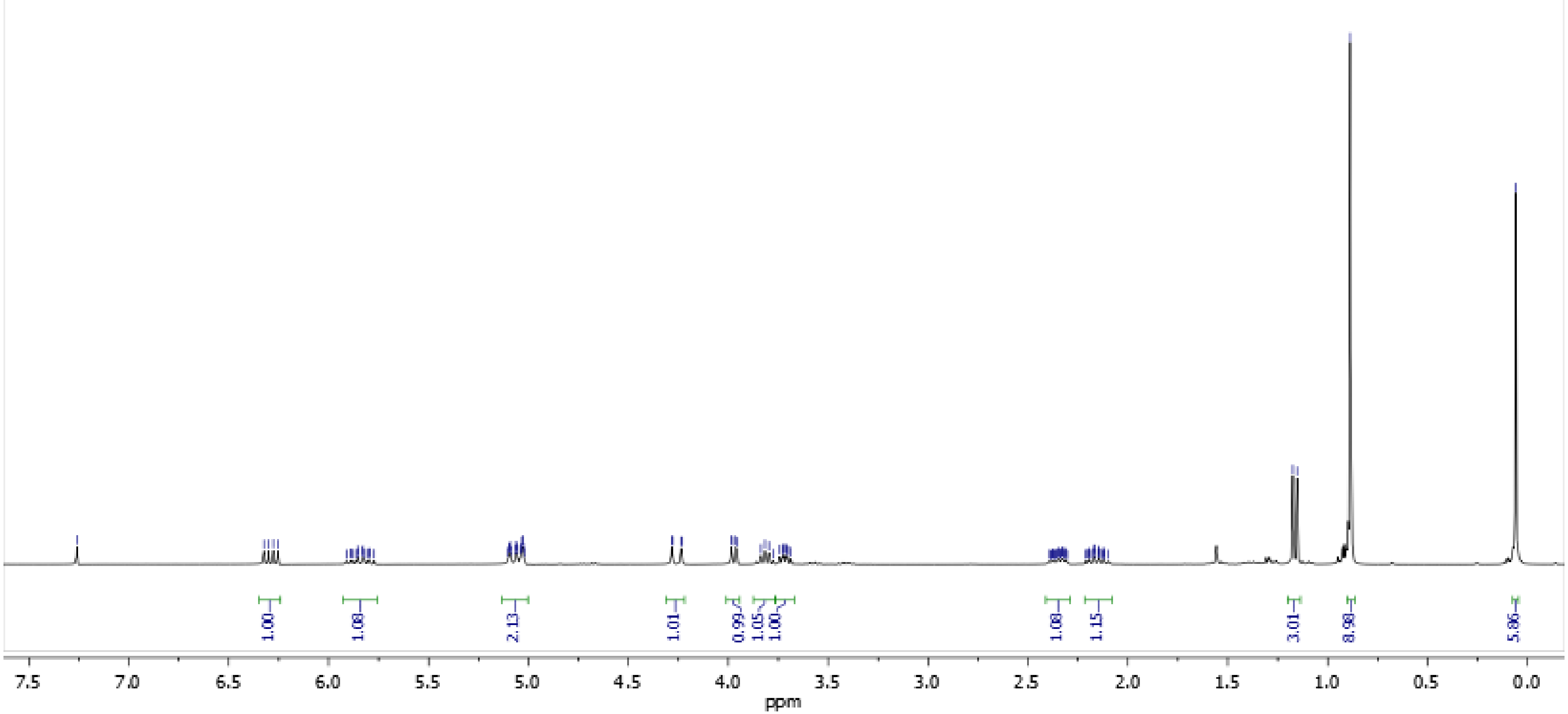

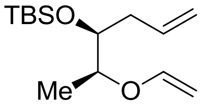

^{13}C NMR of **21** ($CDCl_3$)

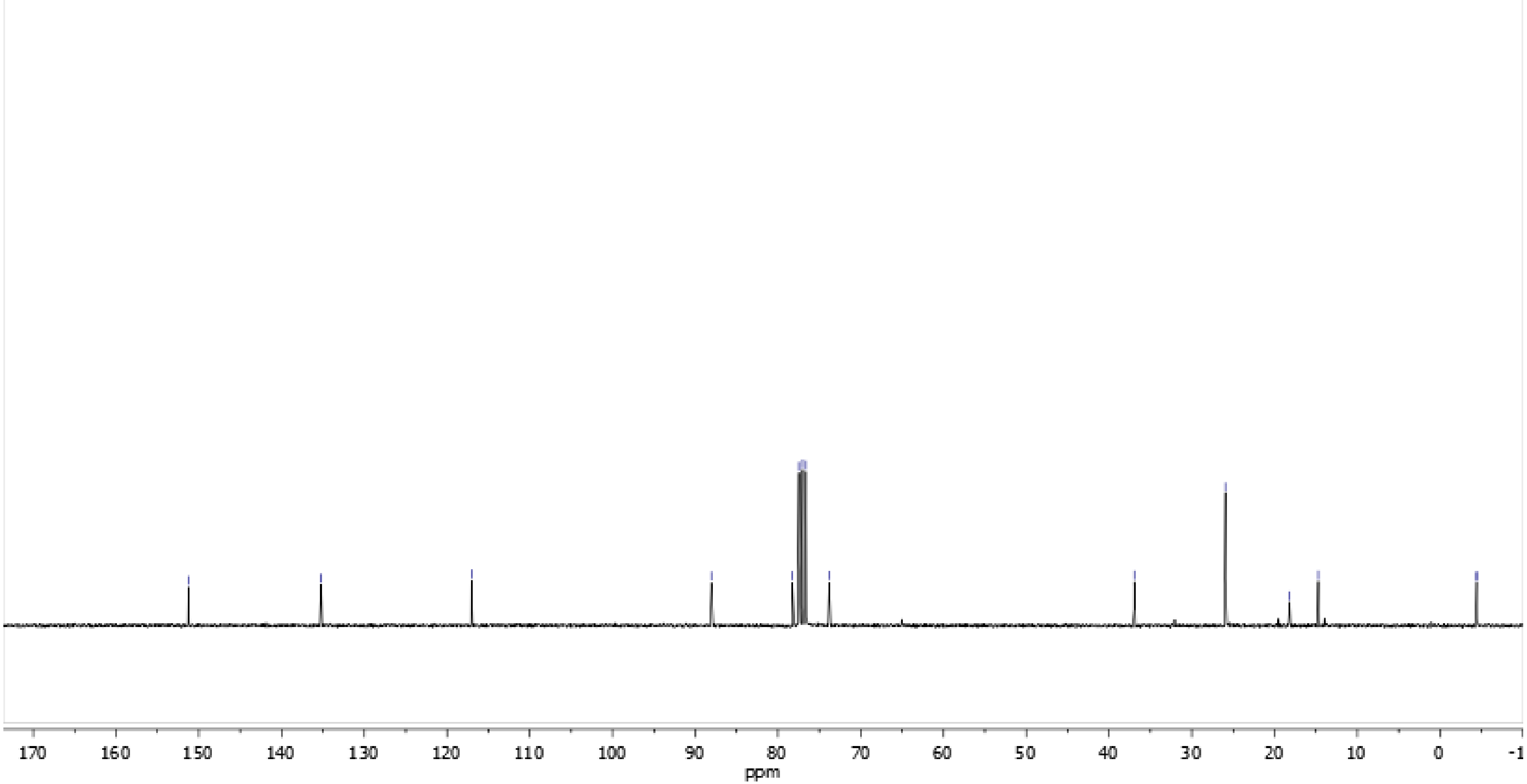

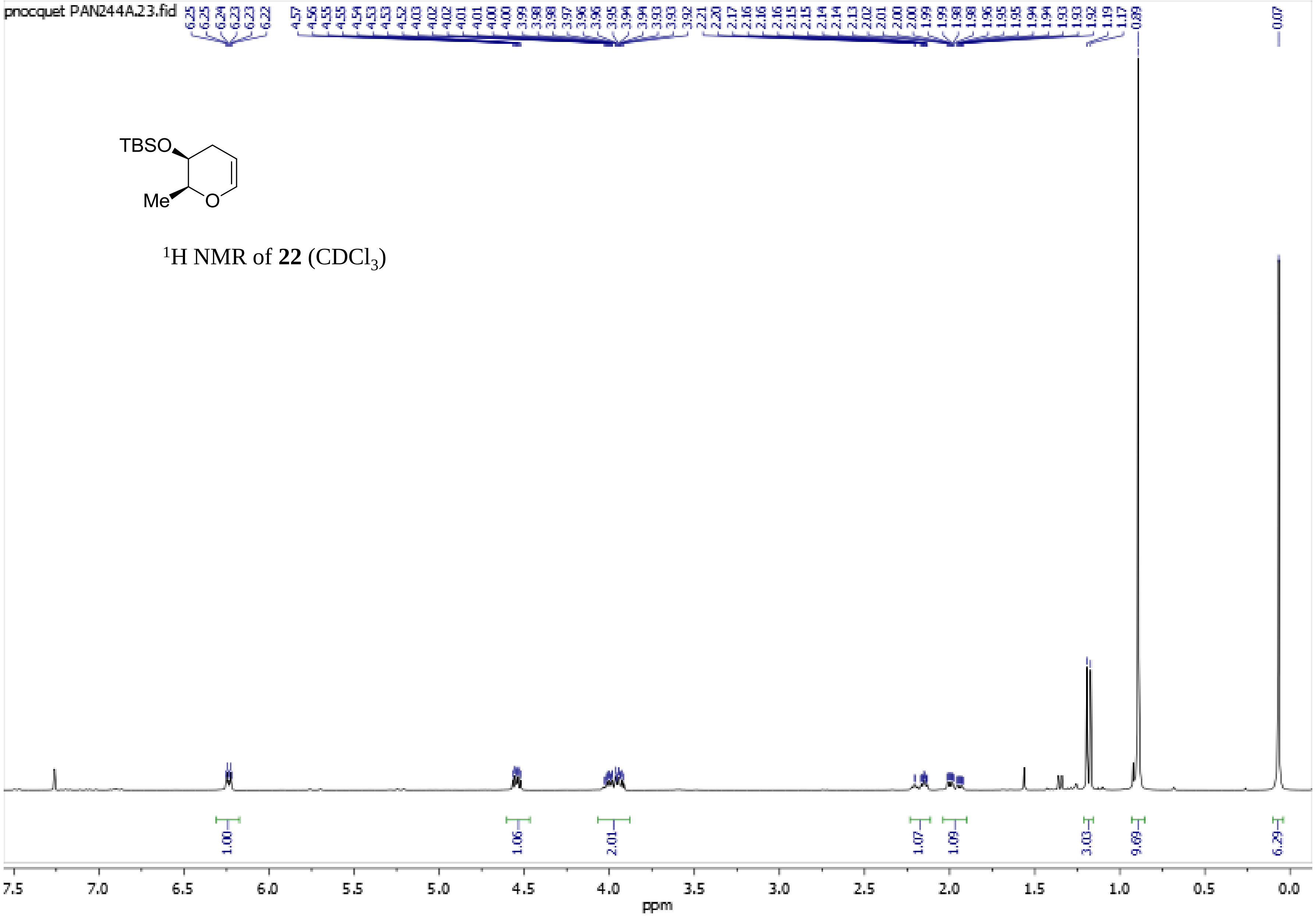

^{1}H NMR of **22** ($CDCl_3$)

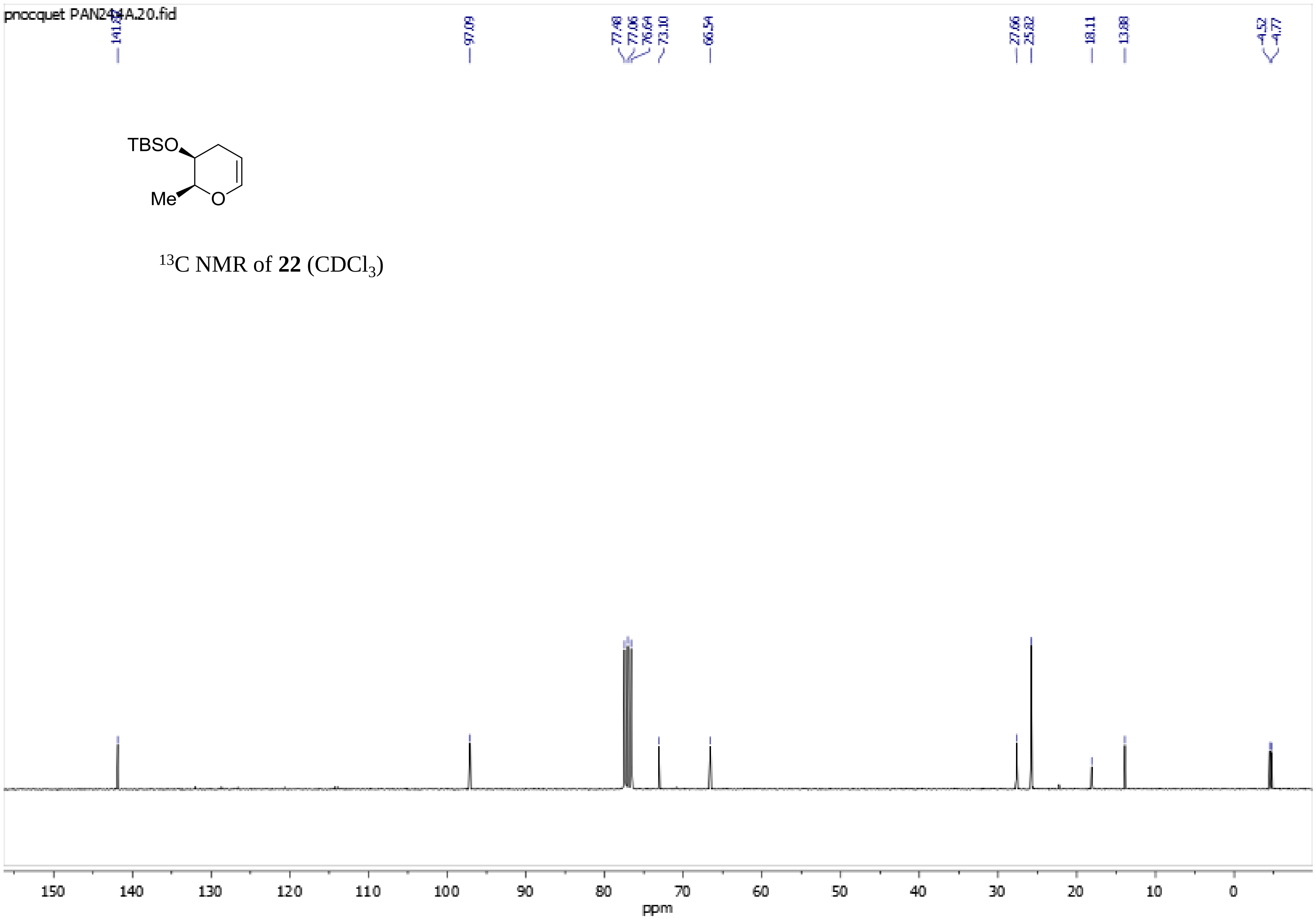

^{13}C NMR of **22** ($CDCl_3$)

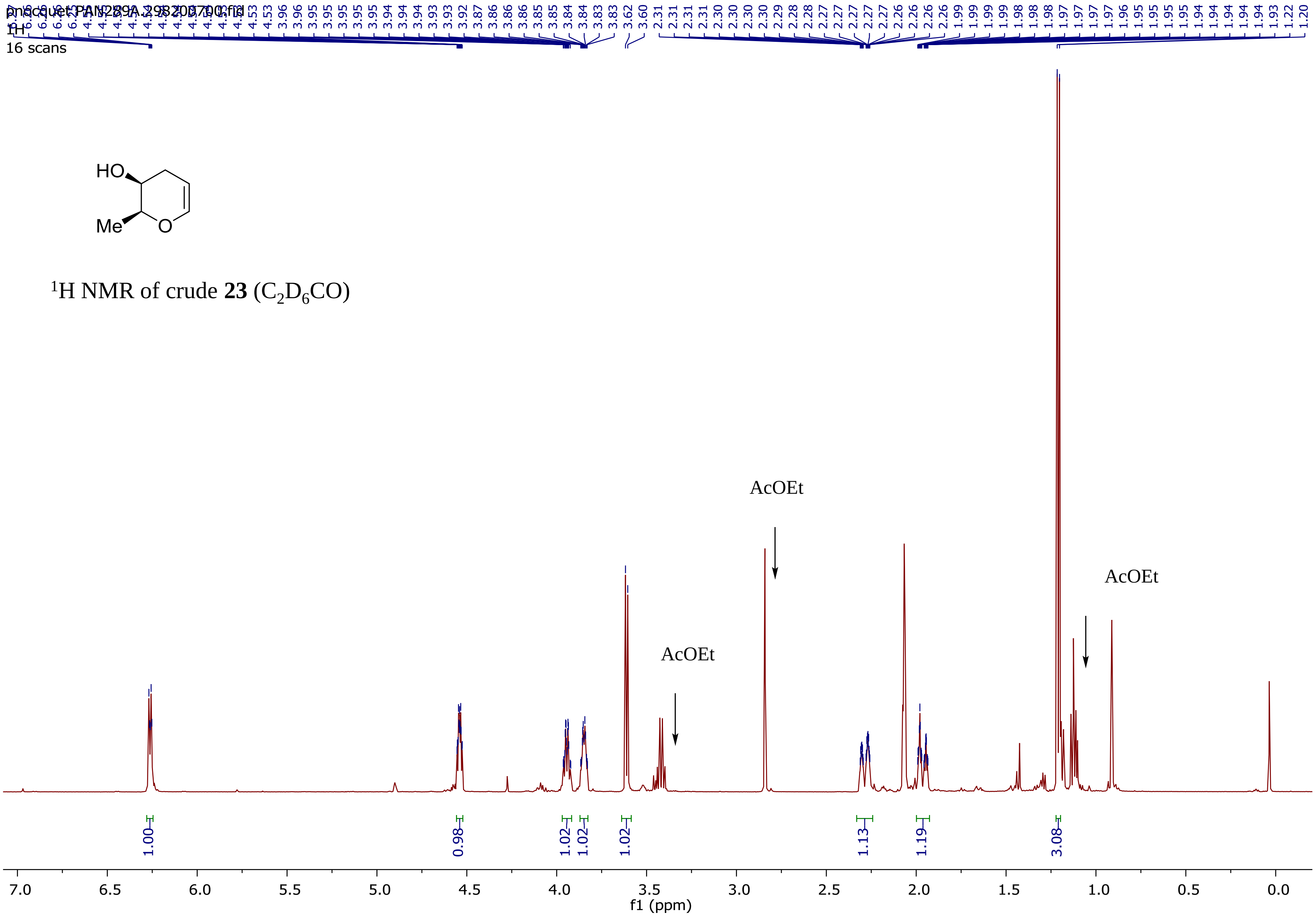

^{1}H NMR of crude **23** (C_2D_6CO)

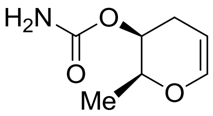

^{1}H NMR of **24** (CD_3OD)

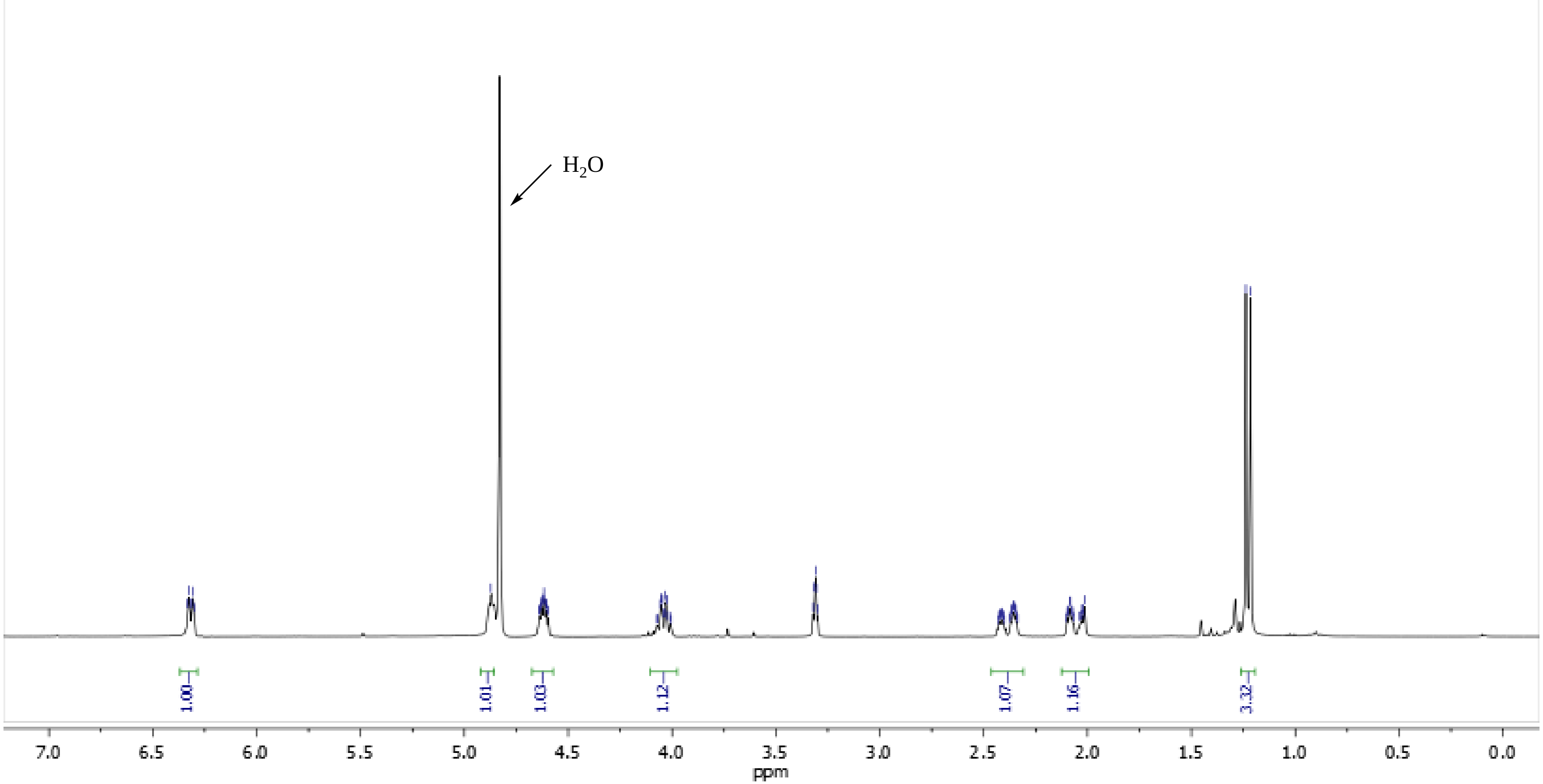

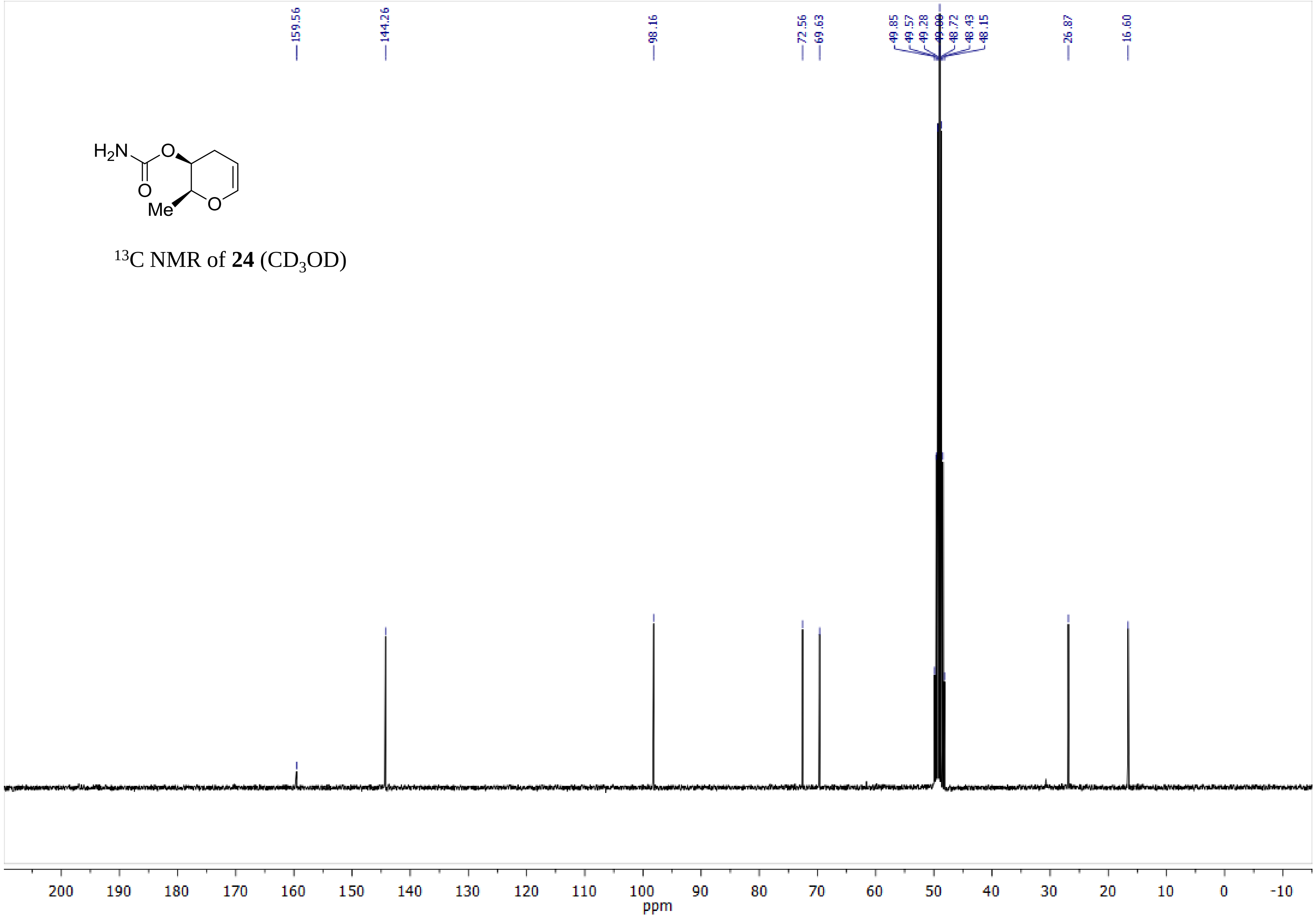

^{13}C NMR of **24** (CD_3OD)

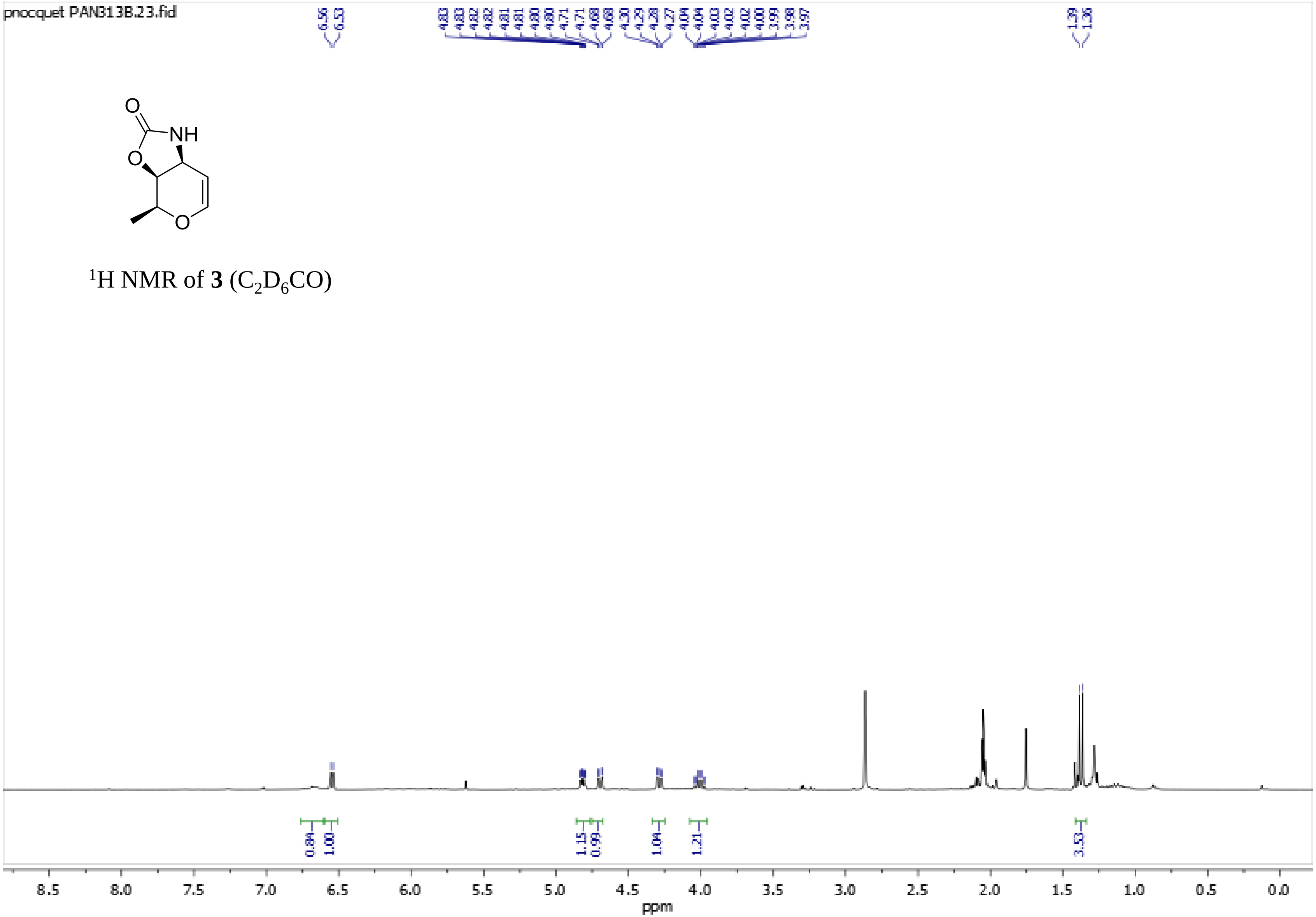

^{1}H NMR of **3** (C_2D_6CO)

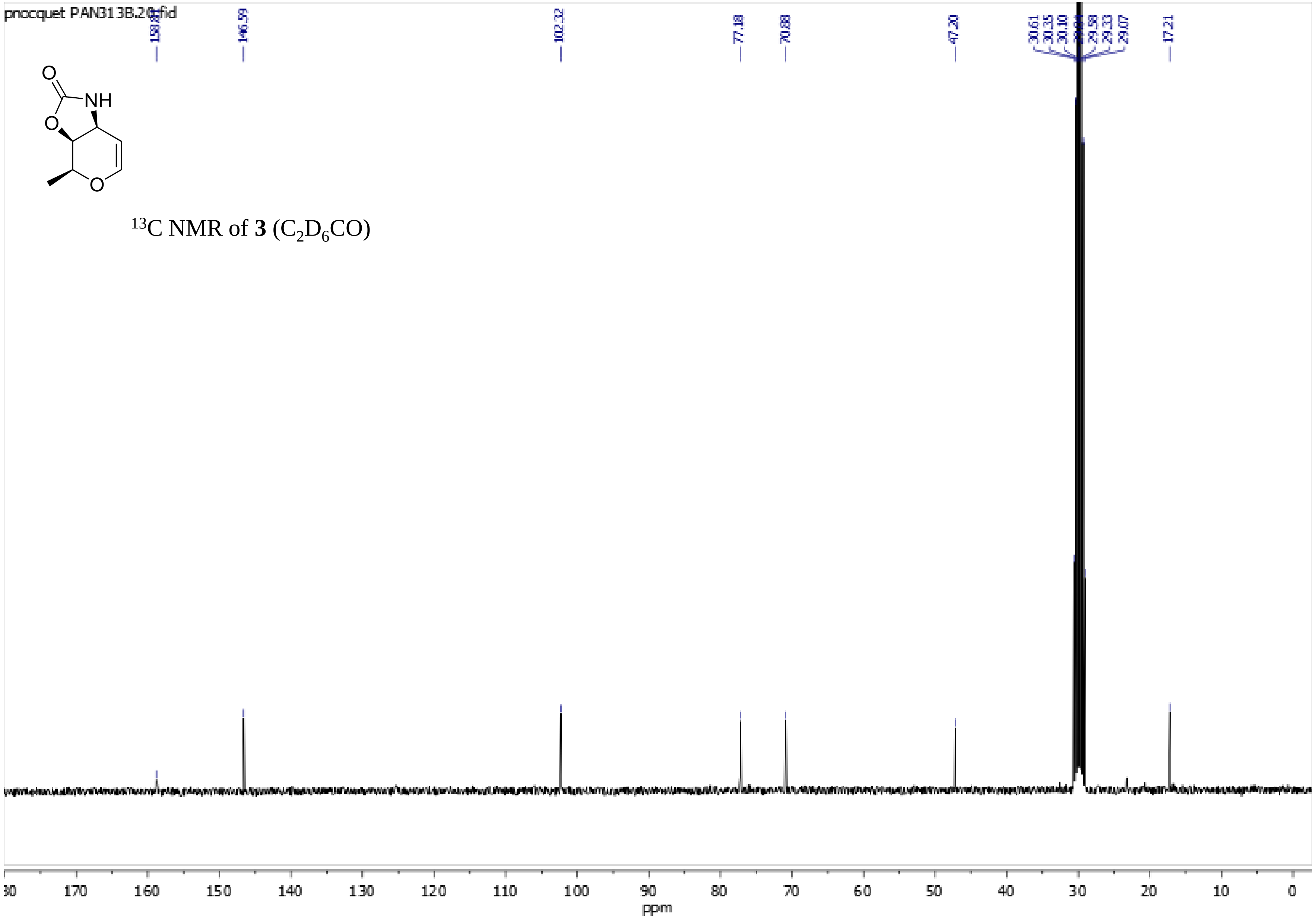

^{13}C NMR of **3** (C_2D_6CO)

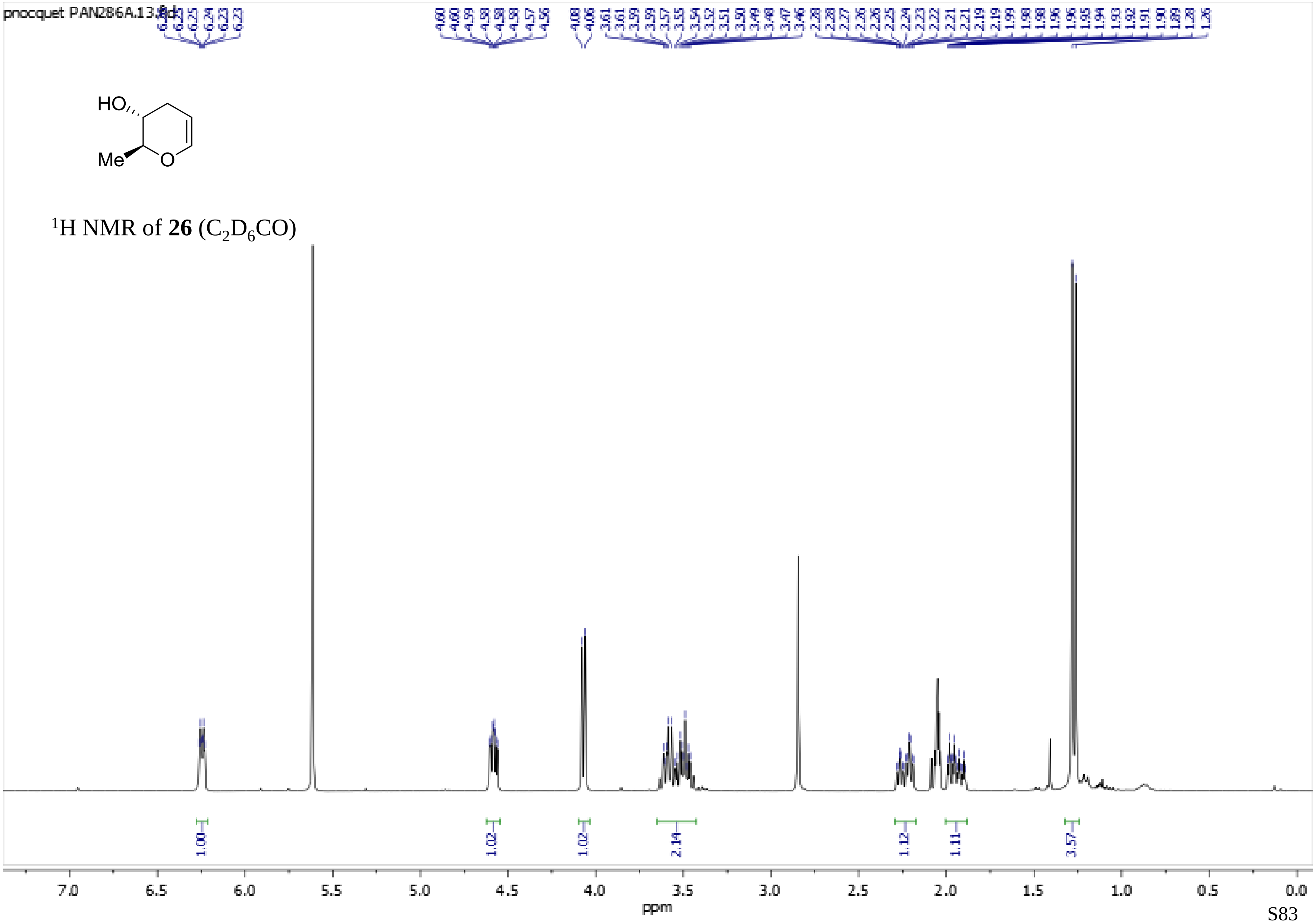

^{1}H NMR of **26** (C_2D_6CO)

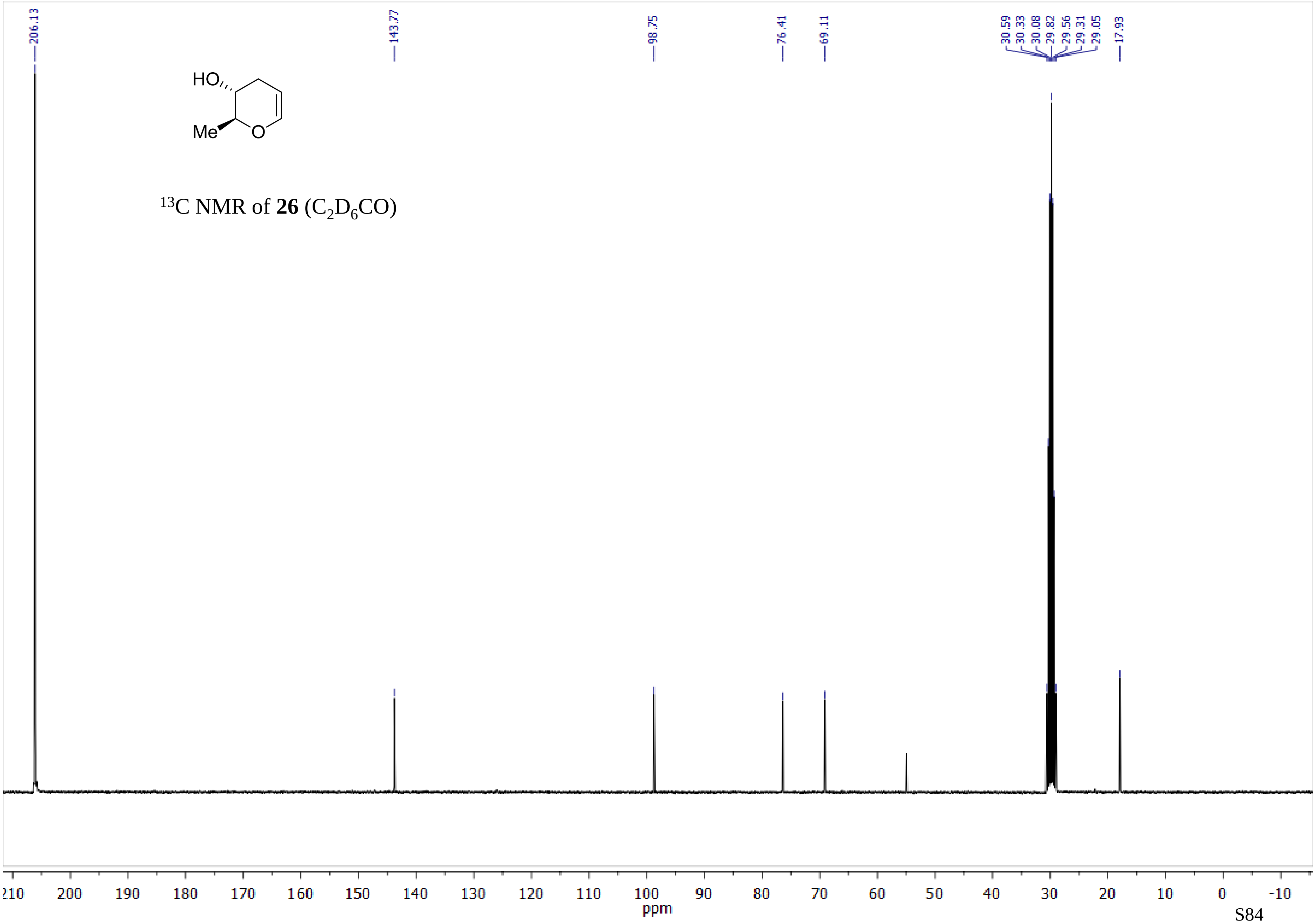

^{13}C NMR of **26** (C_2D_6CO)

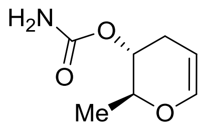

^{1}H NMR of **27** ($CDCl_3$)

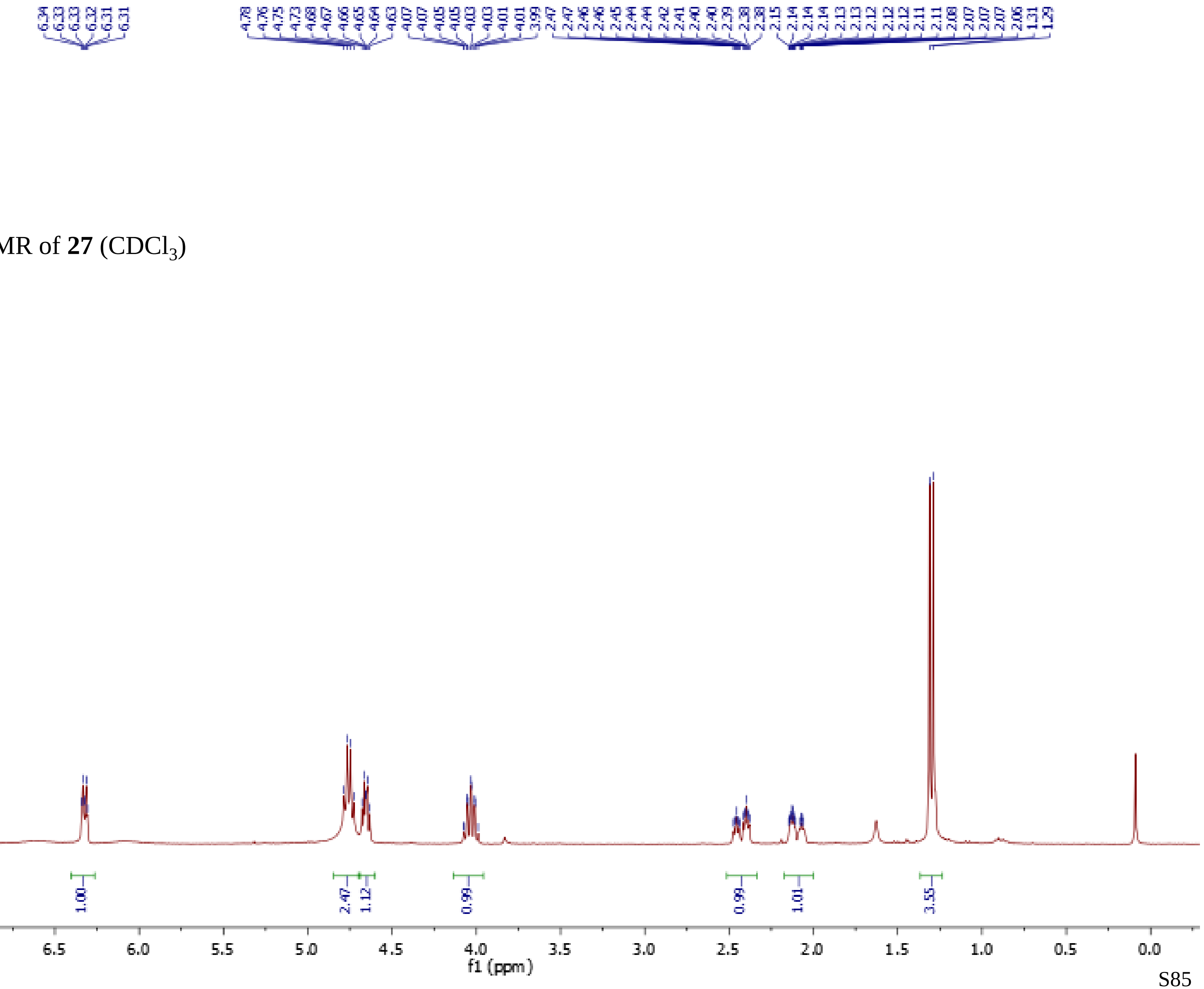

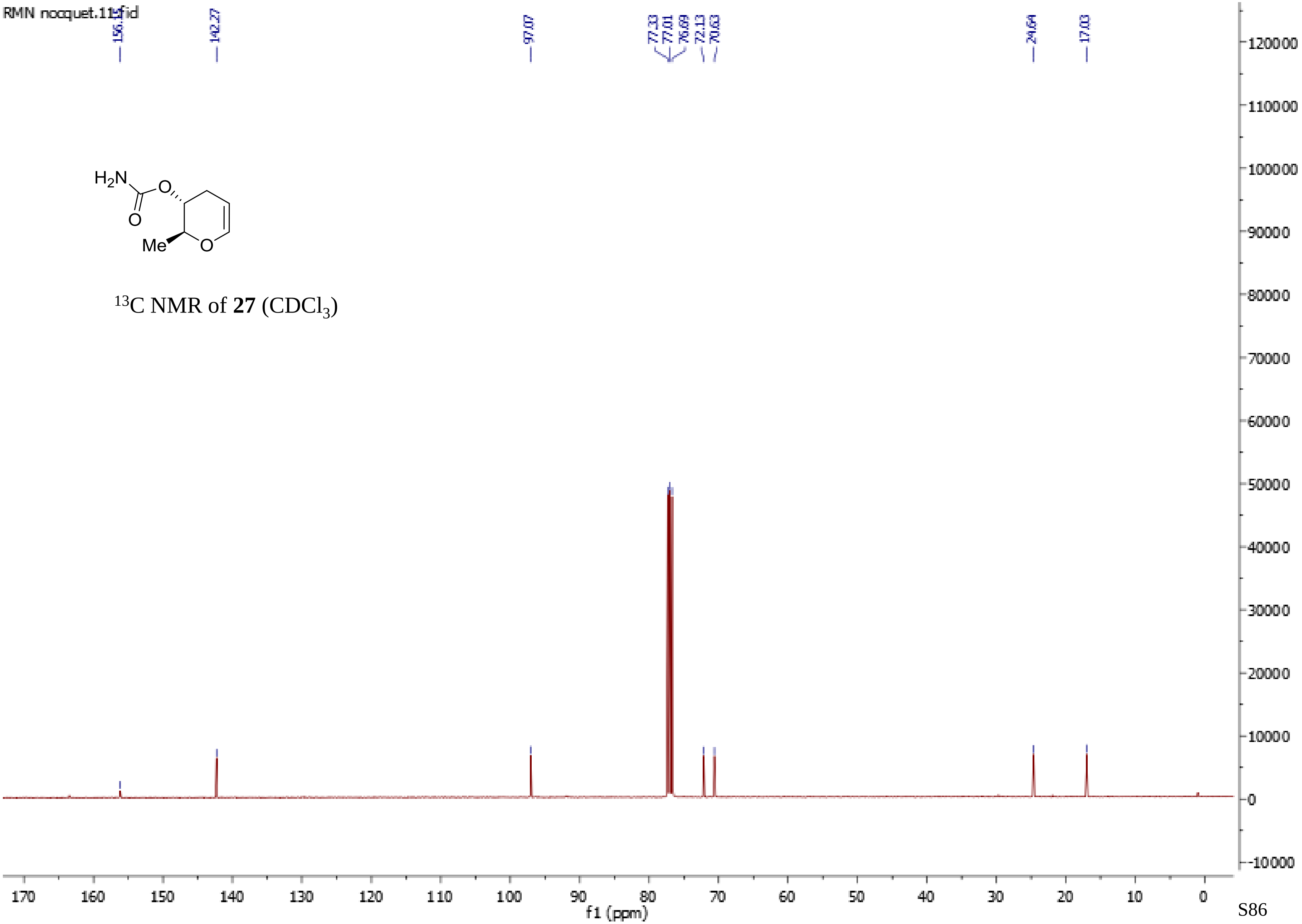

^{13}C NMR of **27** ($CDCl_3$)
